# Transcriptomic and enzymological evidence for plastid peptidoglycan synthesis in the gymnosperm *Picea abies*

**DOI:** 10.1111/tpj.70588

**Published:** 2025-12-06

**Authors:** Yayoi Sugita, Amanda J. Dowson, Ichiro Kajisa, Katsuaki Takechi, Yilan E, Jingzhi Zhao, Jiaqi Wang, Xiaofei Lin, Laura Diaz‐Saez, Adrian J. Lloyd, Christopher G. Dowson, Hiroyoshi Takano

**Affiliations:** ^1^ Graduate School of Science and Technology Kumamoto University Kumamoto 860‐8555 Japan; ^2^ School of Life Sciences University of Warwick Coventry CV4 7AL UK; ^3^ Faculty of Advanced Science and Technology Kumamoto University Kumamoto 860‐8555 Japan; ^4^ College of Life Sciences Inner Mongolia University Hohhot 010021 China

**Keywords:** chloroplast division, Norway spruce (*Picea abies*), peptidoglycan synthesis genes, *Physcomitrium patens*, *Arabidopsis thaliana*, penicillin‐binding protein, MurE ligase

## Abstract

It is understood that a cyanobacterium was the progenitor of plastids and that the biosynthesis of cell wall peptidoglycan was lost during chloroplast evolution. However, accumulated data, especially from the moss *Physcomitrium patens*, suggest that peptidoglycan remains essential for plastid division in some land plants. A fundamental set of peptidoglycan biosynthesis (*Mur*) genes has been identified in the genomes of these land plants, while many angiosperms no longer encode some core *Mur* genes, including a bifunctional penicillin‐binding protein (PBP). Ten incomplete *Mur* genes were previously identified in the genome of the gymnosperm *Picea abies* but these could be pseudogenes or encode proteins that have been repurposed. For instance, mutant albino maize and Arabidopsis seedlings possess a defective UDP‐N‐acetylmuramoyl‐l‐alanyl‐d‐glutamate‐‐2,6‐diaminopimelate ligase (MurE), an intact MurE ligase being essential for peptidoglycan synthesis. In this study, we isolated a full set of cDNAs for peptidoglycan biosynthesis from *P. abies*. GFP fusion proteins with either *P. abies* (Pa)MurE or PaPBP were detected in chloroplasts. Cross‐species complementation assays with *PaMurE* in Arabidopsis albino *MurE* mutants and Physcomitrium *MurE* chloroplast division mutants showed that the gymnosperm *MurE* completely rescued both mutant phenotypes. Enzymatic assay of recombinant PaMurE proteins revealed they catalyze the same reaction performed by their bacterial MurE homologs. Moreover, the expression of the *PaPbp* cDNA partially rescued the giant chloroplast phenotype in the moss *Pbp* knockout line. These results are consistent with the operation of a functional *Mur* gene set in the Norway spruce genome.

## Significance Statement

Peptidoglycan (murein) biosynthesis is involved in chloroplast division in mosses, while Arabidopsis *MurE* mutants are defective in chloroplast development. We have shown that the gymnosperm *Picea abies* expresses 11 genes of the peptidoglycan biosynthesis pathway, and that *P. abies* MurE has a conventional bacterial d,l‐DAP‐incorporating activity and can also complement the Arabidopsis albino phenotype, suggesting *Picea* MurE could be functional in both chloroplast division and plastid development.

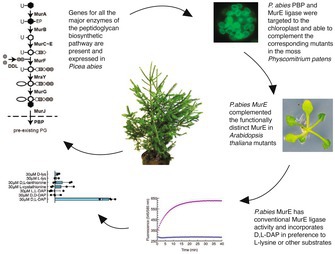

## INTRODUCTION

Peptidoglycan is a continuous covalent macromolecule comprised of a sugar‐peptide polymer and is present in almost all free‐living bacteria, including cyanobacteria (Hoiczyk & Hansel, 2000). Bacterial peptidoglycan confers mechanical resistance to osmotic pressure, maintains cell shape, and functions in cell division (van Heijenoort, 2001). In the primary photosynthetic eukaryotes, such as glaucophytes, red algae, and green plants, it has long been held that only glaucophytes contain a clearly identifiable peptidoglycan between the outer and inner plastid envelopes (Schenk, 1970, Pfanzagl et al., 1996, Iino & Hashimoto, 2003; Sato et al., 2009). However, over the past 25 years, evidence of plastid peptidoglycans has accumulated in green plants, particularly in the moss *Physcomitrium patens* (Hirano et al., 2016; Kasten & Reski, 1997; Machida et al., 2006; Takano & Takechi, 2010; Utsunomiya et al., 2021).

**Figure 1:**
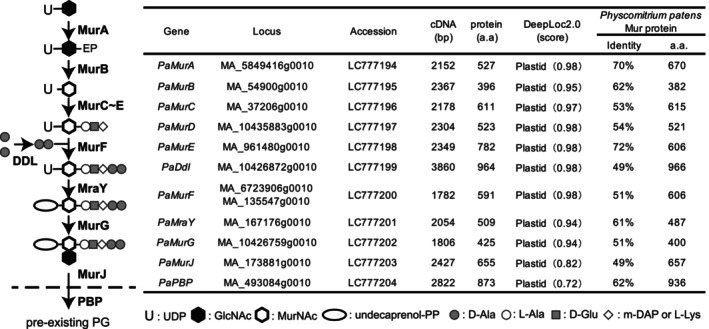
*Mur* genes in *Picea abies*. (Left) Peptidoglycan biosynthesis pathway. The enzymes (bold) MurA and MurB catalyze the formation of UDP‐MurNAc from UDP‐GlcNAc, followed by UDP‐MurNAc‐pentapeptide synthesis by MurC, D, E, and F which append l‐alanine, d‐glutamate, *meso*‐DAP (or l‐lysine), and d‐alanyl‐d‐alanine, respectively. The latter dipeptide is synthesized by d‐Ala: d‐Ala ligase (DDL). MurC–F and DDL all catalyze ATP‐dependent peptide bond formation between two amino acids. MraY and MurG form undecaprenyl pyrophosphoryl GlcNAc‐MurNAc l‐alanyl‐γ‐d‐glutamyl‐meso‐diaminopimelyl‐d‐alanyl‐d‐alanine (lipid II), which is transferred to the periplasm by MurJ. This disaccharide pentapeptide monomer unit is cross‐linked to pre‐existing peptidoglycan; this reaction is mediated by the transglycosylase and transpeptidase activity of penicillin‐binding protein (PBP). (Right) Isolated *Mur* genes from *P. abies*. Locus in the *P. abies* database, accession number, length of isolated cDNA, length of amino acid sequence, localization, and score of DeepLoc2.0 prediction, and amino acid identity (Identity) to that of *Physcomitrium patens* Mur protein with number of amino acids (aa) are shown.

In the genome of *P. patens* (Rensing et al., 2008), we identified 11 *Mur* genes: MurA–MurG, MurJ, MraY, penicillin‐binding protein (PBP), and DDL (*Mur* gene set: Figure 1), all of which are necessary for the primary peptidoglycan biosynthetic pathway (Takano & Takechi, 2010; Utsunomiya et al., 2021). Gene disruption of the *P. patens* (*Pp*) *MurA1/MurA2*, *PpMurE*, *PpMraY*, *PpDdl, PpMurJ*, or *PpPbp* resulted in the appearance of a few macrochloroplasts in each protonemal cell, in contrast to wild‐type (WT) cells that have approximately 40–50 chloroplasts, implying that these *Mur* genes are essential for plastid division in moss (Hirano et al., 2016; Homi et al., 2009; Machida et al., 2006; Utsunomiya et al., 2021). Recently, using a metabolic labeling method for peptidoglycan, we found that plastid peptidoglycan surrounds each chloroplast (Hirano et al., 2016). Single‐pixel densitometry with conventional electron micrographs suggested the presence of moss peptidoglycan in the intermembrane space of the chloroplast envelopes (Sato et al., 2017).

Bacterial MurE enzymes catalyze the aminoacylation of UDP‐N‐acetylmuramoyl‐ l‐alanyl‐d‐glutamate with either 2,6‐diaminopimelic acid or l‐lysine to form the corresponding UDP‐MurNAc‐tripeptide as an intermediate in peptidoglycan biosynthesis (Figure 1). *MurE* genes are generally found in all green plants, including angiosperms (MacLeod et al., 2024; van Baren et al., 2016). Stable transformants of the *PpMurE* knockout line (∆*PpMurE*) expressing *Anabaena* MurE with a plastid‐targeting sequence showed recovery from a macrochloroplast phenotype to the WT chloroplast phenotype, suggesting that moss MurE has a similar function to that of their bacterial counterparts (Garcia et al., 2008). In contrast, Arabidopsis T‐DNA or Ds‐tagged lines for the *MurE* gene (AT1G63680, *atmurE* mutants) displayed a white‐seedling phenotype via decrease of plastid gene expression (shown in Figure 3A), suggesting that *AtMurE* has a different function, related to chloroplast development (Garcia et al., 2008). Moreover, AtMurE could not rescue the macrochloroplast phenotype of ∆*PpMurE*, suggesting that *AtMurE* is functionally divergent from the bacterial and moss MurE proteins (Garcia et al., 2008). Recently, four reports on the cryo‐EM structures of the plastid‐encoded RNA polymerase (PEP) complex from spinach *Spinacia oleracea*, white mustard *Sinapis alba* and *Nicotiana tabacum* indicate that MurE homologs (also identified as PEP‐associated protein 11, PAP11) are a peripheral component of PEP (Vergara‐Cruces et al., 2024, Wu et al., 2024, do Prado et al., 2024; Wang et al., 2024). Additionally, Tran et al. (2023) claimed that AtMurE also supports plastid division due to an absence of clear plastid structures in *atmurE* mutants. Therefore, the role of AtMurE in plastid division is not yet determined.

Physcomitrium PBP is a class A‐type PBP containing a transglycosylase domain and a C‐terminal penicillin‐binding transpeptidase domain (Takahashi et al., 2016). Knockout of *PpPbp* (∆*PpPbp*) also resulted in the appearance of macrochloroplasts as described above (Machida et al., 2006). Stable transformants of ∆*PpPbp* expressing the class A *Anabaena* PBP (AnaPbp, alr5324) with the plastid‐targeting sequence exhibited a partial recovery (Takahashi et al., 2016), suggesting PpPBP interacts more efficiently with the moss plastid division apparatus. Recently, it has been reported that PpPBP interacts with the C‐terminus of the outer envelope plastid division protein PLASTID DIVISION2 (PDV2) in the intermembrane space of plastids (Chang et al., 2024). The interaction of a heterologous cyanobacterial PBP with PDV2 in moss chloroplasts may account for the partial recovery of the WT phenotype.

The accumulation of plant genome sequences has made it possible to infer whether each species has peptidoglycan biosynthesis genes. Further to our review (Takano & Takechi, 2010), the potential existence of 10 *Mur* genes, excluding MurJ, in various plant genomes was investigated by van Baren et al. (2016) and MacLeod et al. (2024). Several prasinophytes; a streptophyte alga, *Klebsomidium nitens*; a liverwort, *Marchantia polymorpha*; a lycophyte, *Selaginella moellendorffii*, and the gymnosperm *Thuja plicata* potentially encode 10 *Mur* genes, similar to the situation in the glaucophyte and *P. patens* genomes. We also identified segments of 10 *Mur* genes in the conifers *P. abies* and *Pinus taeda* in our previous paper (Lin et al., 2017).

In contrast, no angiosperms having a full *Mur* gene set, with a bifunctional ClassA PBP, are known. Generally, many angiosperm genomes, including Arabidopsis, have only *MurE*, *Ddl*, *MraY*, and *MurG* genes, which may have been repurposed by exaptation. In the several angiosperm genomes such as the monocot *Asparagus officinalis* and eudicot *Citrus sinensis*, nine *Mur* genes but not a bifunctional *Pbp* gene were identified. Although a gene encoding only a transglycosylase domain was found in these and a number of important crop genomes (Dowson, 2024; MacLeod et al., 2024), a bifunctional *Pbp* gene encoding both transglycosylase and transpeptidase domains was not found in angiosperms, suggesting that the loss of the capability to transpeptidate peptidoglycan strands may have a significant effect on the role or, even, the presence of peptidoglycan in these plants.

Even if parts of plant genes homologous to each bacterial *Mur* gene were found, it cannot be ruled out that some of these are pseudogenes, as suggested by Björn (2020). To investigate the function of gymnosperm MurE, we isolated an ortholog of MurE from the larch, *Larix gmelinii* (LgMurE, Lin et al., 2017). Cross‐species complementation assay of a T‐DNA‐tagged mutant *atmurE‐1* (SALK_126518) that shows a white‐seedling phenotype (Garcia et al., 2008) showed that the expression of *LgMurE* cDNA completely rescued the Arabidopsis albefaction defects. However, the *LgMurE* gene did not rescue the macrochloroplast phenotype of ∆*PpMurE*. These results suggest that *L. gmelinii* MurE has a similar function to angiosperm MurE, which is distinct from peptidoglycan biosynthesis (Lin et al., 2017). However, preliminary data in our laboratory indicated that *P. abies* (*Pa*) MurE, unlike LgMurE, may function as a conventional MurE ligase, so we determined to clarify whether gymnosperms may indeed retain the capability of synthesizing peptidoglycan. In this study, we isolated a full set of *Mur* cDNA from *P. abies* indicative of a plant capable of synthesizing peptidoglycan. Functional analyses of *PaMurE* and *PaPbp*, by cross‐species complementation and enzymatic analyses, imply that these two enzymes could indeed be part of a functional peptidoglycan synthesis pathway in the gymnosperm Norway Spruce.

## RESULTS

### Isolation of a full set of *P. abies Mur* genes capable of synthesizing peptidoglycan

To isolate a full set of *Mur* genes from *P. abies*, we re‐analyzed the genomic sequence and identified two further fragments with homology to *Mur* genes: MA_10435883g0010 to *MurD* and MA_173881g0010 to *MurJ* (Sundell et al., 2015; https://plantgenie.org). The fragment MA6723906g0010, annotated as PaMurD and also as PaMurF in the previous paper (Lin et al. 2017), has closer homology to MurF. To identify the 5′ or 3′ regions, including the UTR of each gene, a rapid amplification of cDNA ends (RACE) method was used, and then the full‐length cDNAs were cloned for all genes (Figure 1). The cloned *MurF* gene from *P. abies* contained the regions corresponding to MA_6723906g0010 and MA_135547g0010. The subcellular localization prediction software DeepLoc 2.0 (Thumuluri et al. 2022) suggested that the expected amino acid sequences of all 11 proteins were likely to be plastid targeted. High amino acid identities to those of the moss proteins (Figure 1) suggested that these amino acid sequences may be functional Mur proteins. Molecular phylogenetic analysis of each Mur protein showed robust monophyly with other Mur proteins from gymnosperms, indicative that these isolated gene sequences were not contaminants from other organisms (Figures S1 and S2). These results strongly suggested that *P. abies* has a *Mur* gene set capable of synthesizing plastid peptidoglycan. However, the possibility that these genes were functionally inactive with respect to peptidoglycan biosynthesis could not be ruled out. MurE was of particular interest as it has been identified as having a distinct, albino knockout phenotype in *Zea mays* and Arabidopsis (Garcia et al. 2008; Williams‐Carrier et al., 2014), whereas the moss knockout phenotype is the generation of macrochloroplasts (Machida et al. 2006). PBP was likewise of interest as the transpeptidase activity typical of a class A PBP appears to have been lost from the transglycosylase proteins of angiosperms. For this reason, we focused on *MurE* and *Pbp* genes isolated from *P. abies*.

### Subcellular localization of PaMurE and PaPBP

**Figure 2:**
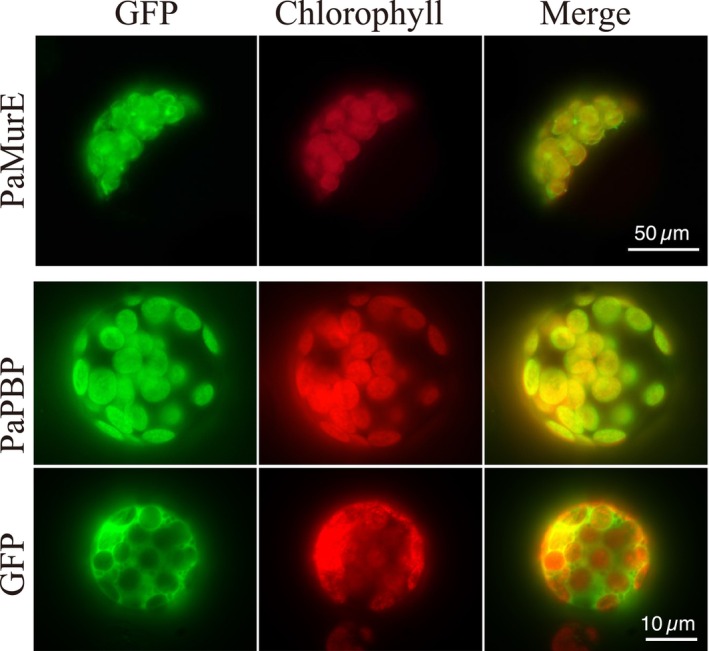
Subcellular localization of PaMurE‐ or PaPBP‐GFP fusion proteins. Transient expression of the putative transit peptide of PaMurE‐GFP in Arabidopsis protoplasts (upper). Transient expression of the full‐length PaPBP‐GFP (middle) or GFP (lower) in moss protoplasts. Fluorescent images of GFP, chlorophyll autofluorescence, and merged images are shown.

To determine the subcellular localization of the PaMurE protein, we constructed a plasmid expressing the N‐terminal 111 amino acids of PaMurE fused with sGFP(S65T), driven by the CaMV 35S promoter. By introduction of the recombinant plasmid DNA into protoplasts of *Arabidopsis thaliana*, GFP fluorescence was observed in chloroplasts, corroborating the computational predictions (Figure 2).

To determine the subcellular localization of PaPBP, we used the full‐length sequence and constructed plasmids expressing PaPBP‐GFP fusion proteins; protoplasts of the moss *P. patens* were transformed using polyethylene glycol. PaPBP‐GFP fusion proteins localized to the chloroplast, and accumulated toward the chloroplast envelope (Figure 2).

### Complementation of the albino phenotype of an Arabidopsis *MurE* knockout mutant with *P. abies MurE*

**Figure 3:**
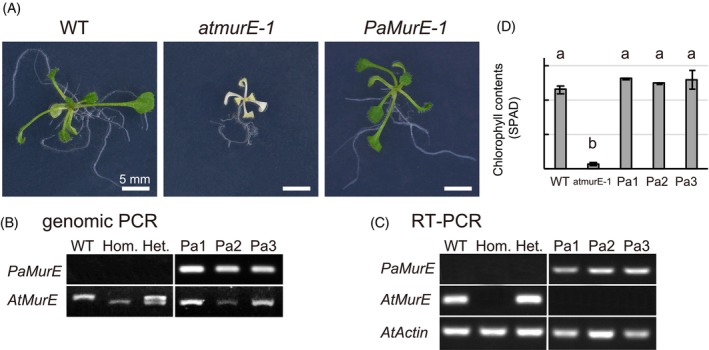
Complementation assay of *atmurE‐1* with *Picea abies MurE*. (A) Two‐week‐old plants of the wild type (WT), the *atmurE‐1* showing a white‐seedling phenotype and transgenic plant of *atmurE‐1* mutants expressing *PaMurE* (*PaMurE‐1*) are shown. (B) Genomic PCR of three complementation lines (PaMurE1–PaMurE3) shows that they were homozygous for *AtMurE* mutation (right). For control, WT, homozygous (Hom.), and heterozygous (Het.) T‐DNA‐tagged plants for *AtMurE* were shown in the left. (C) RT‐PCR were performed to confirm the expression of the *PaMurE* and *AtMurE* genes. *Atactin2* was used as an internal control. (D) The chlorophyll contents of the fifth leaves from 40‐day‐old WT, the *atmurE‐1* mutant and *PaMurE*‐expressing lines were measured with a SPAD 502 Chlorophyll Meter. Data are expressed as mean ± SD; different letters indicate significant differences at *P* < 0.05.

To analyze whether PaMurE has the same function as AtMurE, a complementation assay of *atmurE‐1* was performed with a *PaMurE* gene. The constructed plasmid pBI101‐PaMurE containing the *PaMurE* cDNA driven by the CaMV35S promoter was introduced into plants with heterozygous *atmurE‐1* T‐DNA insertion via *Agrobacterium*‐mediated transformation, because homozygous *atmurE‐1* plants have a white seedling phenotype (Figure 3A). Genomic PCR was used for screening homozygous *atmurE‐1* plants with the *PaMurE* gene (Figure 3B). Expression of the introduced *PaMurE* and absence of expression of *AtMurE* was confirmed by reverse transcription‐polymerase chain reaction (RT‐PCR) in three homozygous *atmurE‐1* transformants (Pa1–Pa3 in Figure 3C). Transgenic *atmurE‐1* plants expressing *PaMurE* cDNA showed a WT phenotype with recovery of chloroplast greening (Figure 3A,D) These results showed that expression of *PaMurE* significantly rescued the albino phenotype in the *atmurE‐1* mutant, suggesting that PaMurE has a function complementary to that of AtMurE.

### Complementation of the giant chloroplast phenotype of a moss *MurE* knockout mutant with *P. abies MurE*

**Figure 4:**
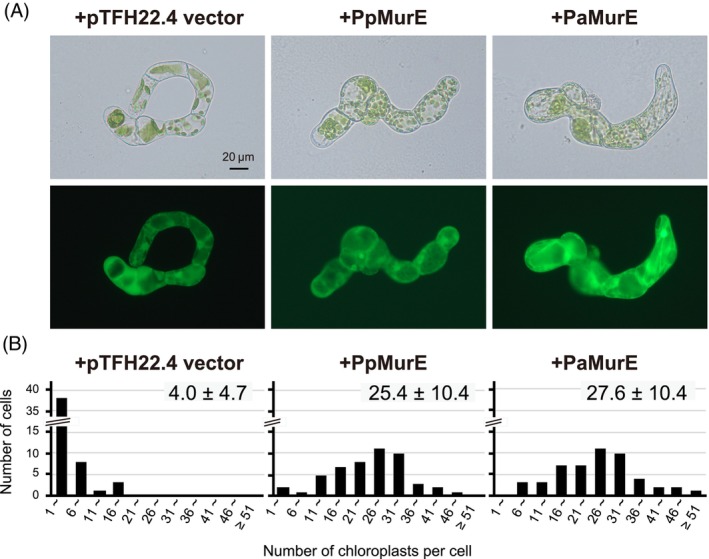
Transient complementation assay of the *PpMurE* knockout line with *Picea abies MurE*. The plasmids containing a gene for the *PpMurE* or *PaMurE* were constructed from the pTFH22.4 vector including the GFP gene, which is independently driven by the CaMV 35S promoter. Each plasmid was introduced into the protoplasts of the ∆*PpMurE* line, and DNA‐induced cells were identified by GFP fluorescence under microscopic observation. (A) Protonemal cells generated from protoplasts of the *PpMurE* knockout line transformed with pTFH22.4 vector, *PpMurE* or *PaMurE* are shown. In the lower panels, GFP fluorescent images of the same cells are shown. (B) The number of chloroplasts in cells with GFP fluorescence in each complementation test (*n* = 50) were counted. The mean chloroplast numbers are also shown.

To analyze whether PaMurE is capable of substituting for moss PpMurE in peptidoglycan biosynthesis, a complementation assay of the moss deletion strain ∆*PpMurE* was performed by transient expression of *PaMurE* cDNA. The plant vector pTFH22.4*‐PaMurE*, with *PaMurE* under the control of the CaMV 35S promoter, was introduced into protoplasts of ∆*PpMurE* by PEG‐mediated transformation and transformants detected by the independent expression of *gfp* under a second 35S promoter within the vector. Moss cells with GFP fluorescence at 5 days after DNA transfer were identified, and the number of chloroplasts per cell was counted (Figure 4). Protonemal cells generated from protoplasts of ∆*PpMurE* expressing the pTFH22.4 vector had only four chloroplasts per cell, whereas ∆*PpMurE* expressing *PpMurE* or *PaMurE* cDNA showed complementation with approximately 25 chloroplasts per cell. The non‐parametric Kruskal–Wallis test revealed a significant difference in chloroplast numbers between ∆*PpMurE* expressing the vector and *PaMurE* cDNA, confirming PaMurE can function in peptidoglycan biosynthesis.

### Enzymatic activity of PaMurE and LgMurE proteins

To determine whether PaMurE or LgMurE can catalyze the same reaction as bacterial MurE, recombinant PaMurE and LgMurE were expressed in *Escherichia coli*. Recombinant proteins were designed to initiate at the predicted chloroplast transit peptide cleavage site (as indicated by ChloroP, DeepLoc 2.0 and sequence alignments), designated PaMurE‐TP and LgMurE‐TP, respectively, or to additionally eliminate the amino terminal Streptophyte‐specific domain (SATD) (as determined by alignment to the bacterial MurE ligases), designated PaMurE‐SATD and LgMurE‐SATD. In the SATD, several disordered regions were predicted by the flDPnn program (Hu et al., 2021). Proteins were expressed with different tags: an amino terminal hexahistidine (His), a hexahistidine with an Avi tag (His_Avi), or a carboxy terminal Avi tag with a hexahistidine (Avi_His).

**Figure 5:**
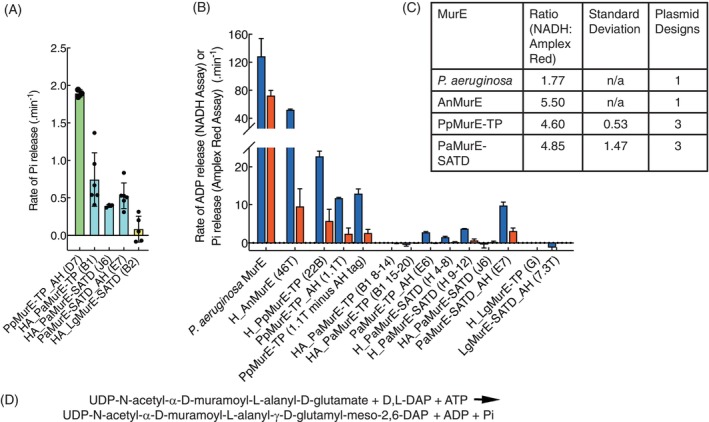
MurE‐dependent generation of ADP or phosphate (Pi) from *Pseudomonas aeruginosa*, Anabaena PCC7120, *Physcomitrium patens*, *Picea abies*, and *Larix gmelinii* MurE. (A) MurE activities of proteins expressed in baculovirus‐infected *Spodoptera frugiperda* (Sf9) insect cells. Activities were measured by Amplex Red assay and error bars are the standard deviation. (B) MurE activities of proteins expressed in *Escherichia coli* as determined by both NADH assay (blue bars) and Amplex Red assay (red bars). Rates were determined using coupling enzymes to relate the MurE products, ADP and Pi, to the consumption of NADH at *A*_340_ or the increase in fluorescence of resorufin from Amplex Red, respectively. Data were calculated as μmoles of product per μmole MurE ligase per min. Results are from triplicate samples for the NADH assay and from six to nine replicates for the Amplex Red assay and error bars represent standard deviation. (C) Comparison of the rates determined by the two assays are presented as a ratio: NADH (ADP):Amplex Red (Pi) for a single protein preparation for each of the two bacterial samples: *Pseudomonas aeruginosa* MurE and AnMurE (46T) and three protein preparations for each of the two plant enzymes: PpMurE‐TP (22B, 1.1T and 1.1T minus His tag) and PaMurE‐SATD (E7 and two S200 fractions of H). (D) Equation for UDP‐N‐acetylmuramoyl‐l‐alanyl‐d‐glutamate‐‐2,6‐diaminopimelate ligase. Names of protein tags have been abbreviated: His to H, His_Avi to HA, and Avi_His to AH and figures in parentheses represent construct names with, where applicable, fraction numbers from S200 gel filtration.

Most plasmid constructions of LgMurE and PaMurE were very weakly expressed in *E. coli*, particularly when the SATD was present in the coding sequences, possibly because of interference with host cell peptidoglycan synthesis, so a eukaryotic expression system (baculovirus‐infected *Spodoptera frugiperda* (Sf9)) was also used. Purified proteins (Figure S3) were analyzed for UDP‐Mur*N*Ac‐tripeptide ligase activity using two distinct low‐volume assays: a fluorescence assay that couples the production of inorganic phosphate to the conversion of Amplex Red to resorufin and a spectrophotometric assay that couples the production of ADP to the oxidation of NADH (Figure S4a,b) (Dowson et al., 2022). These assays monitored MurE ligase activity using d, l‐diaminopimelate (d, l‐DAP) and UDP‐N‐acetylmuramoyl‐ l‐alanyl‐d‐glutamate as substrates (Figure 5D), as we had previously identified d, l‐DAP as the preferred diamino substrate for *P. patens* MurE (Dowson et al., 2022).

The MurE proteins extracted from *S. frugiperda* were less active than their *E. coli*‐expressed counterparts (Figure 5A,B). Despite the low activities, data from the *S. frugiperda* extracted proteins confirmed the ability of three PaMurE constructions, with deletions of either the transit peptide alone (‐TP) or both the transit peptide and the streptophyte‐specific amino terminal domain (‐SATD), to catalyze the ligation of d, l‐DAP to UDP‐N‐acetylmuramoyl‐l‐alanyl‐d‐glutamate (Figure 5A; Figure S5).

Three similar permutations of LgMurE ligase, when purified from either *S. frugiperda* or *E. coli*, did not function in these assays as conventional MurE ligases (Figure 5A,B). The most consistently active gymnosperm MurE ligase construction was PaMurE‐SATD, purified from either *S. frugiperda* or *E. coli* with His or His_Avi tags at the amino terminus, or an Avi_His tag at the carboxy terminus (Figure 5A,B). Although *S. frugiperda*‐expressed and purified His_Avi_PaMurE‐TP had some weak activity, the same protein was inactive when expressed in and purified from *E. coli*. PaMurE‐TP_Avi_His expressed from *E. coli* was active but significantly less than the PaMurE‐SATD_Avi_His protein (unpaired *t* test *P*_0.05_ with the difference and standard error of the mean being: 6.974 ± 0.5508).

The two active plant MurE enzymes, from *P. patens* and *P. abies*, were an order of magnitude less active than *Pseudomonas aeruginosa* MurE and several fold less active than the cyanobacterial AnMurE (Figure 5B) when assayed under the conditions specified. However, the gymnosperm‐derived PaMurE‐SATD_Avi_His was comparable in specific activity to the moss‐derived protein PpMurE‐TP_Avi_His (Figure 5B). As the moss *PpMurE* gene used here encodes no recognizable SATD, PpMurE‐TP is a protein of similar size to PaMurE‐SATD as well as bacterial MurE homologs (Figure S6).

Previous data for PpMurE, specifically His_PpMurE‐TP (Dowson et al., 2022) and in Figure 5B for PpMurE‐TP_Avi_His, indicate that the removal of either an amino terminal His or a carboxyterminal Avi_His tag, respectively, by TEV protease cleavage followed by reverse immobilized metal affinity column purification, did not increase PpMurE activity. However, data here suggest that the amino terminal His_Avi tag has a detrimental effect on the activity of the PaMurE protein, both PaMurE‐TP and PaMurE‐SATD (Figure 5B).

Although the pattern of activities of the different proteins is maintained between the two assays enzyme turnover was 1.8–5.5‐fold more in the NADH spectrophotometric assay than in the Amplex Red fluorescence assay, especially for the cyanobacterial and plant enzymes, where there was a greater difference (Figure 5C). The NADH assay detects ADP release and recycles it to ATP, preventing the accumulation of the ADP product, unlike the Amplex Red assay where inorganic phosphate is part of the coupling reaction and ADP accumulates (Figure S4). The apparent difference between the two enzyme systems and an observed inhibitory effect of ADP on AnMurE in the Amplex Red assay lead us to propose that product inhibition plays a more major role in the activity of the plant and cyanobacterial enzymes compared to *P. aeruginosa* MurE.

MS/MS analysis of the gel‐extracted trypsin digests of cyanobacterial and plant MurE ligases, expressed in either baculovirus‐infected Sf9 insect cells or *E. coli*, failed to identify any one post‐translational modification that might explain the significant differences in activity between the different proteins and the two hosts (Figure S7).

### Proteins copurifying with streptophyte MurE ligases including SATD

It was evident that the presence of the amino terminal SATD had not only a negative effect on PaMurE activity in the *in vitro* assays it also contributed to the instability of the proteins in both heterologous expression systems. Notably, these TP‐less proteins consistently copurified with other proteins and nucleic acid, especially when expressed in *E. coli* (Figure S3b, vi–viii and xiii) and it may be that the binding of one or more of these contributes to the reduced activities of the SATD‐containing proteins. LC–MS analysis of proteins extracted from SDS‐PAGE gel slices identified two major copurifying proteins, common not only to the *L. gmellini* and *P. abies* MurE purifications but also to other streptophyte MurE ligases with a SATD. One, the bifunctional polymyxin resistance protein, ArnA, in plants is homologous to the monofunctional enzymes UDP‐d‐xylose synthase and UDP‐glucuronic acid decarboxylase both of which utilize UDP‐glucuronate as a substrate. The other glucosamine‐6‐phosphate synthase, GlmS, is important in bacteria as an intermediate enzyme in the formation of UDP‐GlcNAc, the substrate of both MurA and MurG in peptidoglycan synthesis. GlmS shares some sequence homology to ArnA (14.2% identity over 75.8% coverage as calculated by EMBL:EBI Mview https://www.ebi.ac.uk/jdispatcher/msa/mview).

The remaining contaminating proteins included the chaperone HtpG and a FKBP‐type peptidyl‐prolyl cis‐trans isomerase (SlyD), both of which assist protein folding, and the 50S ribosomal protein L2 (rplB). Both SlyD and the ribosomal protein L2 have sufficient adjacent basic residues that their presence may be due to an affinity for the Ni resin. It was observed that as well as protein chaperones there was a preponderance of proteins important for not only transcription but also translation. These included *E. coli* RNA polymerase subunit ß' (rpoC) which was not unexpected since an interaction between angiosperm MurE homologs and the plastid RNA polymerase ß' SI1 domain and a PAP1 SAP motif (associated with DNA binding) within the PEP complex is partly mediated through sequences within the MurE carboxy terminal domain (Vergara‐Cruces et al., 2024). MurE (identified as PAP11 in the PEP complex) has consequently been implicated in plastid transcription initiation as well as positioning the PAP1‐SAP domain at the DNA entrance channel (Vergara‐Cruces et al., 2024). Whereas no direct interaction of PAP11 with RNA has been reported, it is closely positioned to PAP2 which has 18 pentatricopeptide repeats (PPRs), implicated in RNA binding and processing, and proximal to the RNA exit channel (Vergara‐Cruces et al., 2024; Wu et al., 2024). The evident frequency of ribosome‐related proteins in our samples, prepared not only from an *E. coli* expression host but also *S. frugiperda*, may be indicative that the role of PAP11 extends to an involvement of the MurE SATD with the PAP2 RNA processing function.

### Substrate specificity of PaMurE

**Figure 6:**
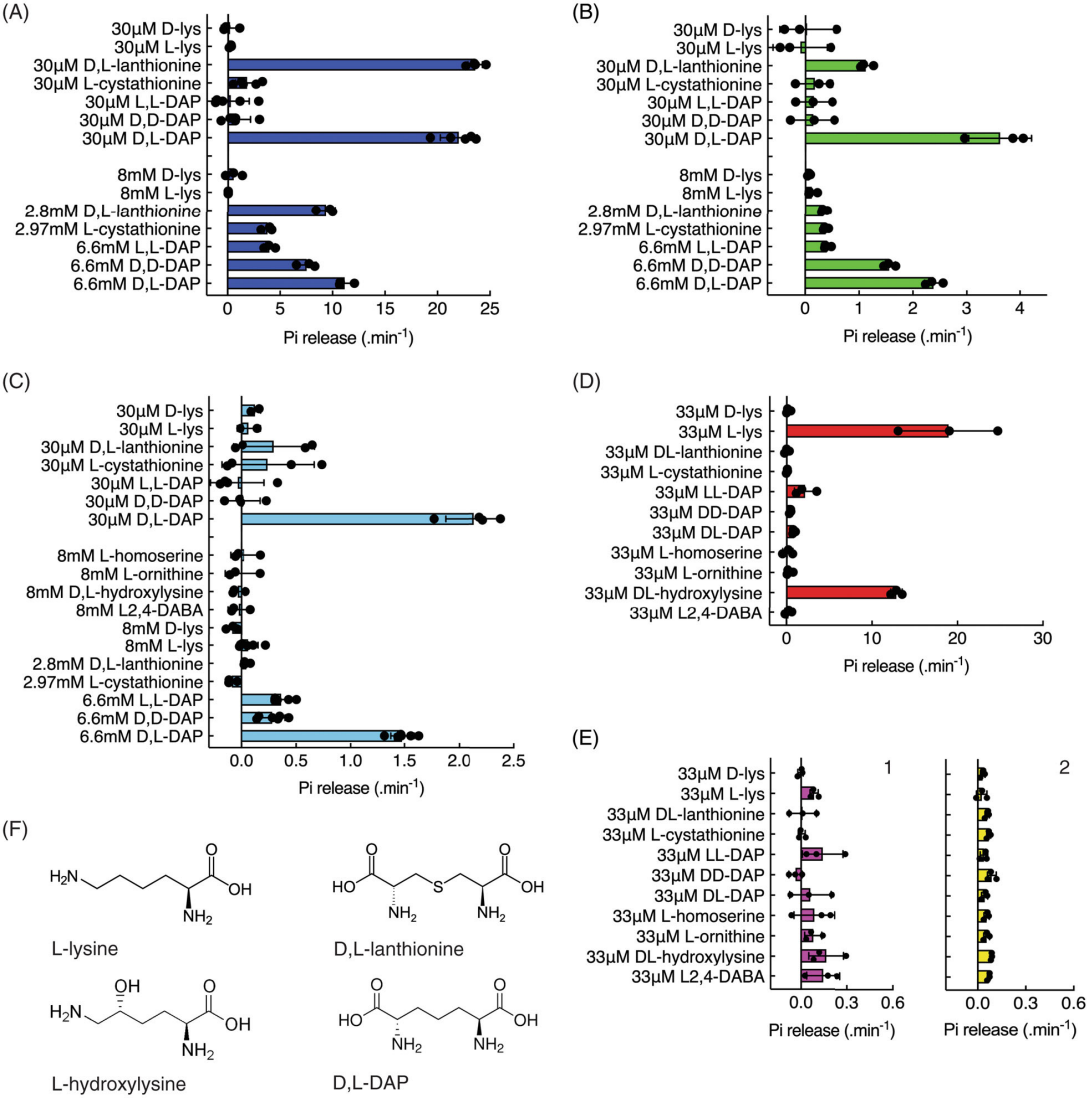
Amino acid substrate preference for bacterial and plant MurE ligases with UDP‐N‐acetylmuramoyl‐l‐alanyl‐d‐glutamate as the recipient molecule. Activities of proteins were analyzed by detection of inorganic phosphate using a coupled reaction where Amplex Red is converted to the fluorescent product resorufin (Figure S4). Proteins were expressed in *Escherichia coli* (A, B, C, D, E2) or baculovirus‐infected Sf9 insect cells (E1) and purified as described. (A) His_AnMurE (46T), (B) PpMurE‐TP_Avi_His (1.1T) and (C) PaMurE‐SATD_Avi_His (E7), (D) *Streptococcus pneumoniae* MurE and (E1) His_Avi_LgMurE‐SATD (B2) and (E2) LgMurE‐SATD_Avi_His (7.3T), and (F) major potential amino acid substrates and their molecular structures. Activities were first determined with the amino acid substrate in excess (2.8–6.6 mM) and then with the amino acid limited to 30–33 μM and error bars are the standard deviation.

A variety of potential MurE amino acid substrates were tested to characterize the substrate specificity of PaMurE. Typically, the substrate of the MurE of Gram‐negative bacteria is d, l‐DAP, which is also the predominant substrate for both the moss, PpMurE, and cyanobacterial, AnMurE, ligases (Dowson et al., 2022). Of all the amino acids tested d, l‐DAP was the preferred substrate for PaMurE‐SATD_Avi_His, with d, d‐DAP and l, l‐DAP being incorporated only when in considerable excess at 8 mM, but at 30 μM d, l‐DAP ligase activity on the l, l and d, d stereoisomers was not significant (Figure 6C). All other substrates tested, including l‐Lys, the major substrate for the MurE of Gram‐positive bacteria, were not incorporated, with the exception of l‐cystathionine and, particularly, d, l‐lanthionine, which were weak substrates for the plant enzymes (Figure 6B,C). Interestingly, d, l‐lanthionine was equally as efficiently incorporated as d, l‐DAP by *Anabaena* MurE, a facility it appears to have in common with *E. coli* MurE (Mengin‐Lecreulx et al., 1994) (Figure 6A). These data are indicative of a difference between the active sites of the cyanobacterial and plant enzymes, with the gymnosperm PaMurE being the most selective.

LgMurE‐SATD, prepared from either baculovirus‐infected insect cells or *E. coli*, was likewise tested on the same potential substrates. It was concluded that His_Avi_LgMurE‐SATD had no significant activity as there was no significant difference between substrates (anova, *P*_0.05_), and, although there was a significant difference between the means for LgMurE‐SATD_Avi_His, the activities were very low and could not be reliably attributed to MurE ligase activity due to slight background contamination of the enzyme with inorganic phosphate (e1 and e2, respectively). *Streptococcus pneumoniae* MurE was included as a control, to validate detection of activity of lysine‐incorporating MurE ligases, and was determined to be able to utilize both l‐hydroxylysine and l‐lysine as substrates with UDP‐N‐acetylmuramoyl‐l‐alanyl‐d‐glutamate as the acceptor.

### Complementation of the giant chloroplast phenotype of a moss *PBP* knockout mutant with *P. abies PBP*

**Figure 7:**
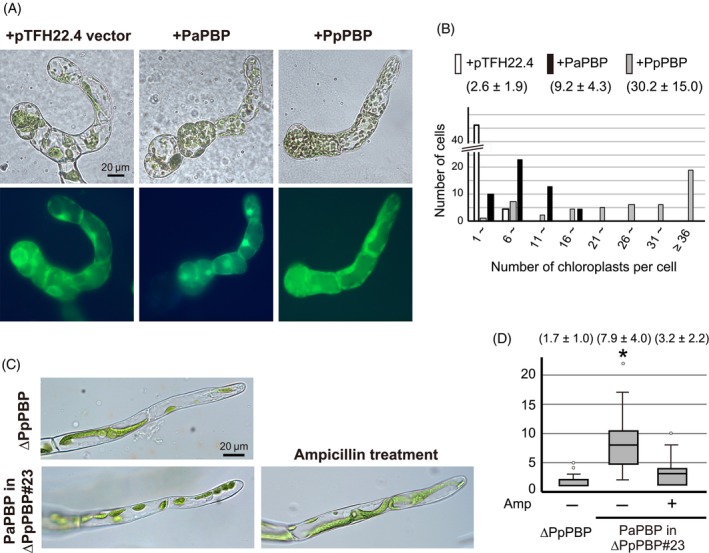
Complementation assay of the *PpPbp* knockout line with *Picea abies Pbp*. (A) The pTFH22.4 vector and derived plasmid containing the *PaPbp* or *PpPbp* gene was introduced into the protoplasts of the ∆*PpPBP* line. DNA‐induced cells were identified by GFP fluorescence. In the lower panels, GFP fluorescent images of the same cells are shown. (B) Number of chloroplasts per cell in the transient expression experiments shown in (A) (*n* = 50). The mean chloroplast numbers are shown in parentheses. Any combinations of the three data are statistically significant using a non‐parametric Kruskal–Wallis test (*P* < 0.01). (C) Protonemal cells of the ∆*PpPbp* line and stable transformant line #23 expressing *PaPbp* are shown in the left. Cells of the transformant #23 treated with ampicillin for 2 weeks is on the right panel. (D) Box plot of the chloroplast numbers in the stable transformants shown in (C) (*n* = 50). The mean chloroplast numbers are shown in parentheses. Asterisk indicates that the value is significantly different from that of the ∆*PpPbp* plants according to the non‐parametric Kruskal–Wallis test (*P* < 0.01).

Among seed plants, class A *PBP* genes were only found in the gymnosperm genomes. To analyze whether isolated PaPBP could work as a class A PBP in the peptidoglycan biosynthesis pathway, with both transglycosylase and transpeptidase activities, a transient assay for complementation of ∆*PpPbp* was performed with *PaPbp* cDNA driven by the CaMV 35S promoter. The constructed plasmid pTFH22.4‐PaPbp was introduced into protoplasts of the moss knockout mutant ∆*PpPbp*, and regenerated cells were observed under a microscope 5 days after DNA induction (Figure 7A,B). Transformed cells were identified by GFP fluorescence. Protonemal cells generated from protoplasts of ∆*PpPbp* expressing the control pTFH22.4 vector had only 2.6 ± 1.9 chloroplasts per cell, while ∆*PpPbp* cells expressing the *PpPbp* cDNA showed complementation with 30.2 ± 15.0 chloroplasts per cell. In contrast, the ∆*PpPbp* expressing *PaPbp* cDNA had 9.2 ± 4.3 chloroplasts per cell. In comparison with the control, this chloroplast number is statistically significant using a non‐parametric Kruskal–Wallis test (*P* < 0.01), suggesting partial complementation with *PaPbp*.

To confirm partial recovery of giant chloroplast phenotypes, we generated stable transformants expressing *PaPbp* cDNA from the ∆*PpPbp* line. The number of chloroplasts in chloronemal subapical cells of the transformant showed a slight increase to 7.9 ± 4.0 per cell, also indicative of a partial complementation with *PaPbp* (Figure 7C,D). Then, we examined the effect of ampicillin on chloroplasts of ∆*PpPbp* expressing *PaPbp* (Figure 7C,D). The average chloroplast number of these cells treated with ampicillin for 2 weeks decreased to 3.2 ± 2.2 chloroplasts per cell, suggesting that enzymatic activities of PaPBP were inhibited by β‐lactam antibiotics.

## DISCUSSION

In this study, we were able to isolate cDNAs encoding transcripts without frameshifts or non‐sense mutations for all *Mur* genes from *P. abies*. The encoded proteins, representing a full set of *Mur* genes, were predicted to be localized in plastids by the DeepLoc 2.0 program, indicative of a pathway with the capability to synthesize peptidoglycan in *P. abies* plastids. Recently, many gymnosperm genomes have been reported (reviewed in Wan et al., 2022). By database searches, we found that the genome of the cedar *Cryptomeria japonica* (Fujino et al., 2023) has a full set of open reading frames for all *Mur* genes (Table S2; Figures S1 and S2). All proteins were predicted to have a plastid targeting sequence and showed high amino acid identities to the moss proteins (Table S2a). In addition, parts of all genes were found in the sequenced gymnosperm genomes of *P. taeda*, *T. plicata*, and *Sequoia sempervirens* (Lin et al., 2017; Zimin et al., 2017; Shalev et al., 2022; Neale et al., 2022; Table S2b). All *Mur* genes except one: *MurC*, *MurC*, *MurJ, MurA*, or *MurE* were found in the genomes of *Pinus lambertiana*, *Abies alba*, *Larix kaempferi*, *Pseudotsuga menziesii*, and *Taxus chinensis*, respectively (Crepeau et al., 2017; Neale et al., 2017; Mosca et al., 2019; Xiong et al., 2021; Sun et al., 2022; Table S2b). On the other hand, in the genome of *Gnetum montanum* (Wan et al., 2018), we detected only parts of two genes (TnS000918299t06 for MurE and TnS000918479t01 for MurG). As transcriptomic and genomic analyses of gymnosperms progress, whether all gymnosperms have all *Mur* genes will become apparent. It is possible that some clades of gymnosperms have lost essential *Mur* genes.

PaMurE recovered the two distinct phenotypes of the Arabidopsis and Physcomitrium mutants, lack of chloroplast differentiation and generation of macrochloroplasts, respectively (Figures 3 and 4). These results suggest that PaMurE can act both in chloroplast development from proplastids and in chloroplast division via peptidoglycan biosynthesis, although they did not directly demonstrate that PaMurE performs both functions in *P. abies* cells. Previously (Lin et al., 2017), we reported that *L. gmelinii* MurE completely rescued the Arabidopsis white seedling phenotype of *atmurE*, but did not recover the macrochloroplast phenotype of ∆*PpMurE*. One possible explanation for the discrepancy between the abilities of PaMurE and LgMurE to complement ∆*PpMurE* could be that Norway spruce and larch have different chloroplast division mechanisms. However, this seems unlikely because both species are in the family Pinaceae; moreover, the MurE ligase domains of the proteins share 96.1% amino acid identity. Because purified larch MurE did not demonstrably function in peptidoglycan biosynthesis when tested with potential substrates including d, l‐DAP and l‐Lys (Figure 6E) this system may not be essential for plastid division in gymnosperms. Conversely, if the peptidoglycan biosynthesis pathway is essential in all gymnosperms, then *L. gmelinii* may encode an alternatively spliced mRNA or an alternative *MurE* gene to the one so far identified.

Although we have demonstrated here that PaMurE has the capacity to function as a conventional MurE ligase, it is less apparent what enzymatic function the streptophyte SATD‐containing MurE homologs would have where the genome does not encode the majority of the Mur enzymes, especially those in the early steps of peptidoglycan synthesis: MurA–MurD. In these plants the d, l‐DAP substrate would be expected to be available, being a precursor in the pathway to l‐lysine (Hudson et al., 2006), but it is improbable that UDP‐MurNAc‐Ala‐Glu could be synthesized, implying these MurE proteins may have an alternative but similar catalytic function. Or they could simply act as a convenient and flexible scaffold in the PEP complex, although the retention of many important catalytic residues including the d, l‐DAP‐specific DNPR (or, commonly, a DNPK) motif is observed in most streptophytes, with a few exceptions, including the cereals (DNPA) and the Podocarp gymnosperms (DNSR). For many angiosperms the MurE residues implicated in interactions with the MurNAc sugar are the least conserved, with the cereals being particularly divergent. Nevertheless, perhaps the best indication that these MurE homologs may have a catalytic function is preliminary X‐ray crystallographic data (to 3.6 Å) for Arabidopsis MurE‐SATD which indicates this protein closely aligns with the *Mycobacterium tuberculosum* and *E. coli* MurE crystal structures.

In addition to *MurE*, Arabidopsis also possesses the *Ddl*, *MraY*, and *MurG* genes. Two T‐DNA tagged lines of the *AtDdl* gene show no defects in chloroplast morphology (Hirano et al., 2016), but this gene may be involved in d‐amino acid metabolism. MurG is known to share sequence similarity with the enzyme synthesizing monogalactosyldiacylglycerol (MGDG), a major plastid lipid (Botté et al., 2005). MraY and MurG may have a role in plastid envelope stability or synthesis. Whether these three genes could have been exapted for secondary functions in gymnosperms remains to be elucidated.

Cross‐species complementation assays showed that expression of the *PaPbp* cDNA partially rescued the giant chloroplast phenotype of ∆*PpPbp*. Because both the transglycosylase and transpeptidase functions in PBP are essential for plastid division (Takahashi et al., 2016), PaPBP would be expected to possess both functions. In Takahashi et al. (2016), we reported that the *Anabaena* PBP with the plastid (chloroplast)‐targeting sequence of PpPBP at its amino‐terminus (CP‐AnaPBP) also showed partial complementation of the ∆*PpPbp* macrochloroplast phenotype. The average chloroplast number in a CP‐AnaPBP stable transformant line of ∆*PpPbp* was 10.3 ± 3.2, similar to that in *PaPbp* complement lines. Recently, it was reported that moss PpPBP directly interacts with PpPDV2, an outer envelope component of the chloroplast division complex (Chang et al., 2024). The reason why neither PaPBP nor AnaPBP completely rescues the ∆*PpPbp* phenotype may be due to less than optimal protein–protein interactions.

Whether peptidoglycan is essential for gymnosperm plastid division has yet to be elucidated. Treatments with peptidoglycan biosynthesis inhibitors have suggested the presence of a peptidoglycan‐associated plastid division system in bryophytes, lycophytes and some algae (Takano & Takechi, 2010), but there are no data for gymnosperms. To address this issue, antibiotic experiments on *P. abies* would be informative, but experiments with *P. abies* plants have so far proven inconclusive due to slow growth rates and the fact that cultured cells typically contain undifferentiated plastids, not chloroplasts. Nevertheless, we are engaged in trying to generate green, cultured *P. abies* cells with high proliferation rates for this purpose. In this context, it is relevant to note that the activity of PaPBP was sensitive to ampicillin (Figure 7). Additionally, for gymnosperms, CRISPR/Cas9‐mediated targeted mutagenesis has been reported in *Picea glauca*, *Pinus radiata*, and Japanese cedar (Cui et al., 2021; Nanasato et al., 2021; Poovaiah et al., 2021). If the *P. abies PBP* and *Mur* gene functions could be similarly modified, it should be possible to directly examine the knockout phenotype of the peptidoglycan biosynthetic enzymes: specifically, the appearance of giant chloroplasts would strongly indicate that peptidoglycan retains a role in plastid division. Moreover, interfering with the function of the *PaMurE* gene would allow us to investigate not only its relationship with plastid division, but also any involvement with chloroplast differentiation, likely mediated through the chloroplast PEP complex.

## MATERIALS AND METHODS

### Plant materials

Norway spruce of approximately 75 cm height was purchased from a local nursery in Japan and grown in standard soil in a controlled environment at 23°C and under continuous illumination. The fresh needles of *P. abies* were collected at Hohhot Arboretum in Inner Mongolia.

Protonemata of the moss *Physcomitrium* (formerly *Physcomitrella*) *patens* (Hedw.) Mitten (Cove‐NIBB WT strain) (Ashton & Cove, 1977; Medina et al., 2019; Sakakibara et al., 2014) were grown on BCDAT medium solidified with 0.8% (w/v) agar in a regulated chamber at 25°C under continuous light (35 μmol photons m^−2^ sec^−1^). The *PpMurE* and *PpPBP* knockout lines (∆*PpMurE* and ∆*PpPBP*) were generated in the previous study (Machida et al., 2006).

Surface‐sterilized seeds of *A. thaliana* ecotype Columbia (WT) and the T‐DNA‐tagged mutant (*atmurE‐1*, SALK_126518; Machida et al., 2006) were grown on solid Murashige‐Skoog (MS) medium under 75 μmol photons m^−2^ sec^−1^ at 23°C under a 16‐h light/8‐h dark cycle. Then, seedlings were transplanted into pots with nutrient soil and grown under the same conditions.

### Isolation of *Mur* genes from *P. abies*

Using the Primescript II 1st standard cDNA synthesis kit with the Oligo dT primer (Takara Bio Inc., Shiga, Japan), cDNA was synthesized from 1 μg of total RNA isolated from leaves of *P. abies* plants using the ISOSPIN plant RNA kit with Assist Buffer (Nippon Gene Co., Ltd., Tokyo, Japan). cDNA fragments for each *Mur* gene except *MurE* were amplified by PCR using the appropriate gene‐specific primer set (Table S1). For 3′ RACE, cDNA was synthesized by using an Oligo dT‐Adaptor Primer (Takara Bio, RNA PCR kit). PCR was performed using the M13 primer M4 (Takara Bio, RNA PCR kit) and a gene‐specific primer, and then DNA fragments amplified by nested PCR with the M13 primer M4 and another gene‐specific primer were cloned and sequenced. To determine the 5′ end of each gene transcript, 5′ RACE was carried out with the SMARTer RACE 5′/3′ kit (Takara Bio). Finally, each full‐length cDNA was amplified, cloned, and sequenced.

For isolation of *MurE*, DNA and RNA were purified from the needles of *P. abies* grown at Hohhot Arboretum. The downstream region of MA_961480g0010 (https://plantgenie.org, Lin et al., 2017) was isolated using the Genome Walking kit (Takara Biomedical Technology, Beijing, China) with three primers: PaMurE‐F1, PaMurE‐F2, and PaMurE‐F3. The ORF for *MurE* was identified with the online software (http://www.bioinformatics.org). To isolate cDNA for the CDS of *PaMurE*, 1st‐strand cDNA was synthesized from 500 ng of total RNA with oligo(dT) adaptor primer (TransGen Biotech Co., LTD, Beijing, China). Then, PCR was carried out by using the TransStart Taq DNA polymerase (TransGen Biotech) with the primers: PaMurE‐F0 and PaMurE‐R0.

### Phylogenetic analysis

Phylogenetic analyses of Mur enzymes except MurJ from all species were reported previously (Sato & Takano, 2017). All predicted amino acid sequences of the isolated *Mur* genes except *MurJ* from *P. abies* in this study were monophyletic with Mur proteins from other plant species when they were combined with the data of Sato and Takano (2017). Therefore, using the maximum likelihood method and JTT matrix‐based model in MEGA11 (Jones et al., 1992; Stecher et al., 2020; Tamura et al., 2021), we generated phylogenetic trees with the amino acid sequences of selected plant species and the bacteria *Anabaena* sp. PCC7120 and *E. coli* (Figure S2). A phylogenetic tree for MurJ was made by the same method with the selected plant species and the two bacteria.

The amino acid sequences from plant species were acquired from the database Phytozome (Goodstein et al., 2012; https://phytozome‐next.jgi.doe.gov). Using BlastP with *P. abies* proteins, Mur proteins were identified from the plant species as follows: mosses *P. patens* and *Sphagnum fallax*, liverwort *M. polymorpha*, lycophyte *S. moellendorffii*, gymnosperm *T. plicata*, and angiosperms *A. thaliana*, *Brassica rapa, Theobroma cacao*, *Gossypium raimondii*, *Prunus persica*, *Malus domestica*, *Solanum lycopersicum*, and *Mimulus guttatus*. Gymnosperm Mur proteins found in the Genbank/DDBJ/EMBL databases by BlastP with the *P. abies* Mur protein were included. Mur proteins of cyanobacterium *Anabaena* sp. PCC7120 (Nostoc sp. PCC7120) and *E. coli* were also used. Amino acid sequences that were shorter than other proteins or that showed many amino acid substitutions were manually removed for phylogenetic analysis. If a longer putative full‐length protein was found in the Genbank/DDBJ/EMBL databases, we used it instead of the shorter sequence in Phytozome.

### Subcellular localization of the Mur proteins

Computational predictions of subcellular protein localization were performed using DeepLoc 2.0 (Thumuluri et al., 2022) software. As the PaMurE protein was predicted to be in the stroma, to determine the subcellular localization of the PaMurE protein, a plasmid expressing a GFP protein fused to the putative transit peptide of PaMurE driven by the CaMV 35S promoter was constructed and transformed into protoplasts of *A. thaliana*, as follows. The N‐terminal region of 333 bp containing the putative transit peptide was amplified with PaMurE‐specific primers: TP‐F and TP‐R, both containing an *Nco*I restriction site. Amplified DNA was digested with *Nco*I and inserted into the *Nco*I‐digested sGFP(S65T) vector (Chiu et al., 1996) to produce pUC18‐35S‐PaMurE‐tp‐sGFP. Transformation of protoplasts of *A. thaliana* was carried out as described by Yoo et al. (2007). GFP fluorescence was observed with a fluorescence microscope (Eclipse 90i; Nikon, Tokyo, Japan).

To determine the subcellular localization of PaPBP, we used the full‐length sequence because it codes the transmembrane region in addition to the plastid‐targeting sequence. A DNA fragment comprising the full‐length coding sequence of *PaPbp* was amplified from the cDNA clone of *PaPbp* with the appropriate primer set for In‐Fusion cloning (Table S1). Linearized sGFP(S65T) vector was prepared by PCR amplification with the sGFP/F0‐Met primer and GFPvec 35S (end) rev primer. DNA for *PaPBP* was cloned between the CaMV 35S promoter and sGFP gene of the sGFP(S65T) vector to express the PaPBP‐GFP fusion protein by using In‐Fusion HD Enzyme Premix (Takara Bio). Using the constructed plasmids, the moss *P. patens* was transformed with PEG as described previously (Machida et al., 2006). Bright field and epifluorescence cell images were recorded with a CCD camera (Zeiss AxioCam) under a microscope (Zeiss Axioskop 2 plus).

### Complementation of Arabidopsis *atmurE‐1* plants with the *PaMurE* gene

The primers PaMurE‐F and PaMurE‐R, both containing a 20 bp overlapping region with the pBI101 vector were designed to amplify the *PaMurE* cDNA. The amplified DNA was ligated to *Bam*HI‐digested pBI101‐35‐Gus‐Hm vector (Lin et al., 2008) by homologous recombination to generate the plasmid pBI101‐PaMurE. The pBI101‐PaMurE was introduced into *Agrobacterium tumefaciens* strain LBA4404 by the freeze–thaw method, and transformation of Arabidopsis plants was performed by a simplified in‐plant infiltration method without application of a vacuum (Clough & Bent, 1998). For the complementation assay, the T‐DNA tagged line *atmurE‐1* was used (Garcia et al., 2008). Because homozygous T‐DNA‐tagged mutants for *AtMurE* showed an albino phenotype, heterozygous plants were used for transformation. For genotyping, genomic PCR experiments were performed with primers: Lba1 and MurE‐LP for detection of the tagged *AtMurE* gene or MurE‐LP and MurE‐RP for the WT *AtMurE* gene. Transgenic lines were obtained by screening on 1/2 MS medium with kanamycin (40 mg L^−1^) and confirmed by genomic PCR with primers PaMurE‐F0 and PaMurE‐R0. Complementation of the albino phenotype of *atmurE‐1* was tested in plants homozygous for T‐DNA‐tagged *AtMurE*. Expression of *PaMurE* and *AtMurE* in transgenic Arabidopsis was investigated by RT–PCR with primer sets: PaMurE‐F0 and PaMurE‐R0 for *PaMurE* and AtMurE‐SP‐F and AtMurE‐SP‐R for AtMurE. The Arabidopsis *actin2* gene was used as an internal control.

The chlorophyll contents of the fifth leaf from 40‐day‐old WT and transgenic Arabidopsis were measured with a SPAD 502 Chlorophyll Meter (Konica Minolta, Inc, Osaka, Japan). Three plants were measured for the WT and each transgenic line, and the experiments were repeated three times. By using SPSS software, significant differences were assessed by one‐way analysis of variance (anova) followed by a *post‐hoc* Tukey's honestly significant difference test (*P* < 0.05).

### Complementation of a moss *PpMurE* knockout line with the *PaMurE* gene

The coding sequence of *PaMurE* was amplified from the cDNA clone by PCR with PaMurE‐F0 and PaMurE‐R0 primers, subjected to blunting and phosphorylation, and inserted into a *Sma*I site between the rice actin promoter and terminator of the pea *rbcS* gene of pTFH22.4 vector (Fujita et al., 2004) including the GFP gene, which is driven by the CaMV 35S promoter to generate a pTFH22.4‐PaMurE plasmid. The plasmid was introduced into *P. patens MurE* knockout cells with a macrochloroplast phenotype. At 5 days after transformation, moss cells with GFP fluorescence were observed by microscopy. The significance of differences was assessed using a non‐parametric Kruskal–Wallis test with IBM SPSS software, and differences between means were considered to be significant at a probability (*P*) value of <0.01.

### Construction of *E. coli* expression vectors

*His_AnMurE_*pPROEX (46T) and *His_PpMurE1‐TP_*pPROEX (22B) were constructed as described (Dowson et al., 2022). To construct *PpMurE‐TP_Avi_His_pET28* (1.1T) and *LgMurE‐SATD_Avi_His*_pET28 (7.3T), the coding sequences of *PpMurE‐TP* in pPROEx (22B) and *LgMurE‐SATD* in LgMurE_pTFH22.4 (Lin et al., 2017) were PCR amplified from Leu63 and Thr254, respectively, using Q5 polymerase and Gibson cloned into *Avi_His_*pET28 (constructed from a synthetic DNA for *TEV_Avi_3C_His* (Table S1) previously Gibson cloned into pET28b). *His_LgMurE‐TP_pQE30* (G) and *His_PaMurE‐SATD_pQE30* (H) were constructed by PCR amplification of the coding sequences, from Gln48 and Thr249, respectively, and the products were digested with *Bam*HI and *Pst*I and ligated into *Bam*HI*‐Pst*I restricted pQE30 vector. Sequences correspond to the Genbank accessions: *P. abies* BER90440.1 and *L. gmelinii* BAX09277.1. *Pseudomonas aeruginosa* MurE and *Streptococcus pneumoniae* MurE were cloned into pET21, as described (Blewett et al., 2004; El Zoeiby et al., 2001). The pOPIN‐based *S. frugiperda* expression vectors were also designed for expression in *E. coli* and their construction is detailed below.

### Construction of *S. frugiperda* expression vectors

Initially, *His_PaMurE‐TP_pQE30* (D) and *His_LgMurE‐SATD_pQE30* (E) were constructed by PCR amplification of the coding sequences, from Ser36 and Thr253, respectively, and the products were digested with *Bam*HI and *Pst*I and ligated into *Bam*HI*‐Pst*I restricted pQE30 vector. *PaMurE‐TP* initiates at the predicted transit peptide cleavage site Ser36 and LgMurE‐SATD initiates at a residue that aligns with the cognate start for bacterial MurE homologs. For *His_Avi_PaMurE‐TP_*pOPINF (B1), first a 134 bp *His_3C_Avi_TEV* sequence was Q5 HiFi DNA polymerase (PCR) amplified (New England Biolabs, Ipswich, MA, USA) from a synthetic fragment (Table S1) and Gibson cloned into PCR amplified *PaMurE‐TP_*pQE30 (D), creating *His_Avi_PaMurE‐TP_*pQE30 (16.7). Likewise, for *His_Avi_LgMurE‐SATD_pOPINF* (B2) an Avi tag was introduced into *His_LgMurE‐SATD_pQE30* (E) to create the intermediate vector *His_Avi_LgMurE‐SATD_pQE30* (19.1). Then the *His_Avi_PaMurE‐TP* and *His_Avi_LgMurE‐SATD* coding sequences were PCR amplified by Phusion Flash polymerase (ThermoFisher Scientific, Waltham, MA, USA) and cloned using InFusion (Takara Bio) into pOPINF (Berrow et al., 2007; kindly supplied by Ray Owens, Rosalind Franklin Institute, UK). All pOPIN vectors were amplified using KOD Hot Start DNA polymerase (Sigma‐Aldrich, Merck KGaA, Darmstadt, Germany). For the shorter coding sequence with homology to cognate bacterial MurE, *PaMurE‐SATD* (from Thr249 in *PaMurE*) was PCR amplified and InFusion cloned into *His_Avi_LgMurE‐SATD_*pOPINF (B2) to substitute the two coding sequences and create *His_Avi_PaMurE‐SATD_*pOPINF (J6).

For *PaMurE‐SATD_Avi_His_*pOPINE (E7), *PaMurE‐SATD* was PCR amplified, again from Thr249 in *PaMurE‐SATD_*pQE30 (D) and cloned by two‐fragment InFusion cloning with a carboxyterminal *TEV_Avi_3C_His* fragment (Table S1), derived from an Avi_His_pET28 construction, into pOPINE. For *PaMurE‐TP_Avi_His_*pOPINE (E6) *PaMurE‐TP* from Ala 67 in *PaMurE‐TP_*pQE30 (D) was substituted for *PaMurE‐SATD* in *PaMurE‐SATD_Avi_His_*pOPINE (E7) by InFusion cloning. For *PpMurE‐TP_Avi_His_*pOPINE (D7) the PpMurE‐TP coding sequence in PpMurE‐TP_Avi_His_pET28 (1.1T) was PCR amplified and InFusion cloned into pOPINE. Coding sequences were confirmed by Sanger sequencing (Eurofins Genomics, Ebersberg, Germany).

### Preparation of baculovirus‐infected *S. frugiperda* cells

Baculovirus bacmid containing mCherry (Kolesnikova et al., 2022) was prepared using the Nucleobond BAC 100 Kit (Takara Bio) and linearized by *Bsu*36I digestion. Transfection mixes comprising 250 ng heat‐treated (70°C, 15 min), linearized bacmid and 100–500 ng pOPIN‐based *MurE* expression vector (and a pOPIN‐*GFP* control) in 50 μl ExpiSf CD Medium (ThermoFisher Scientific) with 1.5 μl Fugene‐HD (Promega, Madison, WI, USA), were incubated for 30 min at room temperature. *Spodoptera frugiperda* ExpiSf9 cells at 5–7 × 10^5^ cells ml^−1^ were dispensed as 500 μl aliquots into a 24‐well plate and left to adhere at room temperature for 1 h before being transfected with 50 μl transfection mix. Plates were incubated static for 6 days at 27°C. The cells were pelleted at 6000× ***g*** and the viral supernatant (P0) amplified by one or two further rounds of infection of ExpiSf9 cells (1:100 viral supernatant:cells) and growth at 27°C, 220 rpm, for 3 days, to create P1 and P2 viral supernatants, respectively.

### MurE protein preparation from *S. frugiperda* cells

Pelleted *S. frugiperda* cells from 50 ml of P2 infected growth were lysed using a 3 ml Dounce homogenizer into a final volume of 7 ml Lysis buffer: 50 mM MOPS (3‐(N‐morpholino)propanesulfonic acid) pH 7.6, 500 mM NaCl, 2 mM MgCl_2_, 0.2% Tween 20 containing 10 mM imidazole, 0.7% (v/v) protease inhibitor cocktail (P8849, Sigma‐Aldrich) and 90 units ml^−1^ Benzonase nuclease (Merck, Merck KGaA, Darmstadt, Germany). Insoluble material was pelleted at 50 000× ***g***, at 4°C for 40 min and the supernatant was withdrawn and mixed with 200 μl Ni‐NTA HisPur beads (ThermoFisher Scientific) on a roller mixer for 1–3 h, at 4°C. The Ni‐NTA resin was transferred to a 10 ml column with a sintered glass base and washed with 2 × 2 ml Lysis buffer containing 10 mM imidazole and 2 × 1 ml Lysis buffer containing 30 mM imidazole. His‐tagged proteins were eluted with 350–700 μl of Lysis buffer containing 300 mM imidazole. Total yield for better expressed proteins was 2–4 mg.

### MurE protein preparation from *E. coli*

*His_AnMurE*_pPROEX (46T), *His_PpMurE1‐TP*_pPROEX (22B) were purified by Ni‐NTA affinity and S200 size exclusion chromatography, as described (Dowson et al., 2022). *PpMurE‐TP_Avi_His*_pET28a (1.1T) and *LgMurE‐SATD_Avi_His_pET28* (7.3T) were expressed in Tuner cells ([DE3] Novagen, Sigma‐Aldrich, Merck KGaA, Darmstadt, Germany), with the chaperone plasmid pG‐KJE8 (Takara Bio), and purified in the same way, except LgMurE was not subjected to size exclusion chromatography due to the low yield. The pQE30‐based vectors were expressed in *E. coli* BL21(DE3):pLysS transformed with the plasmid pREP4groESL in L‐Broth plus 1% (v/v) glucose, 50 mg ml^−1^ kanamycin and 100 mg ml^−1^ ampicillin at 37°C to an *A*_600_ of 0.8 when recombinant protein expression was induced with 0.1 mM IPTG and a repeat addition of antibiotics. Bacteria were then grown overnight at 11°C, harvested by centrifugation at 5600× ***g***, 15 min at 4°C and resuspended in Buffer A: 50 mM HEPES–NaOH, 0.5 m NaCl, 0.5 mM MgCl_2_, 5% (v/v) glycerol (pH 8.0), EDTA‐free protease inhibitor tablets, as recommended by the supplier (Pierce, ThermoFisher Scientific, Waltham, MA, USA), 1 mM ß‐mercaptoethanol, 0.5 mg ml^−1^ lysozyme and 0.03 mg ml^−1^ DNase at a ratio of 1:10 (w/v), with gentle mixing for 30 min at 4°C. Lysis was by passing the resuspended cells through a cell disruptor at 15 000–20 000 psi, three times. Insoluble material was pelleted at 20 000× ***g*** for 30 min at 4°C and the supernatant was loaded directly onto a 5 ml His Trap HP (GE Healthcare) and washed with 25 ml Buffer A at 2 ml min^−1^, 4°C. Bound material was eluted with a 100 ml linear gradient to 100% Buffer B: 50 mM HEPES–NaOH, 0.5 m NaCl, 5% (v/v) glycerol, and 0.5 m imidazole (pH 7.5) at 1 ml min^−1^. Selected peak fractions were pooled and concentrated in 30 kDa MWCO Vivaspin concentrators (Cytiva, Merck KGaA, Darmstadt, Germany), at 2800× ***g*** at 4°C. Protein was further purified by size exclusion chromatography on Superdex G200 Increase (Cytiva, Merck KGaA), pre‐equilibrated and eluted with 50 mM TRIS–HCl, 150 mM NaCl (pH 7.5). Pooled peak fractions were dialyzed against DB2: 30 mM HEPES–NaOH, 1 mM MgCl_2_, 50 mM NaCl, 50% (v/v) glycerol with 0.2 mM PMSF, 1 mM leupeptin, 1 mM pepstatin, 3 mM dithiothreitol (pH 7.6) overnight at 4°C, before storage at −20°C and −80°C.

Bacterial expression of pOPIN‐based *PaMurE* was in BL21*Rosetta (Novagen, Sigma‐Aldrich). Cells were grown to log phase in L‐Broth containing 1.5% glucose, 100 mg ml^−1^ ampicillin and 35 mg ml^−1^ chloramphenicol at 37°C to an *A*_600_ of 0.6 when expression was induced with 0.1 mM IPTG with a repeat addition of antibiotics and the cultures were grown overnight at 16°C. Cells were pelleted and lysed as above by cell disruption into Buffer A, but with MOPS in place of HEPES, 10 mM imidazole, 1 mM Tris(2‐carboxyethyl)phosphine hydrochloride, and 1 mM methionine. Proteins were purified on 5 ml His traps using MOPS‐based buffers, but as above, and peak fractions were dialysed at 4°C overnight into 25 mM MOPS, 200 mM NaCl, 1 mM MgCl_2_, 0.5 mM methionine, 50% (v/v) glycerol (pH 7.6) with 0.2 mM PMSF, 1 mM leupeptin, and 1 mM pepstatin.

Purity of the dialysed MurE proteins was established by sodium dodecyl sulfate–polyacrylamide gel electrophoresis (Figure S3) and proteins to be assayed by the Amplex Red assay that had been previously dialysed in the presence of dithiothreitol were dialysed a second time in the absence of any reducing agent into 25 mM MOPS, 200 mM NaCl, 1 mM MgCl_2_, 50% (v/v) glycerol (pH 7.6). Removal of the His_Avi tag from PpMurE‐TP_Avi_His (1.1T; Figure S3b) was as described (Dowson et al., 2022).

### Amplex red enzymatic assays of PaMurE and LgMurE proteins

Assays of MurE‐dependent generation of phosphate were performed at 30°C, pH 7.6, in a 10 μl volume/384‐well format, containing 50 mM MOPS, 10 mM MgCl_2_, 0.5 mM inosine, 2.5 mM.min^−1^ *Arthrobacter* sp. xanthine oxidase, 20 mM.min^−1^ horse radish peroxidase, 50 μM Amplex Red, 2.64 mM.min^−1^ *E. coli* purine nucleoside phosphorylase, 20.1 μM ATP, 0.1 mM UDP‐MurNAc‐l‐Ala‐γ‐d‐Glu, 0.29 mg ml^−1^ *P. aeruginosa* MurE or 2.5–67.6 mg ml^−1^ of the other MurE proteins and, where added, 8 mM diaminopimelic acid (d, l‐2,6‐DAP, racemic mixture, meso‐DAP, Sigma‐Aldrich (92591)), or 2.8–6.6 mM amino acid substrate for the substrate assays. MurE was assayed in three wells where the reaction was initiated by the addition of an equal volume of 16 mM DAP and in three wells where the reaction (control) was initiated by water. The fluorescent product of the reaction cascade (resorufin—derived from Amplex Red) was continuously monitored from above the well at excitation and emission wavelengths of 545 nm and 585 nm, respectively, in a Varioskan Lux plate reader (ThermoFisher Scientific). Control reactions were carried out in the absence of added MurE which confirmed detectable activity was dependent on the addition of MurE. Assay responses were linearly dependent upon phosphate in the range 0–20 μM, allowing calculation of MurE rates from the relationship fluorescence/pmol phosphate = 2.274 fluorescence units/pmol phosphate. For substrate assays where the amino acid substrate was close to rate‐limiting (30 μM) the concentrations were as above with 100 μM ATP.

A similar spectrophotometric cuvette‐based derivative of the above MurE assay was also performed in triplicate in a final volume of 200 ml at 37°C, pH 7.6, containing 50 mM HEPES, 10 mM MgCl_2_, 0.5 mM inosine, 2.5 mM.min^−1^ *Arthrobacter* sp. xanthine oxidase, 20 mM.min^−1^ horse radish peroxidase, 50 μM Amplex Red, 2.64 mM.min^−1^ *E. coli* purine nucleoside phosphorylase, 0.1 mM ATP, 0.1 mM UDP‐MurNAc‐l‐Ala‐γ‐d‐Glu, and 5.65–23.8 mg ml^−1^ plant MurE. Initial rate data were collected in a Cary 100 UV/Vis double beam spectrophotometer at 555 nm for 2 min and MurE activity was initiated by addition of d, l‐2,6‐DAP to 0.5 mM where the difference in rates was proportional to MurE activity. The assay was calibrated with respect to phosphate between 0 and 50 μM phosphate yielding a molar absorbance relative to phosphate of 46 840 m phosphate^−1^.cm^−1^, allowing calculation of MurE rates. Alternative substrates, from Sigma‐Aldrich, included l‐Lysine (L5501), d‐Lysine (L5876), d, l‐Lanthionine (L8543), l‐Cystathionine (C7505), l‐2,4‐Diaminobutyric acid (32 830M), d, l‐Hydroxylysine‐HCl (H0377), l‐Ornithine (O6503), and l‐Homoserine (H6515). The d, d and l, l‐DAP stereoisomers were further purified by TLC. For all assays, rates were related to protein added to the assay where protein concentrations were determined according to Bradford (1976).

### NADH enzymatic assays of PaMurE and LgMurE proteins

Assays of MurE‐dependent generation of ADP were performed at 30°C in a 50 μl volume/384 well format. The loss of absorbance of NADH at *A*_340_ in the reaction cascade (that is coupled to ADP release by the enzymes pyruvate kinase and lactate dehydrogenase) was continuously monitored from above in a Clariostar plate reader (BMG Labtech, Ortenberg, Germany).

Assay concentrations were those reported by Dowson et al. (2022).

### MS/MS analyses of purified proteins

The methods for NanoLC‐ESI‐MS/MS analysis of the gel‐extracted trypsin digests of cyanobacterial and plant MurE ligases, expressed in either baculovirus‐infected Sf9 insect cells or *E. coli* are described and post‐translational modifications are interpreted in Method S1, with results being presented in Figure S7. Raw data were searched using FragPipe version 18.0 (https://fragpipe.nesvilab.org/) against *S. frugiperda, E. coli* databases (https://www.uniprot.org/), and the protein sequences provided. For the database search, peptides were generated from a tryptic digest with up to two missed cleavages and carbamidomethylation of cysteines as fixed modifications. As variable modifications, methionine oxidation, phosphorylation of serine, threonine and tyrosine, acetylation of the protein N‐terminus, ubiquitination and methylation of lysine were added. Scaffold software (https://www.proteomesoftware.com/products/scaffold‐5) was used for data analysis and visualization of results.

### Complementation of a moss *PpPBP* knockout line with the *PaPBP* gene

The coding sequence of *PaPbp* was amplified from the cDNA clone by PCR with PaPbp/Met‐F and PaPbp/stop‐R primers, subjected to blunting and phosphorylation, and inserted into a *Sma*I site between the rice actin promoter and terminator of the pea *rbcS* gene of pTFH22.4 vector to generate a pTFH22.4‐*PaPbp* plasmid which was introduced into *P. patens* ∆*PpPbp* cells having a macrochloroplast phenotype. At 5 days after transformation, GFP fluorescence of moss cells was assessed by microscopy.

### Generation of stable complementation lines of moss ∆ *PpPbp* with *PaPbp* gene

The coding sequence of *PaPbp* with the heterologous promoter and terminator was amplified from the pTFH22.4‐*PaPbp* plasmid by PCR with Act1p‐PIG1b and Trbcs‐m35sp primers. From the pPGX8 vector (Kubo et al., 2013), the backbone of the vector including the hygromycin resistance gene and two *P. patens* genomic regions (PIG1L and 1R) for insertion into the genome by homologous recombination was also amplified by PCR with PIG1b (PGX8) and m35sp (PGX8) primers, and both PCR products were assembled in the In‐Fusion reaction to create a pPGX8‐*PaPbp* plasmid. The constructed plasmid was linearized and used to transform moss cells. Genomic and RT‐PCR were used to prove the insertion of the *PaPbp* gene at the target site and expression of *PaPbp* in ∆*PpPbp*, respectively (Figure S8). Ampicillin was used at a final concentration of 100 μM for 2 weeks. The numbers of chloroplasts in chloronemal subapical cells were determined under a light microscope. Chloroplast numbers were compared using the non‐parametric Kruskal–Wallis test.

## AUTHOR CONTRIBUTIONS

HT and AJD planned, designed and performed the experiments and authored the manuscript. AJL designed the Amplex Red assay, contributed to the enzymatic assays and manuscript correction. LD‐S assisted with insect cell transformations and advice. CGD assisted with manuscript edits. All authors revised and approved the manuscript. EY, JZ, JW, and XL isolated PaMurE and conducted the experiments using Arabidopsis. YS, IK, and KT isolated other *Mur* genes from *P. abies* and carried out moss experiments.

## CONFLICT OF INTEREST

The authors declare that there is no conflict of interest.

## Supporting information


**Data S1.** Supplementary Method S1.

**Table S1.** Primers used in this study.

**Table S2.** *Mur* genes in the gymnosperm genomes.

**Figure S1.** Phylogenetic trees for Mur proteins in plants.
**Figure S2.** Mur gene amino acid alignment.
**Figure S3.** PAGE (10%) of MurE proteins used for activity studies.
**Figure S4.** Enzymatic assays of UDP‐N‐acetylmuramoyl‐l‐alanyl‐d‐glutamate‐‐2,6‐diaminopimelate ligase (MurE) proteins.
**Figure S5.** Time courses of resorufin fluorescence from Amplex Red, consequent on Pi release in reactions catalyzed by plant MurE ligases.
**Figure S6.** Multiple sequence alignment of bacterial and plant MurE ligases and homologs with reported ligand binding residues identified.
**Figure S7.** LC–MS data for post‐translational modifications of MurE ligases expressed in baculovirus‐infected *S. frugiperda* (Sf9) and *E. coli*.
**Figure S8.** Generation of stable transformants expressing *PaPBP* gene in ∆PpPbp.

## Data Availability

The data that support the findings of this study are available in the supplementary material of this article. Accession numbers: LC777194–LC777204 for PaMurA–PaPbp (Figure 1).
