# Supplementary material for: Transcriptomic and enzymological evidence for plastid peptidoglycan synthesis in the gymnosperm Picea abies
Source: Plant J. 2025 Dec 6;124(5):e70588. doi: 10.1111/tpj.70588 (PMC12681378; doi:10.1111/tpj.70588)
Supplement: Supplementary file 1 — Data S1. Supplementary Method S1. [file TPJ-124-0-s001.docx]

**Supplementary Method S1**

**NanoLC-ESI-MS/MS Analysis**

Proteins bands were excised from 10% (w/v) PAGE gels, diced and destained with repeat 20 min washes of 50 mM ammonium bicarbonate (ABC) in 50% ethanol at 20ºC, 650rpm shaking, until clear, and then dehydrated with 100% ethanol, 5 min, at 20ºC, 650rpm. Proteins were reduced and alkylated in 10 mM Tris-(2-carboxethyl) phosphine hydrochloride with 40 mM 2-chloroacetamide, 5 min, 70ºC and the washes and dehydration were repeated. Dried gel pieces were incubated in 2.5 ng/µL sequencing grade modified trypsin (V5111 Promega) in 50 mM ABC, overnight at 20ºC. The liquid was retained and the peptides in the gel pieces extracted three times into 5% formic acid in 25% acetontitrile, 7 min, at 20ºC and the liquids combined. Peptides were dried down in a Speed-Vacuum at 60ºC and resuspended in 2% acetonitrile, 0.5% trifluoric acid, ready for LC-MS.

Reversed phase chromatography was used to separate tryptic peptides prior to mass spectrometric analysis. Two C18 columns were utilised, an Acclaim PepMap µ-precolumn cartridge 300 µm i.d. x 5 mm 5 μm 100 Å (Thermo Fisher Scientific) and a 75 µm x 40 cm 1.5 µm (Bruker PepSep Series Analytical column). The Ultimate 3000 RSLCnano system (Thermo Fisher Scientific) was used with mobile phase buffer A composed of 0.1% formic acid in water and mobile phase B 0.1 % formic acid in acetonitrile. Samples were loaded on the µ-precolumn equilibrated in 2% aqueous acetonitrile containing 0.1% Trifluoroacetic and peptides were eluted onto the analytical column at 300 nL min^-1^ by increasing the mobile phase B concentration from 4% B to 25% over 36 min, then to 35% B over 10 min, and to 90% B over 3 min, followed by a 10 min re-equilibration at 4% B.

Ultimate 3000 RSLCnano was coupled online to a hybrid timsTOF Pro (Bruker Daltonics, Germany) via a CaptiveSpray nano-electrospray ion source (Meier et al.,2018). The timsTOF Pro was operated in Data-Dependent Parallel Accumulation-Serial Fragmentation (PASEF) mode. Peptides were separated by ion mobility depending on their collisional cross sections and charge states. The method settings were as follows: mass range 100 to 1700 m/z, ion mobility range 1/K0 Start 0.6 Vs/cm^2^ End 1.6 Vs/cm^2^, Ramp rate 9.42 Hz and Duty cycle 100%.

Comparison between proteins indicated that the insect cell-derived ligases were generally more oxidized, probably a consequence of the small volume protein preparation protocol, but oxidation of methionine residues did not appear to be proportional to lower enzymatic activity. More noticeable were the acetylation profiles, with the *E. coli*-derived PaMurE proteins being extensively acetylated, particularly in the carboxy-terminal domain on the lysine preceding the d, l-DAP-specific DNPR motif (Figure S7c and d, red boxed area #4). The same lysine in *E. coli*-expressed LgMurE was more extensively methylated or ubiquitinylated, as were the insect cell-expressed PaMurE proteins (Figure S7, red boxed area #4 in a and b). In fact, acetylation of the insect cell-expressed proteins was almost exclusively on a residue proximal to the amino terminus. The lysine proximal to the DNPR is of particular interest as it is highly conserved in the plant MurE homologs and PyMOL predictions indicate it lies on the surface of the protein, whereby it may be capable of controlling the alignment of the catalytic site DNPR or mediating an interaction with other proteins.

Other differences included phosphorylation, the *E. coli*-expressed gymnosperm proteins being frequently phosphorylated, particularly on a threonine in the **T**EAC/**T**EVC motif (Figure S7c - e, red boxed area #2). Again, this sequence is not conserved in procaryotic MurE and is predicted to be on the surface of the protein and unlikely to be directly involved in catalytic activity.

Carbamylation of a lysine, linked to Mg^2+^ binding in the Mur ligase family, has been positively implicated in activity of *E. coli* MurE ligase (Dementin et al., 2002), and is present in the published crystal structures of both *E. coli* and *Mycobacterium tuberculosum* MurE (PDB: 1E8C and 2WTZ, respectively, Gordon et al., 2001 and Basavannacharya et al., 2010). The equivalent lysine residue in the plant and cyanobacterial proteins (identified by red arrows in red boxed area #2 of Figure S7a and red boxed area #3 in S7c) was weakly carbamylated in *E. coli*-expressed AnMurE (46T) (0.47%) but was not detected as being carbamylated in any of the plant MurE ligases. However, within the same box, the surface facing lysine in the plant consensus **K**TEE was found to be carbamylated. Interestingly, where there were fragments that spanned the conventionally carbamylated lysine reported in *E. coli* MurD (Dementin et al. 2001) and also in the PDB crystal structure depositions for *M. tuberculosis* (2WTZ) and *E. coli* MurE (1E8C), the lysine residue was found to be predominantly methylated in His_AnMurE (46T) (25.9%) and acetylated in His_PpMurE-TP (22B) (36.1%) and PpMurE-TP_Avi_His (1.1T) (71.7%) (Fig S7d, box 3 figures in parenthesis) although the few fragmentations for the gymnosperm proteins were not found to be modified at all. PyMOL alignments would indicate that these methylations and acetylations, as well as any carbamylation of the lysine in the **K**TTT motif in the PaMurE proteins which is very closely proximal to the second Mg^2+^ ion, (blue arrows in Figure S7a and S7c) would have a significant, and potentially negative, impact on retention of the Mg2^+^ ion at the catalytic site.

It was evident that the SATD of the *E. coli*-expressed PaMurE-TP proteins was extensively methylated on a number of lysines and particularlyin the sequences PV**K**DEE and LE**K**EEV(15.8% and 19.6% for His_Avi_PaMurE-TP, and 8.3% and 11.6% for PaMurE-TP_Avi_His, in green boxes of Figure S7c ). The former lysine was almost equally ubiquitinylated. When expressed in Sf9 cells the SATD was also methylated, especially on the lysine in LE**K**EEV (15.5% Figure S7a small grren box). Notably, in *E.coli* most of the other methylations, acetylations and carbamylations, occur, often interchangeably, on residues that would be expected to lie on the surface of protein and especially around the entrance to the active site and therefore may play a complex role in regulating MurE functionality through protein:protein or protein:nucleic acid interactions. However, these data, although indicative of how the proteins may be being regulated in not only the heterologous host but also the original plant cell, do not provide sufficient evidence to account for differences in activity between the PaMurE and LgMurE proteins as evidenced here.

**References**

Basavannacharya, C., Moody, P.R., Munshi, T., Cronin, N., Keep, N.H. and Bhakta, S. (2010) Essential residues for the enzyme activity of ATP-dependent MurE ligase from *Mycobacterium tuberculosis*. *Protein Cell*, 1, 1011-1022.

Dementin, S., Bouhss, A., Auger, G., Parquet, C., Mengin-Lecreulx, D., Dideberg, O. et al. (2001) Evidence of a functional requirement for a carbamoylated lysine residue in MurD, MurE and MurF synthetases as established by chemical rescue experiments. *Eur. J. Biochem.*, 268, 5800–5807.

Gordon, E., Flouret, B., Chantalat, L., van Heijenoort, J., Mengin-Lecreulx, D. and Dideberg, O. (2001) Crystal structure of UDP-N-acetylmuramoyl-l-alanyl-d-glutamate: meso-diaminopimelate ligase from *Escherichia coli*. J*. Biol. Chem.*, 276, 10999-11006.

Meier, F., Brunner, A.D., Koch, S., Koch, H., Lubeck, M., Krause, M. et al. (2018) Online parallel accumulation–serial fragmentation (PASEF) with a novel trapped ion mobility mass spectrometer. *Mol. Cell. Proteom.*, 17, 2534–2545.
