# Supplementary material for: Transcriptomic and enzymological evidence for plastid peptidoglycan synthesis in the gymnosperm Picea abies
Source: Plant J. 2025 Dec 6;124(5):e70588. doi: 10.1111/tpj.70588 (PMC12681378; doi:10.1111/tpj.70588)
Supplement: Supplementary file 4 — Figure S1. Phylogenetic trees for Mur proteins in plants. Figure S2. Mur gene amino acid alignment. Figure S3. PAGE (10%) of MurE proteins used for activity studies. Figure S4. Enzymatic assays of UDP‐N‐acetylmuramoyl‐l‐alanyl‐d‐glutamate‐‐2,6‐diaminopimelate ligase (MurE) proteins. Figure S5. Time courses of resorufin fluorescence from Amplex Red, consequent on Pi release in reactions catalyzed by plant MurE ligases. Figure S6. Multiple sequence alignment of bacterial and plant MurE ligases and homologs with reported ligand binding residues identified. Figure S7. LC–MS data for post‐translational modifications of MurE ligases expressed in baculovirus‐infected S. frugiperda (Sf9) and E. coli. Figure S8. Generation of stable transformants expressing PaPBP gene in ∆PpPbp. [file TPJ-124-0-s003.pdf]

Supporting Figures

Transcriptomic and enzymological evidence for plastid peptidoglycan synthesis in the gymnosperm *Picea abies*.

Yayoi Sugita, Amanda J. Dowson et al.

For correspondence
(e-mail takano@kumamoto-u.ac.jp and A.J.Dowson@warwick.ac.uk)

References

Basavannacharya, C., Moody, P.R., Munshi, T., Cronin, N., Keep, N.H. and Bhakta, S. (2010) Essential residues for the enzyme activity of ATP-dependent MurE ligase from *Mycobacterium tuberculosis*. *Protein Cell*, 1, 1011-1022.

Dementin, S., Bouhss, A., Auger, G., Parquet, C., Mengin-Lecreulx, D., Dideberg, O. et al. (2001) Evidence of a functional requirement for a carbamoylated lysine residue in MurD, MurE and MurF synthetases as established by chemical rescue experiments. *Eur. J. Biochem.*, 268, 5800–5807.

Gordon, E., Flouret, B., Chantalat, L., van Heijenoort, J., Mengin-Lecreulx, D. and Dideberg, O. (2001) Crystal structure of UDP-N-acetylmuramoyl-L-alanyl-D-glutamate: meso-diaminopimelate ligase from *Escherichia coli*. J. *Biol. Chem.*, 276, 10999-11006.

Madeira, F., Park, Y.M., Lee, J., Buso, N., Gur, T., Madhusoodanan, N. et al. (2019) The EMBL-EBI search and sequence analysis tools APIs in 2019. *Nucl. Acids Res.*, 47, W636-W641.

Maitra, A., Munshi, T., Healy, J., Martin, L.T., Vollmer, W., Keep, N.H. and Bhakta, S. (2019) Cell wall peptidoglycan in *Mycobacterium tuberculosis*: An Achilles' heel for the TB-causing pathogen. *FEMS Microbiol. Rev.*, 43, 548-575.

Ruane, K.M., Lloyd, A.J., Fulop, V., Dowson, C.G., Barreteau, H., Boniface, A. et al. (2013) Specificity determinants for lysine incorporation in *Staphylococcus aureus* peptidoglycan as revealed by the structure of a MurE enzyme ternary complex. *J. Biol. Chem.*, 288, 33439-33448.

Smith, C.A. (2006) Structure, function and dynamics in the mur family of bacterial cell wall ligases. *J. Mol. Biol.*, 362, 640-655.

Waterhouse, A.M., Procter, J.B., Martin, D.M.A, Clamp, M. and Barton, G.J. (2009) Jalview Version 2 - A multiple sequence alignment editor and analysis workbench. *Bioinformatics*, 25, 1189-1191.

Figure S1. Phylogenetic trees for Mur proteins in plants
(a) MurA

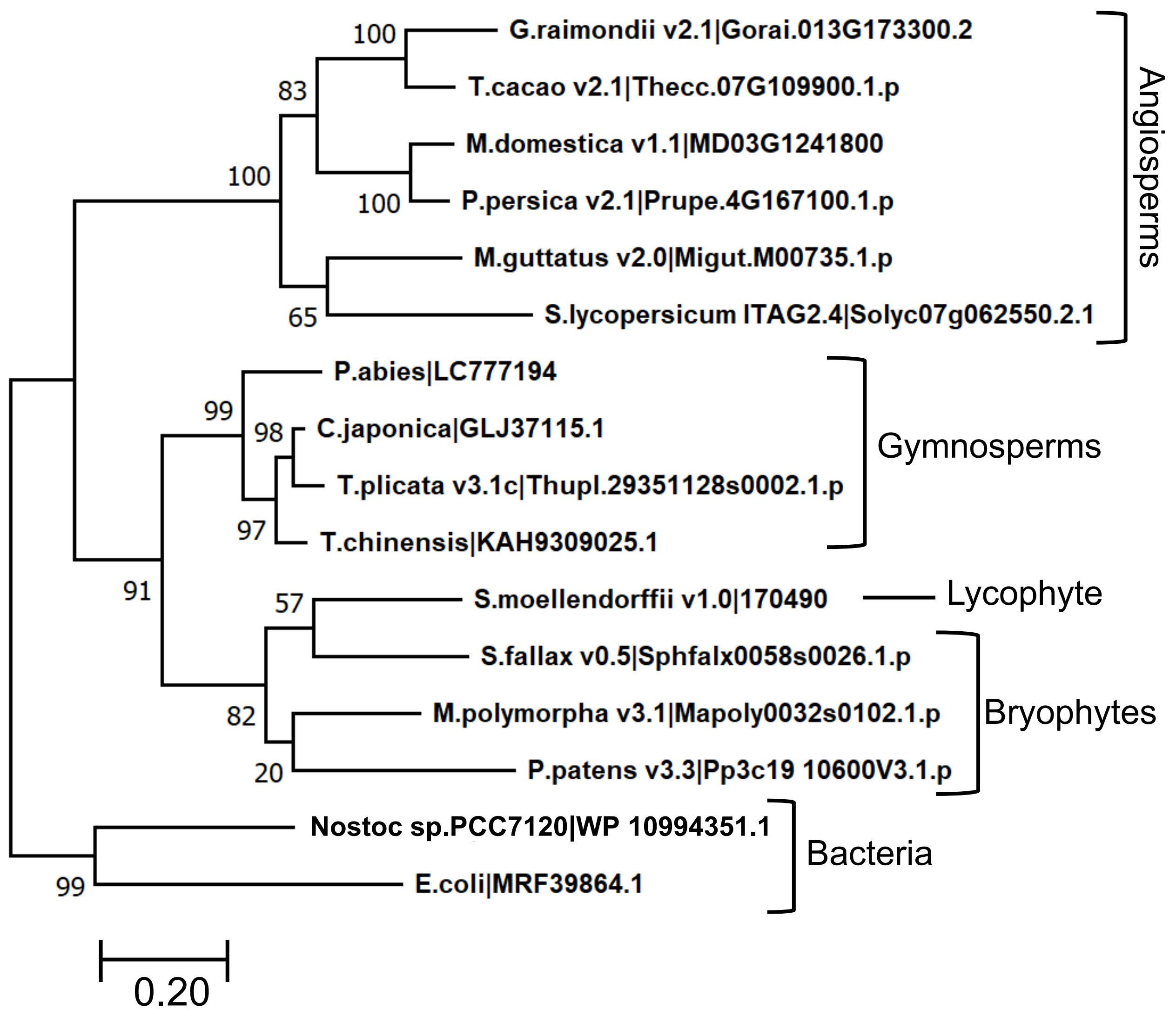

(b) MurB

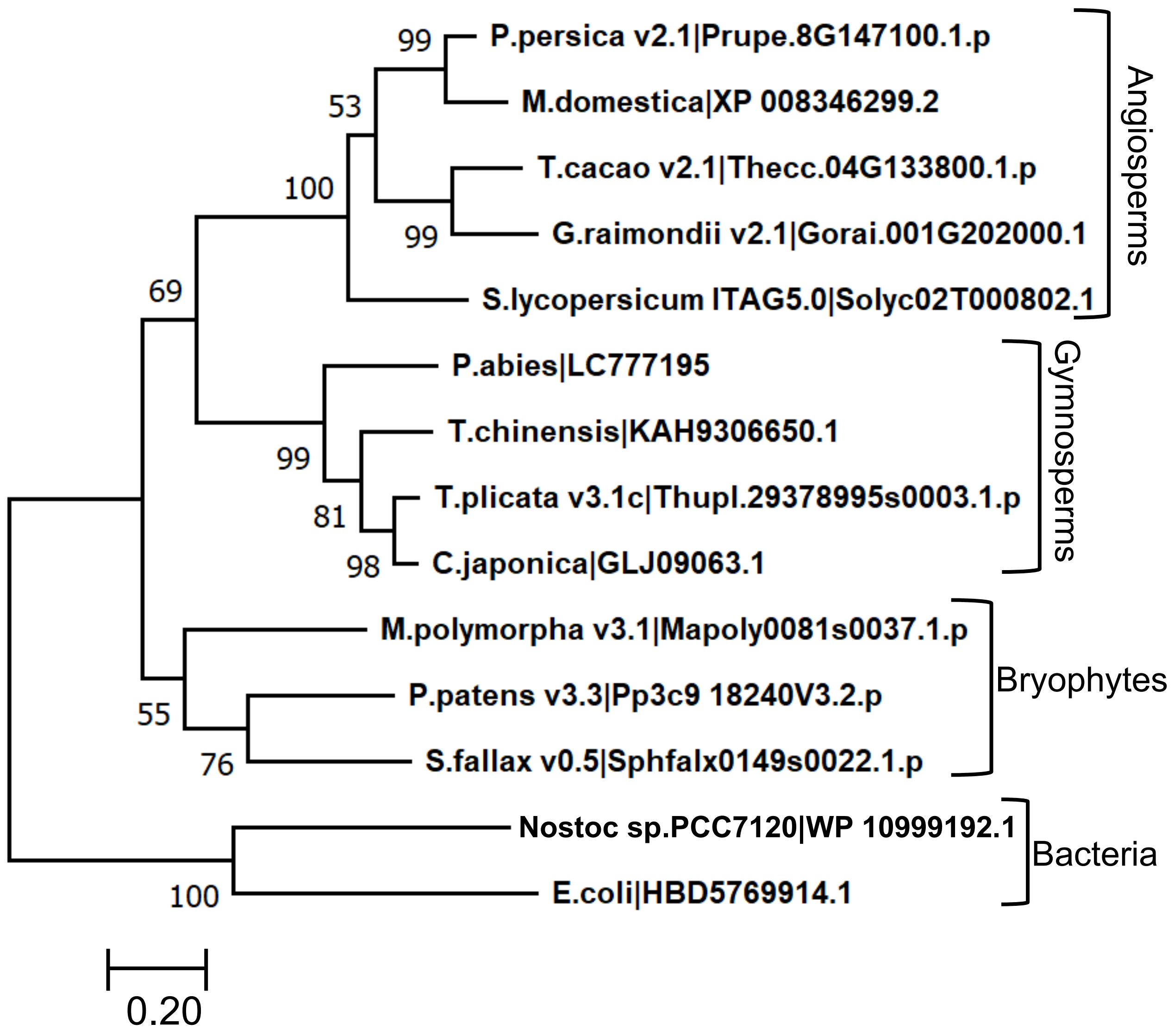

P.persica v2.1|Prupe.8G147100.1.p
M.domestica|XP 008346299.2
T.cacao v2.1|Thecc.04G133800.1.p
G.raimondii v2.1|Gorai.001G202000.1
S.lycopersicum ITAG5.0|Solyc02T000802.1
P.abies|LC777195
T.chinensis|KAH9306650.1
T.plicata v3.1c|Thupl.29378995s0003.1.p
C.japonica|GLJ09063.1
M.polymorpha v3.1|Mapoly0081s0037.1.p
P.patens v3.3|Pp3c9 18240V3.2.p
S.fallax v0.5|Sphfalx0149s0022.1.p
Nostoc sp.PCC7120|WP 10999192.1
E.coli|HBD5769914.1
Angiosperms
Gymnosperms
Bryophytes
Bacteria
99
53
100
99
69
99
81
98
55
76
100
0.20

(c) MurC

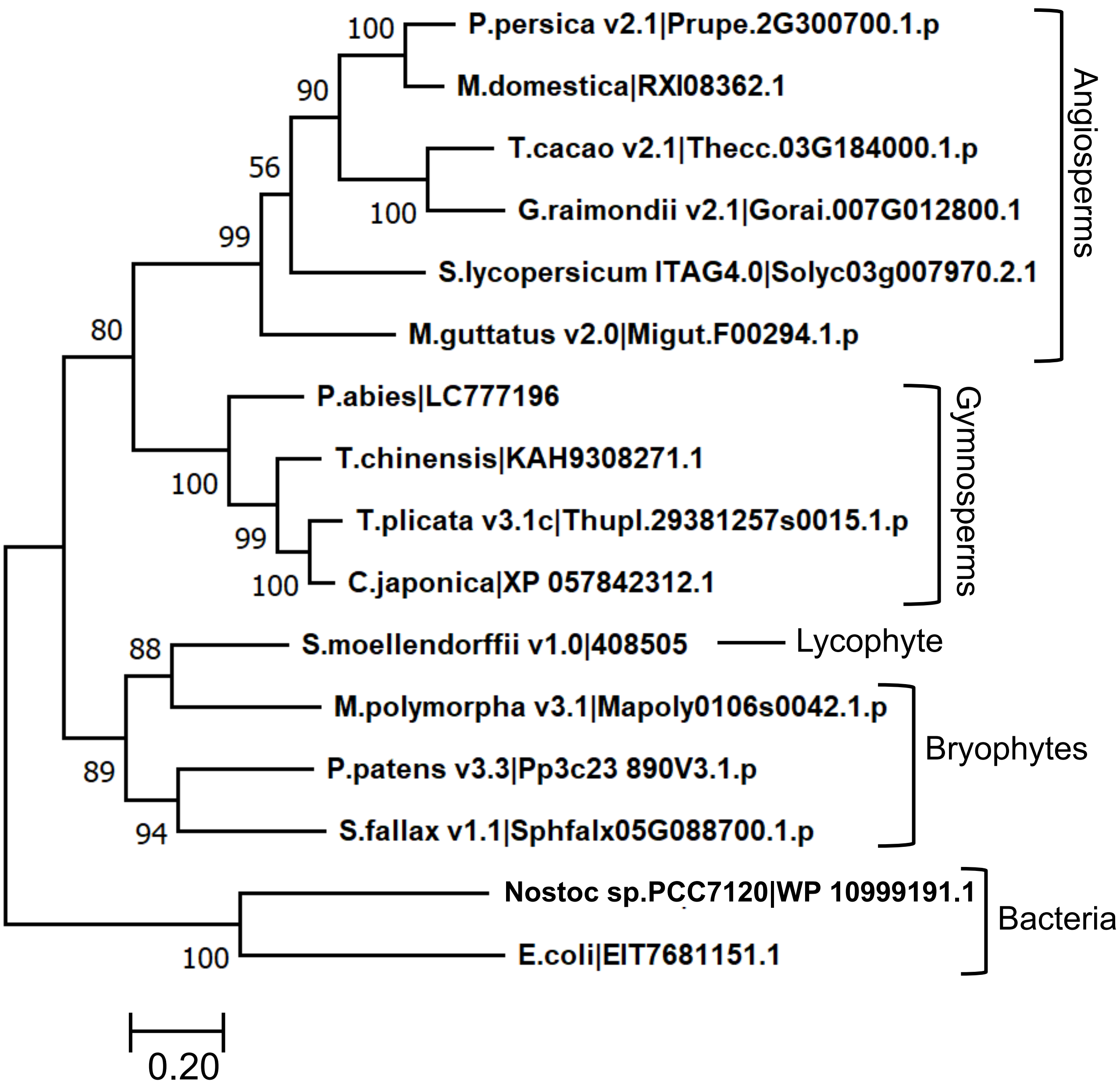

P.persica v2.1|Prupe.2G300700.1.p
M.domestica|RXI08362.1
T.cacao v2.1|Thecc.03G184000.1.p
G.raimondii v2.1|Gorai.007G012800.1
S.lycopersicum ITAG4.0|Solyc03g007970.2.1
M.guttatus v2.0|Migut.F00294.1.p
P.abies|LC777196
T.chinensis|KAH9308271.1
T.plicata v3.1c|Thupl.29381257s0015.1.p
C.japonica|XP 057842312.1
S.moellendorffii v1.0|408505
M.polymorpha v3.1|Mapoly0106s0042.1.p
P.patens v3.3|Pp3c23 890V3.1.p
S.fallax v1.1|Sphfalx05G088700.1.p
Nostoc sp.PCC7120|WP 10999191.1
E.coli|EIT7681151.1
Angiosperms
Gymnosperms
Lycophyte
Bryophytes
Bacteria
100
90
56
99
80
100
100
99
100
88
89
94
100
0.20

(d) MurD

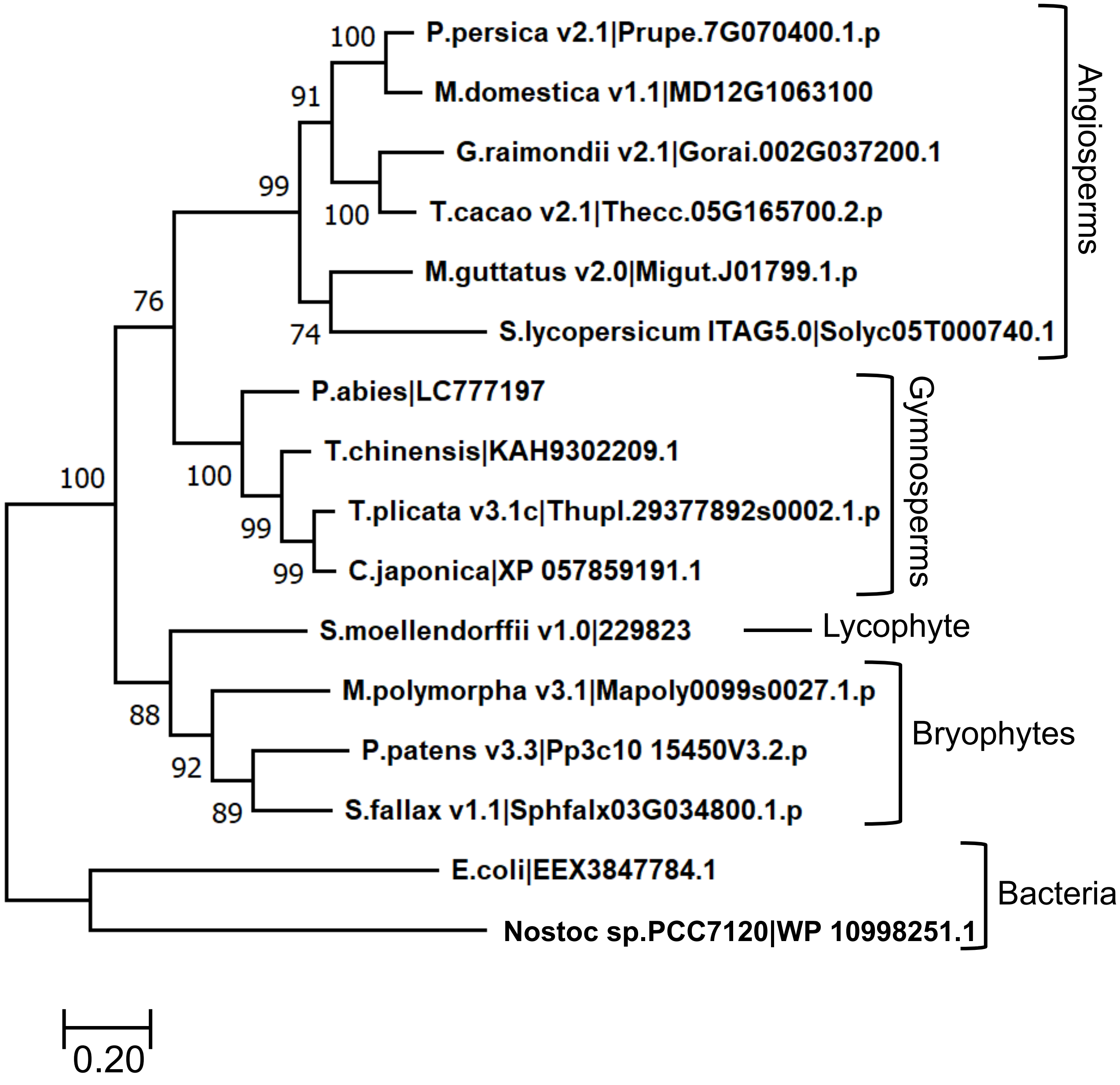

100
P.persica v2.1|Prupe.7G070400.1.p
91
M.domestica v1.1|MD12G1063100
G.raimondii v2.1|Gorai.002G037200.1
99
100
T.cacao v2.1|Thecc.05G165700.2.p
M.guttatus v2.0|Migut.J01799.1.p
76
74
S.lycopersicum ITAG5.0|Solyc05T000740.1
Angiosperms
P.abies|LC777197
T.chinensis|KAH9302209.1
100
100
T.plicata v3.1c|Thupl.29377892s0002.1.p
99
99
C.japonica|XP 057859191.1
Gymnosperms
S.moellendorffii v1.0|229823
Lycophyte
M.polymorpha v3.1|Mapoly0099s0027.1.p
88
P.patens v3.3|Pp3c10 15450V3.2.p
92
89
S.fallax v1.1|Sphfalx03G034800.1.p
Bryophytes
E.coli|EEX3847784.1
Nostoc sp.PCC7120|WP 10998251.1
Bacteria
0.20

(e) MurE

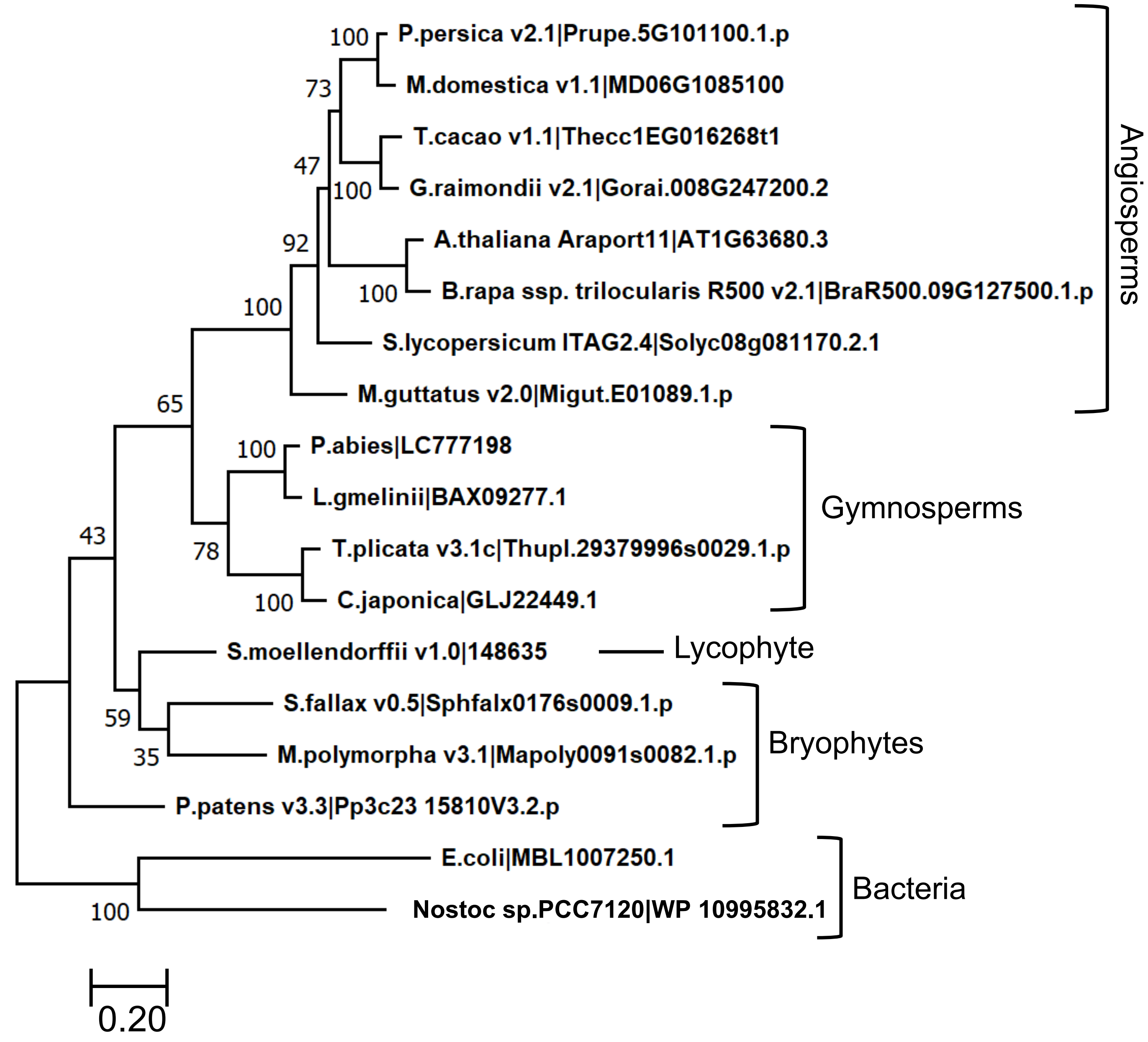

P.persica v2.1|Prupe.5G101100.1.p
M.domestica v1.1|MD06G1085100
T.cacao v1.1|Thecc1EG016268t1
G.raimondii v2.1|Gorai.008G247200.2
A.thaliana Araport11|AT1G63680.3
B.rapa ssp. trilocularis R500 v2.1|BraR500.09G127500.1.p
S.lycopersicum ITAG2.4|Solyc08g081170.2.1
M.guttatus v2.0|Migut.E01089.1.p
P.abies|LC777198
L.gmelinii|BAX09277.1
T.plicata v3.1c|Thupl.29379996s0029.1.p
C.japonica|GLJ22449.1
S.moellendorffii v1.0|148635
S.fallax v0.5|Sphfalx0176s0009.1.p
M.polymorpha v3.1|Mapoly0091s0082.1.p
P.patens v3.3|Pp3c23 15810V3.2.p
E.coli|MBL1007250.1
Nostoc sp.PCC7120|WP 10995832.1
Angiosperms
Gymnosperms
Lycophyte
Bryophytes
Bacteria
100
73
47
100
92
100
100
65
100
78
100
43
59
35
100
0.20

(f) DDL

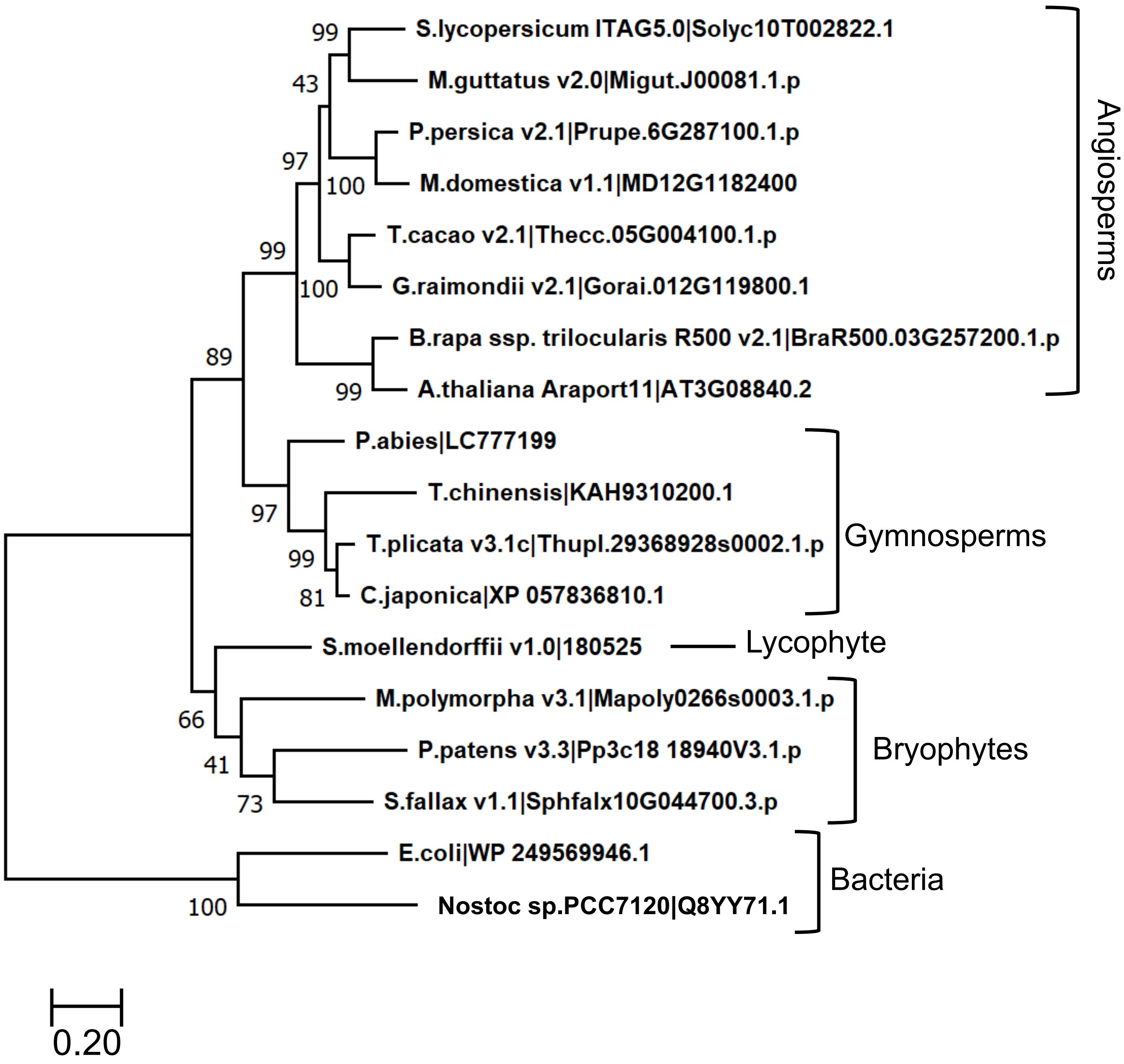

99
S.lycopersicum ITAG5.0|Solyc10T002822.1
43
M.guttatus v2.0|Migut.J00081.1.p
97
P.persica v2.1|Prupe.6G287100.1.p
100
M.domestica v1.1|MD12G1182400
99
T.cacao v2.1|Thecc.05G004100.1.p
100
G.raimondii v2.1|Gorai.012G119800.1
89
B.rapa ssp. trilocularis R500 v2.1|BraR500.03G257200.1.p
99
A.thaliana Araport11|AT3G08840.2
Angiosperms
P.abies|LC777199
97
T.chinensis|KAH9310200.1
99
T.plicata v3.1c|Thupl.29368928s0002.1.p
81
C.japonica|XP 057836810.1
Gymnosperms
S.moellendorffii v1.0|180525
Lycophyte
66
M.polymorpha v3.1|Mapoly0266s0003.1.p
41
P.patens v3.3|Pp3c18 18940V3.1.p
73
S.fallax v1.1|Sphfalx10G044700.3.p
Bryophytes
E.coli|WP 249569946.1
100
Nostoc sp.PCC7120|Q8YY71.1
Bacteria
0.20

(g) MurF

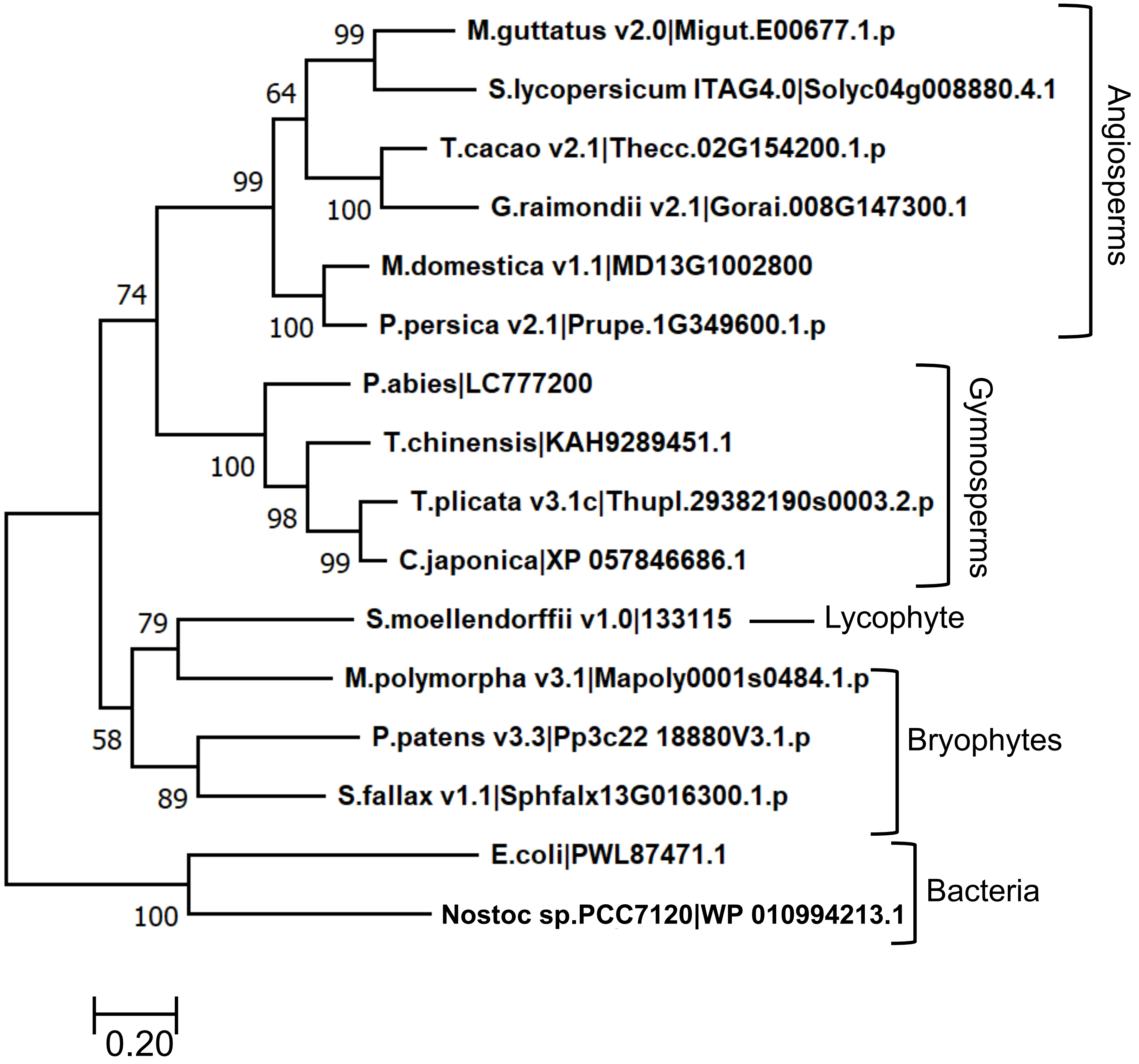

M.guttatus v2.0|Migut.E00677.1.p
S.lycopersicum ITAG4.0|Solyc04g008880.4.1
T.cacao v2.1|Thecc.02G154200.1.p
G.raimondii v2.1|Gorai.008G147300.1
M.domestica v1.1|MD13G1002800
P.persica v2.1|Prupe.1G349600.1.p
P.abies|LC777200
T.chinensis|KAH9289451.1
T.plicata v3.1c|Thupl.29382190s0003.2.p
C.japonica|XP 057846686.1
S.moellendorffii v1.0|133115
M.polymorpha v3.1|Mapoly0001s0484.1.p
P.patens v3.3|Pp3c22 18880V3.1.p
S.fallax v1.1|Sphfalx13G016300.1.p
E.coli|PWL87471.1
Nostoc sp.PCC7120|WP 010994213.1
Angiosperms
Gymnosperms
Lycophyte
Bryophytes
Bacteria
99
64
99
100
100
74
100
98
99
79
58
89
100
0.20

(h) MraY

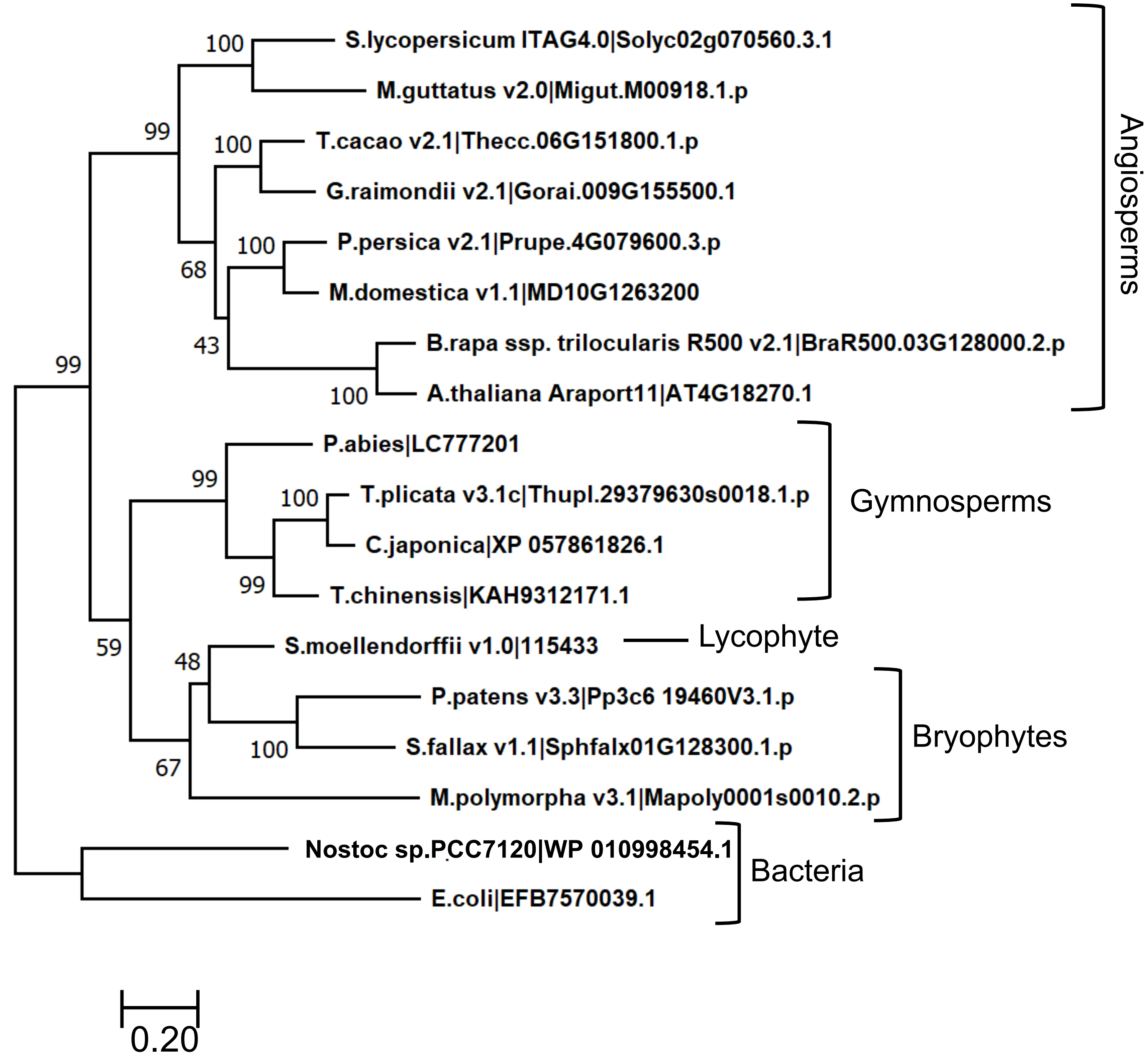

S.lycopersicum ITAG4.0|Solyc02g070560.3.1
M.guttatus v2.0|Migut.M00918.1.p
T.cacao v2.1|Thecc.06G151800.1.p
G.raimondii v2.1|Gorai.009G155500.1
P.persica v2.1|Prupe.4G079600.3.p
M.domestica v1.1|MD10G1263200
B.rapa ssp. trilocularis R500 v2.1|BraR500.03G128000.2.p
A.thaliana Araport11|AT4G18270.1
P.abies|LC777201
T.plicata v3.1c|Thupl.29379630s0018.1.p
C.japonica|XP 057861826.1
T.chinensis|KAH9312171.1
S.moellendorffii v1.0|115433
P.patens v3.3|Pp3c6 19460V3.1.p
S.fallax v1.1|Sphfalx01G128300.1.p
M.polymorpha v3.1|Mapoly0001s0010.2.p
Nostoc sp.PCC7120|WP 010998454.1
E.coli|EFB7570039.1
Angiosperms
Gymnosperms
Lycophyte
Bryophytes
Bacteria
100
99
100
100
68
43
100
99
99
100
99
59
48
100
67
0.20

(i) MurG

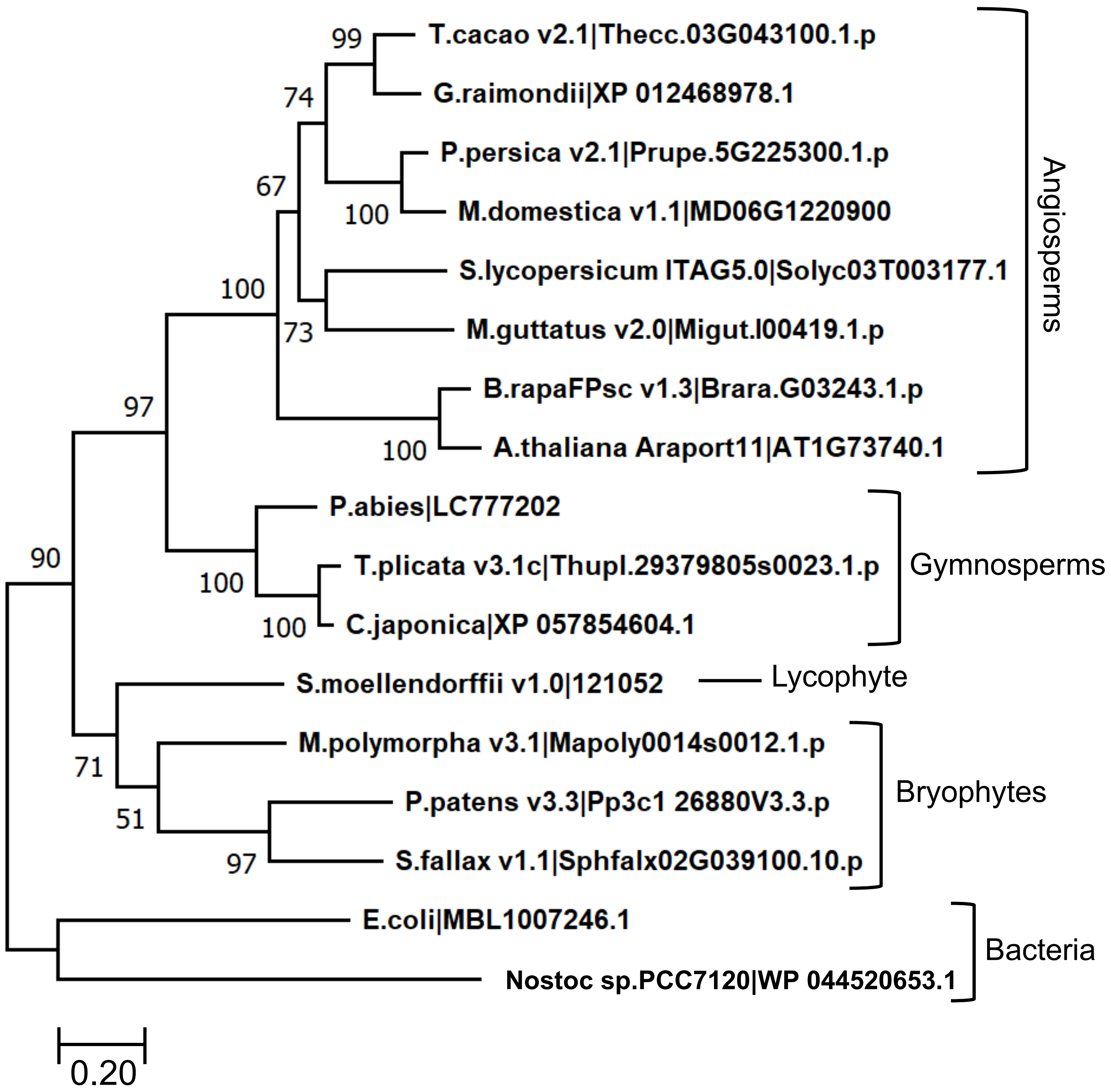

T.cacao v2.1|Thecc.03G043100.1.p
G.raimondii|XP 012468978.1
P.persica v2.1|Prupe.5G225300.1.p
M.domestica v1.1|MD06G1220900
S.lycopersicum ITAG5.0|Solyc03T003177.1
M.guttatus v2.0|Migut.I00419.1.p
B.rapaFPsc v1.3|Brara.G03243.1.p
A.thaliana Araport11|AT1G73740.1
P.abies|LC777202
T.plicata v3.1c|Thupl.29379805s0023.1.p
C.japonica|XP 057854604.1
S.moellendorffii v1.0|121052
M.polymorpha v3.1|Mapoly0014s0012.1.p
P.patens v3.3|Pp3c1 26880V3.3.p
S.fallax v1.1|Sphfalx02G039100.10.p
E.coli|MBL1007246.1
Nostoc sp.PCC7120|WP 044520653.1
Angiosperms
Gymnosperms
Lycophyte
Bryophytes
Bacteria
99
74
67
100
100
73
97
100
90
100
100
71
51
97
0.20

(j) MurJ

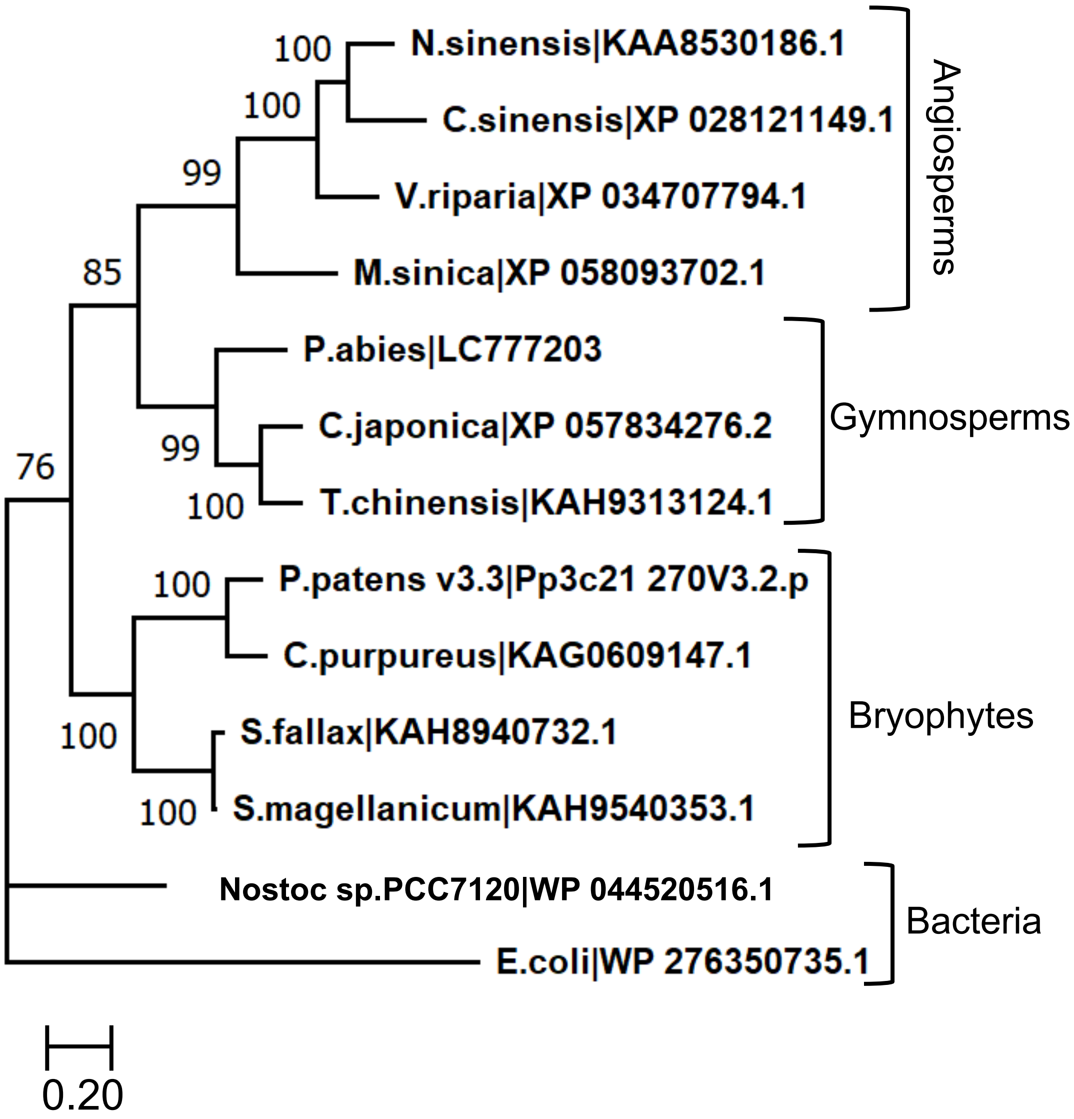

N.sinensis|KAA8530186.1
C.sinensis|XP 028121149.1
V.riparia|XP 034707794.1
M.sinica|XP 058093702.1
P.abies|LC777203
C.japonica|XP 057834276.2
T.chinensis|KAH9313124.1
P.patens v3.3|Pp3c21 270V3.2.p
C.purpureus|KAG0609147.1
S.fallax|KAH8940732.1
S.magellanicum|KAH9540353.1
Nostoc sp.PCC7120|WP 044520516.1
E.coli|WP 276350735.1
Angiosperms
Gymnosperms
Bryophytes
Bacteria
100
100
99
85
99
100
76
100
100
100
0.20

(k) PBP

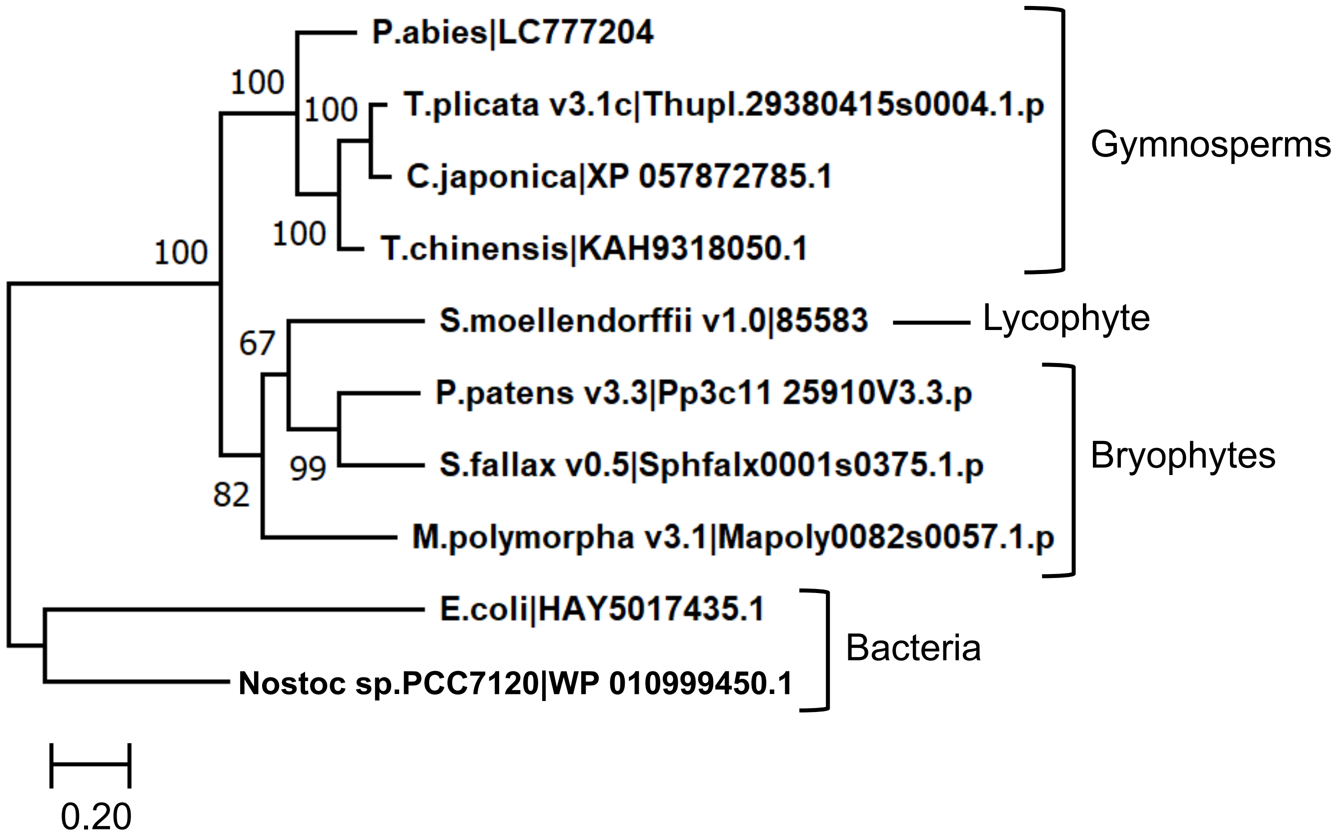

Figure. S1. Phylogenetic trees for Mur proteins in plants.
Phylogenetic trees were constructed with the amino-acid sequences of selected plant species and bacteria *Anabaena* sp. PCC7120 and *E. coli* using the maximum likelihood method and JTT matrix-based model in MEGA11 (Jones et al. 1992, Tamura et al. 2021, Stecher et al. 2020). Trees with the highest log likelihood are shown. The percentage of trees in which the associated taxa clustered together is shown next to the branches. Initial tree(s) for the heuristic search were obtained automatically by applying Neighbor-Join and BioNJ algorithms to a matrix of pairwise distances estimated using the JTT model and then selecting the topology with superior log likelihood value. The tree is drawn to scale, with branch lengths measured in the number of substitutions per site. Phylogenetic trees for MurA (a), MurB (b), MurC (c), MurD (d), MurE (e), MurF (f), MurG (g), MraY (h), MurJ (i), DDL (j) and PBP (k) are shown.

Suppl. Figure 2. Mur gene amino acid alignment
(a) MurA

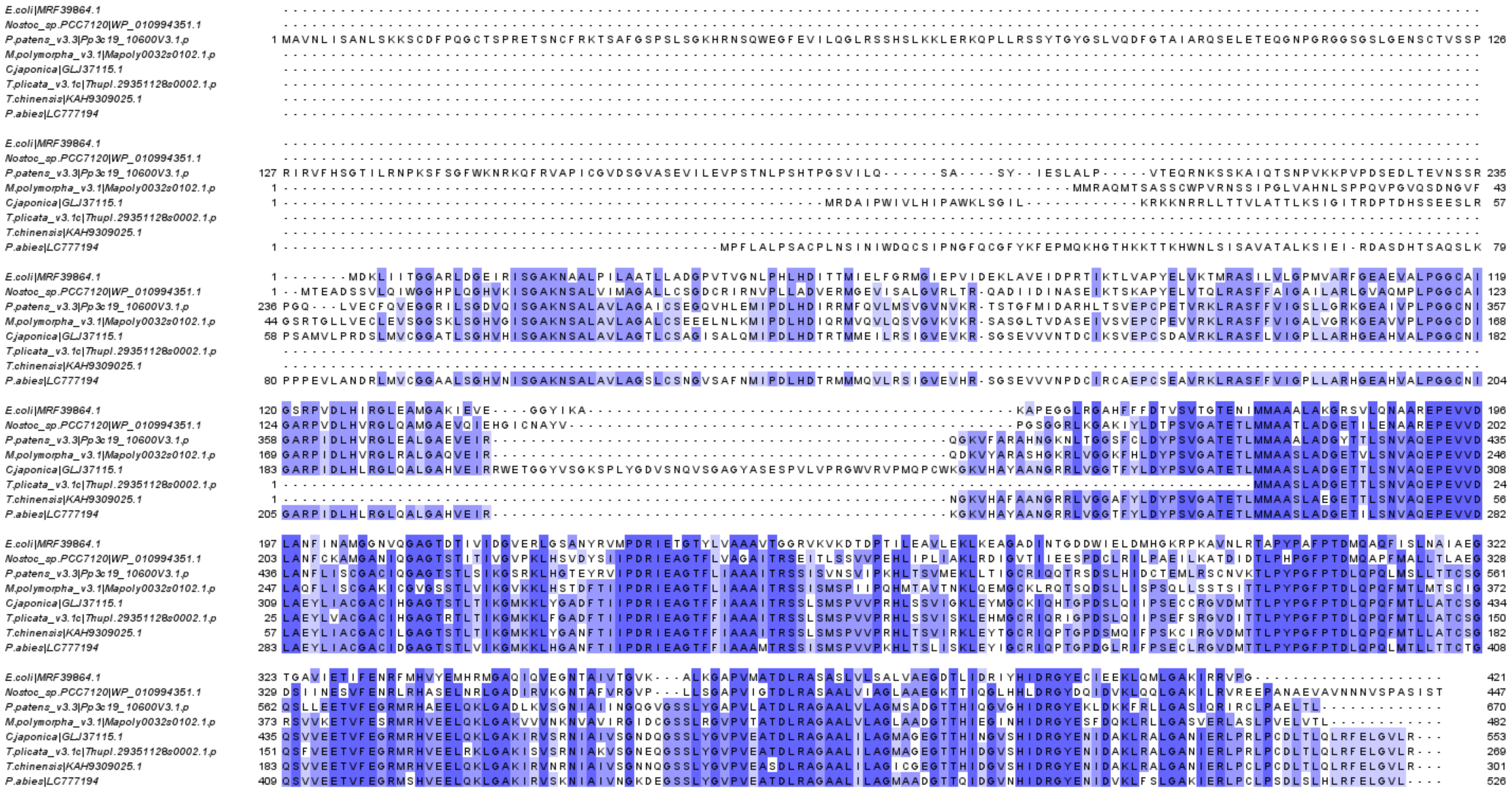

(b) MurB

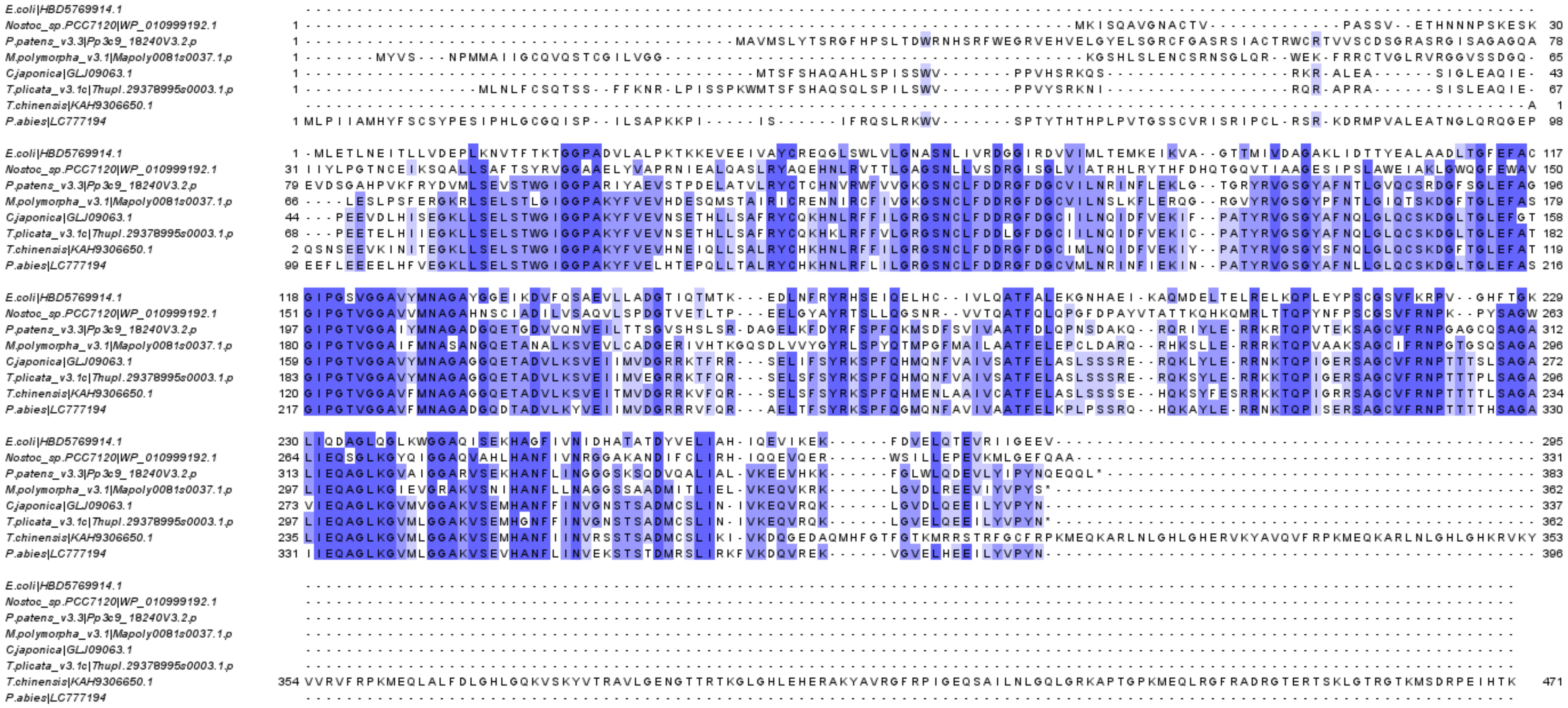
E.coli|HBD5769914.1
Nostoc_sp.PCC7120|WP_010999192.1
P.patens_v3.3|Pp3c9_18240V3.2.p
M.polymorpha_v3.1|Mapoly0081s0037.1.p
C.japonica|GLJ09063.1
T.plicata_v3.1c|Thupl.29378995s0003.1.p
T.chinensis|KAH9306650.1
P.abies|LC777194

(c) MurC

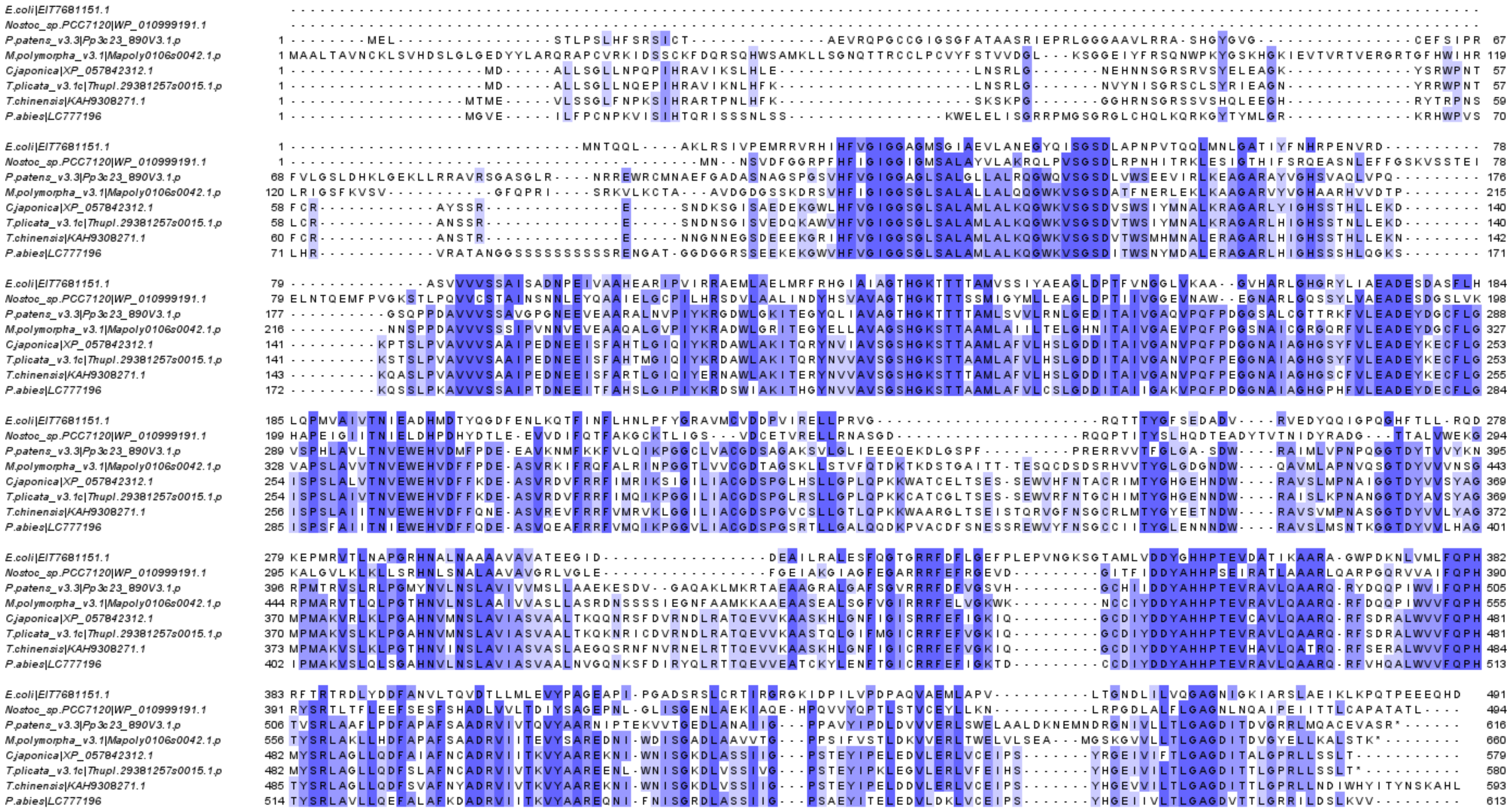
E.coli|EIT7681151.1
Nostoc_sp.PCC7120|WP_010999191.1
P.patens_v3.3|Pp3c23_890V3.1.p
M.polymorpha_v3.1|Mapoly0106s0042.1.p
C.japonica|XP_057842312.1
T.plicata_v3.1c|Thupl.29381257s0015.1.p
T.chinensis|KAH9308271.1
P.abies|LC777196

(d) MurD

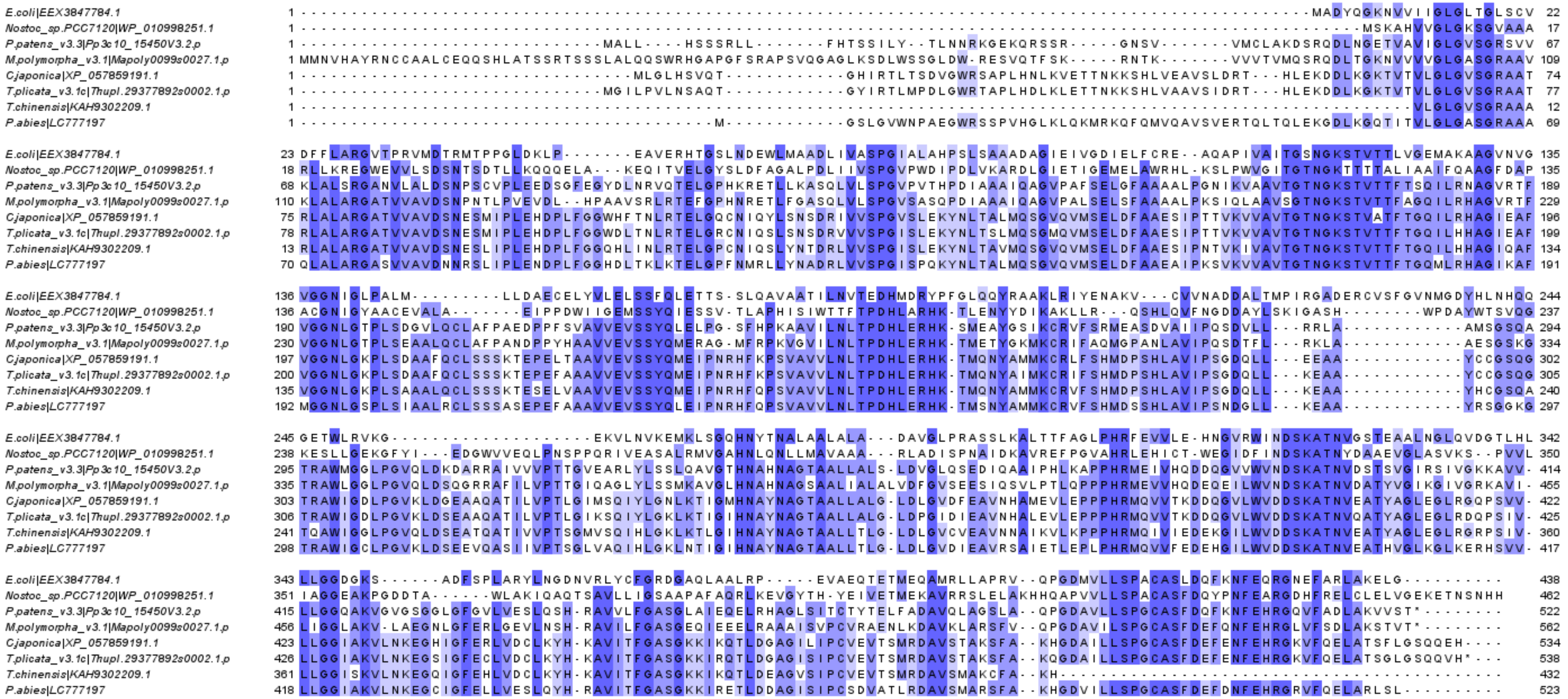

E.coli|EEX3847784.1
Nostoc_sp.PCC7120|WP_010998251.1
P.patens_v3.3|Pp3c10_15450V3.2.p
M.polymorpha_v3.1|Mapoly0099s0027.1.p
C.japonica|XP_057859191.1
T.plicata_v3.1c|Thupl.29377892s0002.1.p
T.chinensis|KAH9302209.1
P.abies|LC777197

(e) MurE

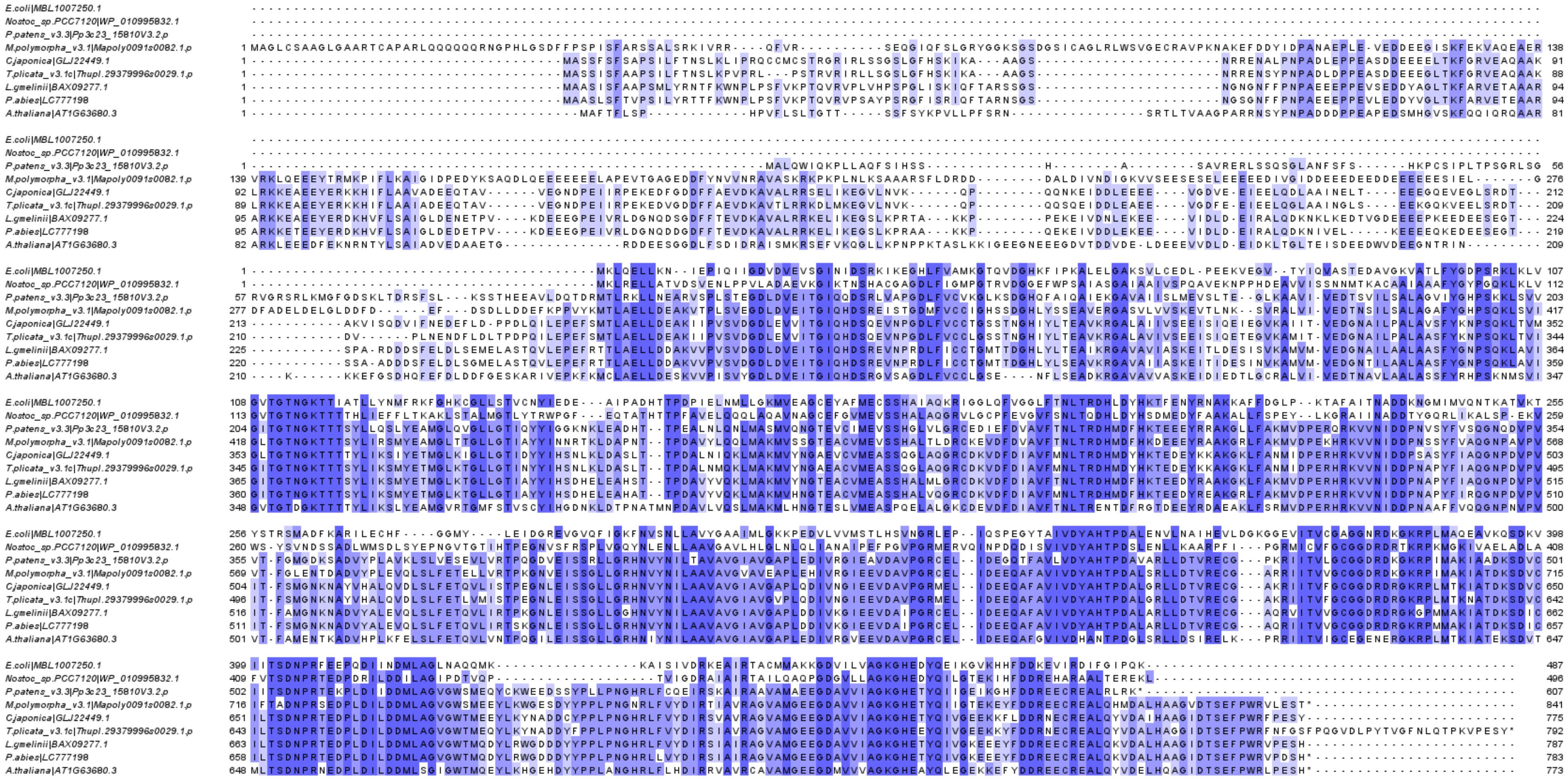
E.coli|MBL1007250.1
Nostoc_sp.PCC7120|WP_010995832.1
P.patens_v3.3|Pp3c23_15810V3.2.p
M.polymorpha_v3.1|Mapoly0091s0082.1.p
C.japonica|GLJ22449.1
T.plicata_v3.1c|Thupl.29379996s0029.1.p
L.gmelinii|BAX09277.1
P.abies|LC777198
A.thaliana|AT1G63680.3

(f) MurF

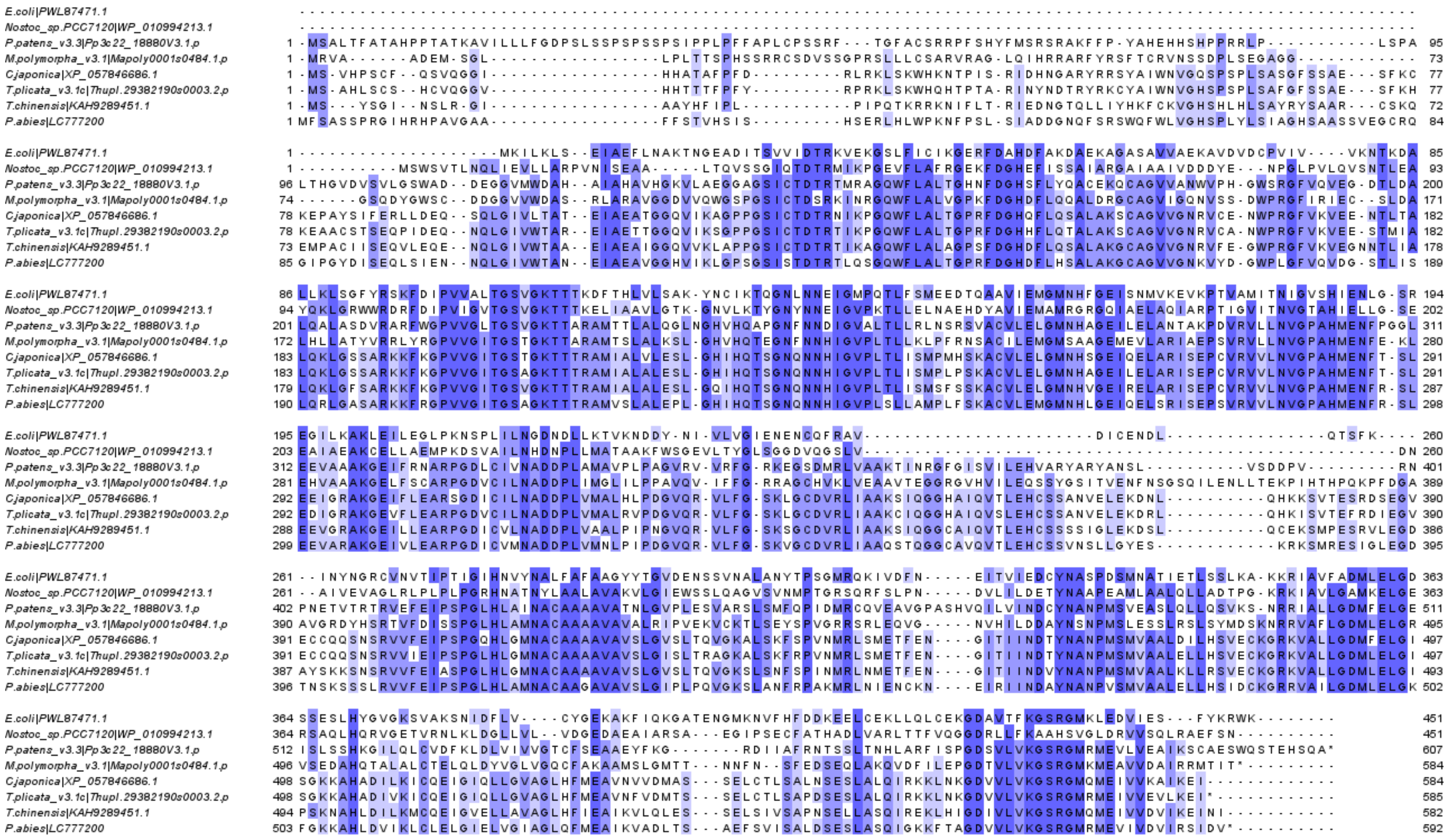
E.coli|PWL87471.1
Nostoc_sp.PCC7120|WP_010994213.1
P.patens_v3.3|Pp3c22_18880V3.1.p
M.polymorpha_v3.1|Mapoly0001s0484.1.p
C.japonica|XP_057846686.1
T.plicata_v3.1c|Thupl.29382190s0003.2.p
T.chinensis|KAH9289451.1
P.abies|LC777200

(g) MurG

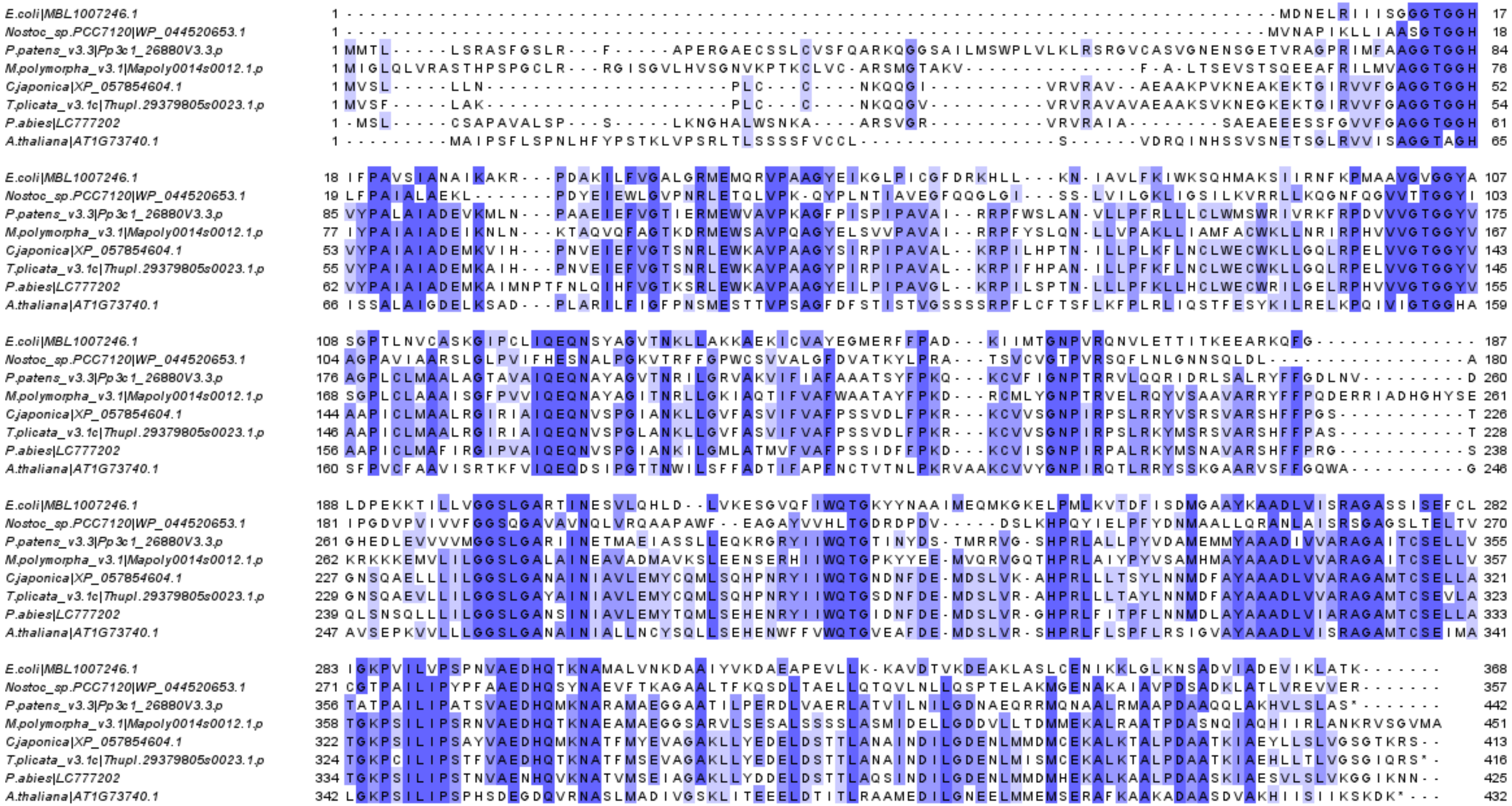

E.coli|MBL1007246.1
Nostoc_sp.PCC7120|WP_044520653.1
P.patens_v3.3|Pp3c1_26880V3.3.p
M.polymorpha_v3.1|Mapoly0014s0012.1.p
C.japonica|XP_057854604.1
T.plicata_v3.1c|Thupl.29379805s0023.1.p
P.abies|LC777202
A.thaliana|AT1G73740.1

(h) MraY

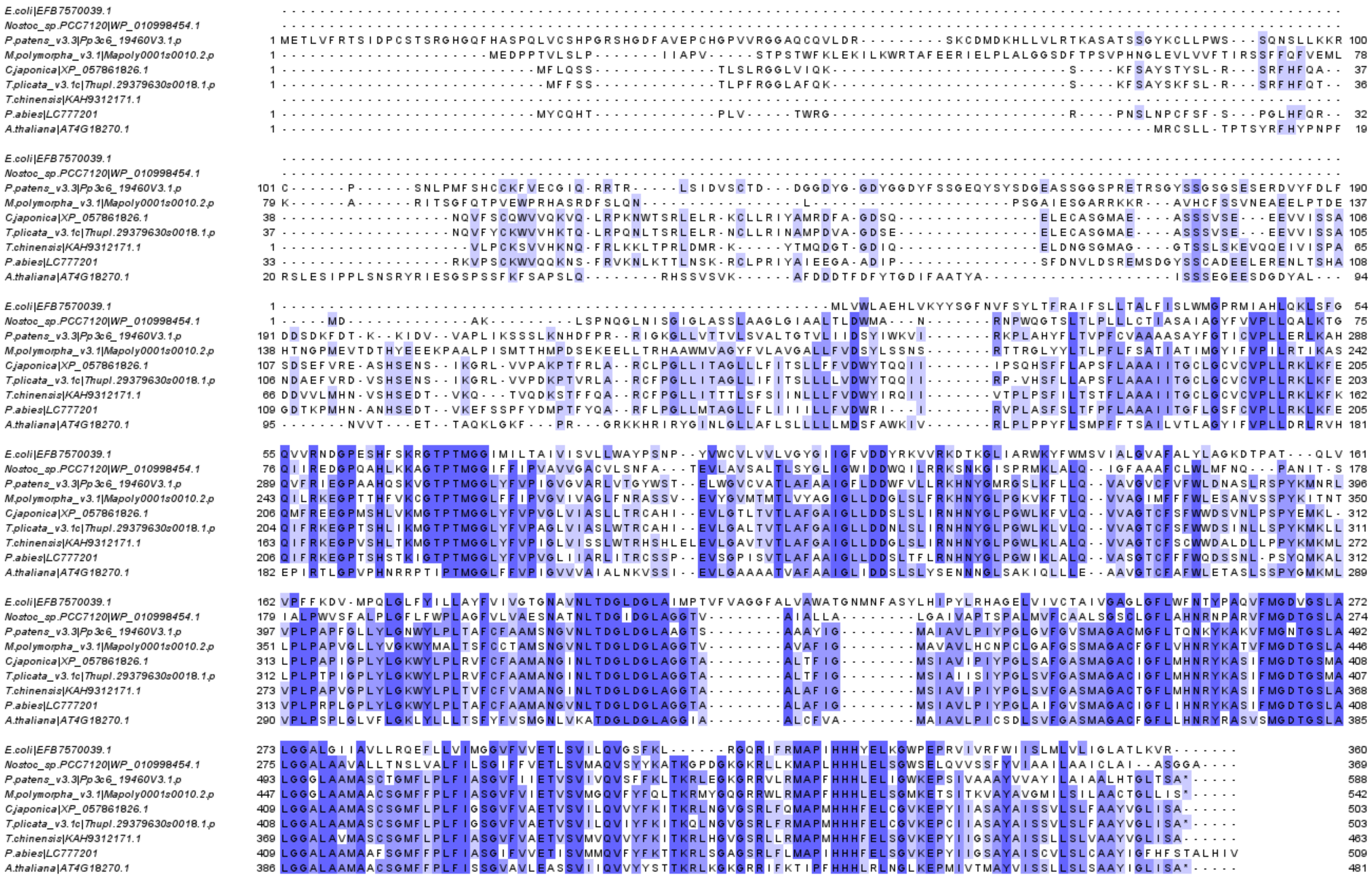
E.coli|EFB7570039.1
Nostoc_sp.PCC7120|WP_010998454.1
P.patens_v3.3|Pp3c6_19460V3.1.p
M.polymorpha_v3.1|Mapoly0001s0010.2.p
C.japonica|XP_057861826.1
T.plicata_v3.1c|Thupl.29379630s0018.1.p
T.chinensis|KAH9312171.1
P.abies|LC777201
A.thaliana|AT4G18270.1

(i) MurJ

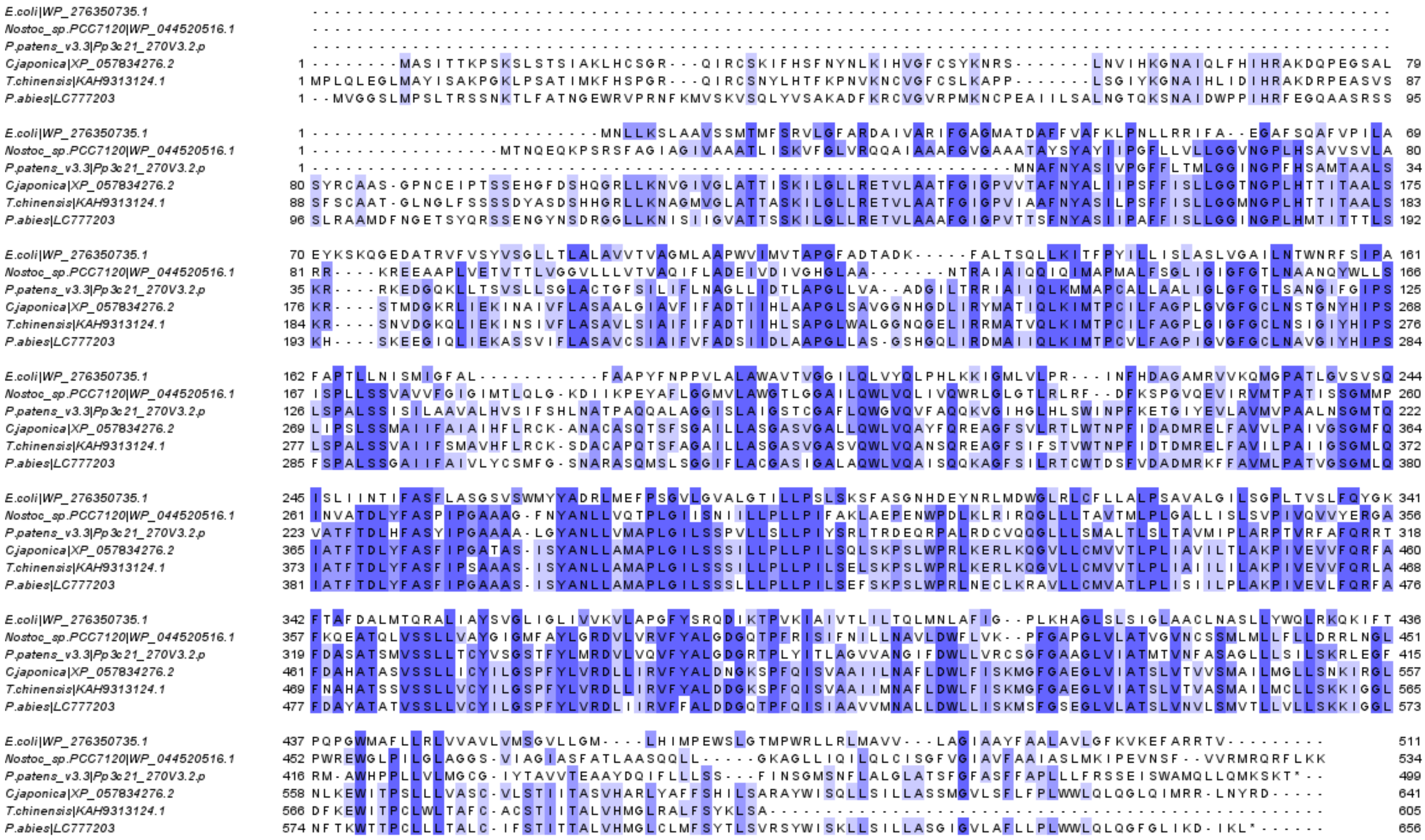
E.coli|WP_276350735.1
Nostoc_sp.PCC7120|WP_044520516.1
P.patens_v3.3|Pp3c21_270V3.2.p
C.japonica|XP_057834276.2
T.chinensis|KAH9313124.1
P.abies|LC777203

(j) DDL

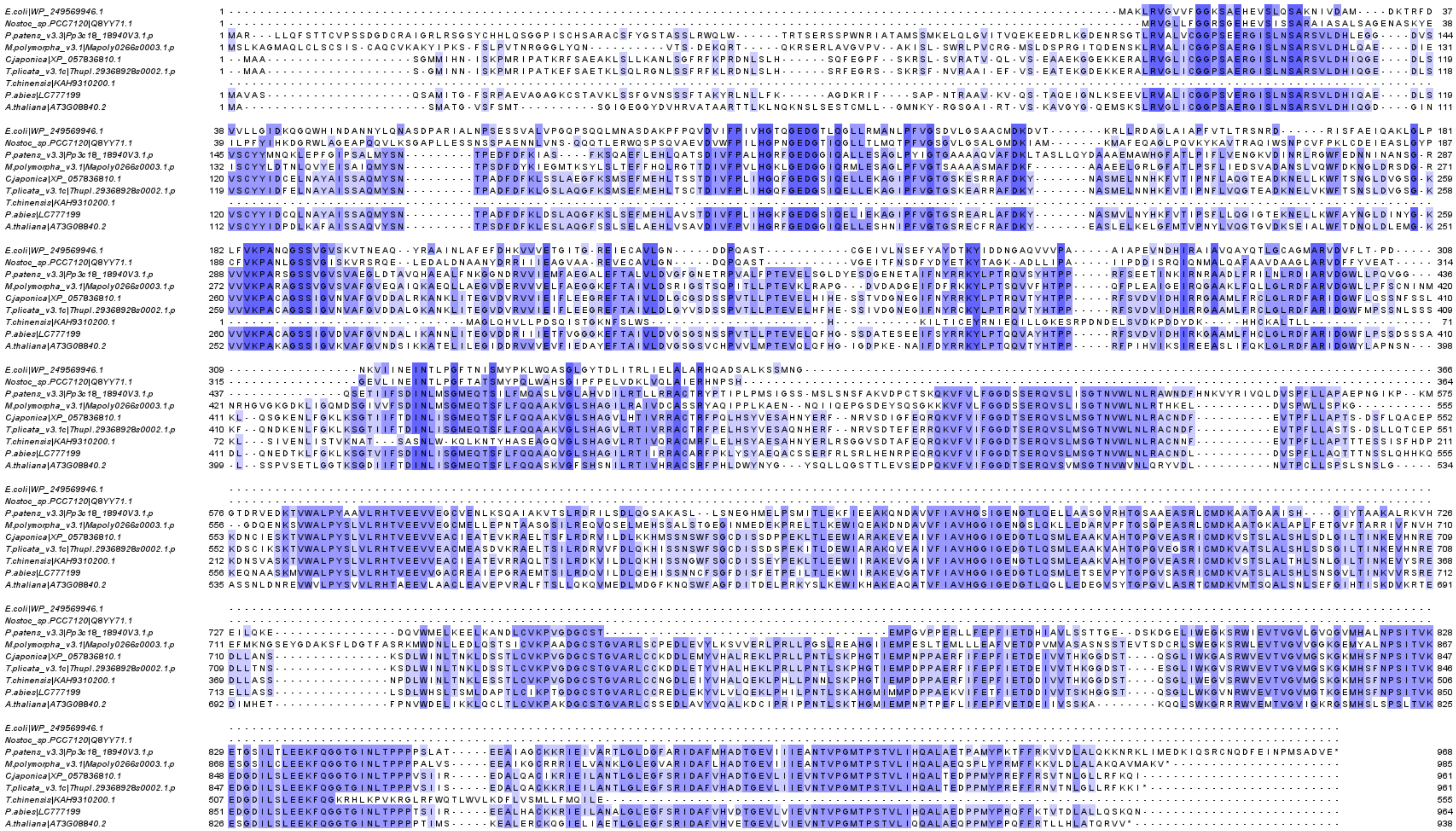

E.coli|WP_249569946.1
Nostoc_sp.PCC7120|Q8YY71.1
P.patens_v3.3|Pp3c18_18940V3.1.p
M.polymorpha_v3.1|Mapoly0266s0003.1.p
C.japonica|XP_057836810.1
T.plicata_v3.1c|Thupl.29368928s0002.1.p
T.chinensis|KAH9310200.1
P.abies|LC777199
A.thaliana|AT3G08840.2

(k) PBP

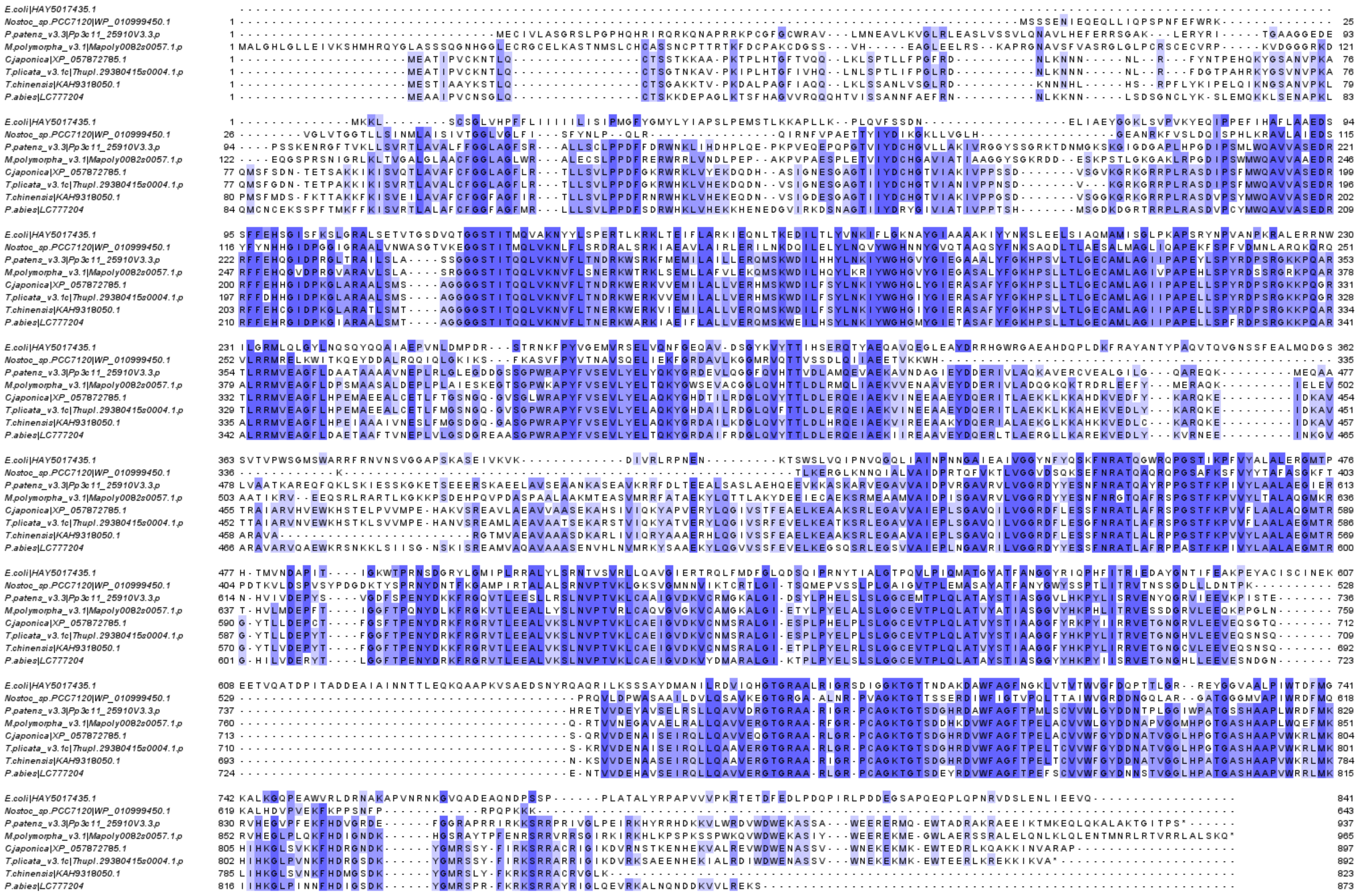
E.coli|HAY5017435.1
Nostoc_sp.PCC7120|WP_010999450.1
P.patens_v3.3|Pp3c11_25910V3.3.p
M.polymorpha_v3.1|Mapoly0082s0057.1.p
C.japonica|XP_057872785.1
T.plicata_v3.1c|Thupl.29380415s0004.1.p
T.chinensis|KAH9318050.1
P.abies|LC777204

Figure S2. Mur gene amino acid alignment.

Comparison of the amino-acid sequences encoded by the *Mur* genes of *P. abies* with those of other species as aligned by (Clustal W). Mur proteins were used from the selected species: *Physcomitrium patens*, *M. polymorpha*, *A. thaliana*, *Anabaena* (Nostoc) sp. PCC7120 and *E. coli* in the data for phylogenetic trees (Figure S1). Other gymnosperm Mur proteins in Figure S1 were also added. Blue coloring followed the default settings of the JalView software (Waterhouse et al. 2009). Amino-acid alignments for MurA (a), MurB (b), MurC (c), MurD (d), MurE (e), MurF (f), MurG (g), MraY (h), MurJ (i), DDL (j) and PBP (k) are shown.

(a)

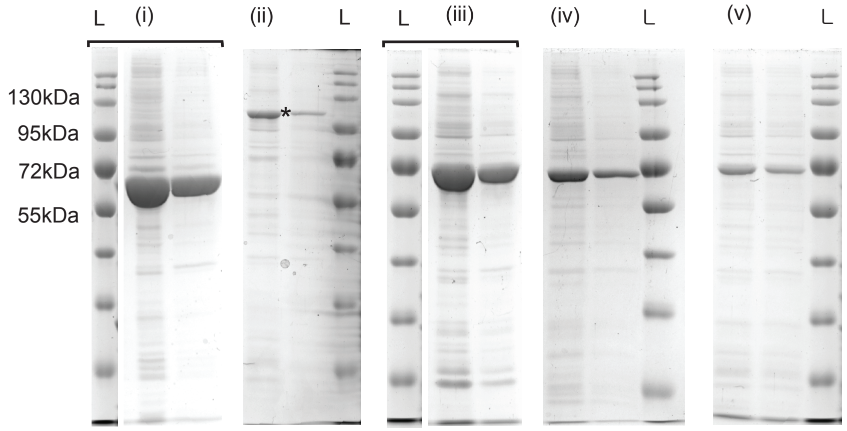

(b)

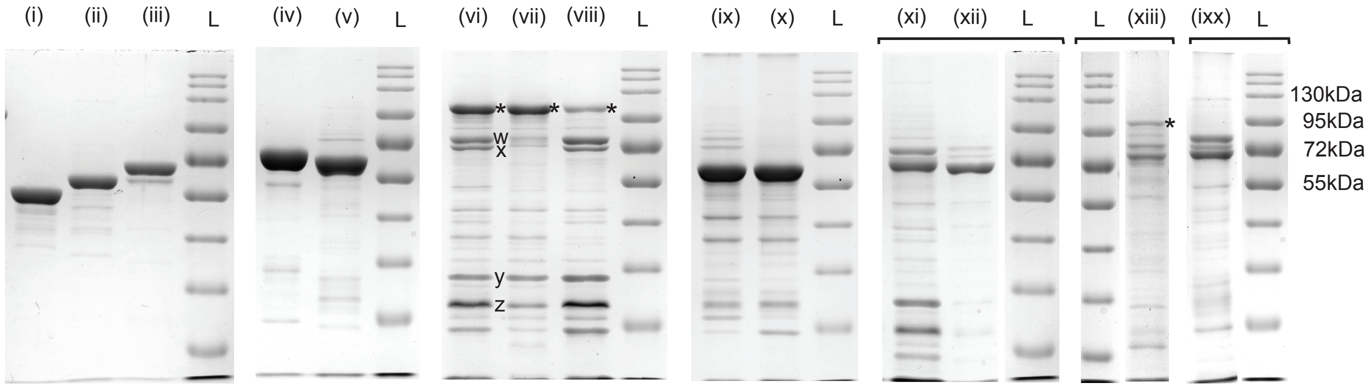

Figure S3. PAGE (10%) of MurE proteins used for activity studies. (L) Color Prestained Protein Standard (10–250 kDa) (NEBiolabs P7719S).

(a) Proteins prepared by heterologous expression in baculovirus-infected Spodoptera frugiperda (Sf9) cells. Proteins were purified using Ni affinity and the two 300 mM imidazole eluted fractions visualised: (i) PpMurE-TP_AH (64.3 kDa, D7), (ii) HA_PaMurE-TP (87.7 kDa, B1), (iii) HA_PaMurE-SATD (63.7 kDa, J6), (iv) PaMurE-SATD_AH (63.7 kDa, E7), (v) HA_LgMurE-SATD (63.3 kDa, B2).

(b) Proteins were prepared by heterologous expression in E. coli and purified by Ni affinity and, for some proteins, size exclusion chromatography. (i) P. aeruginosa MurE (51.2 kDa), (ii) H_AnMurE (56.6 kDa, 46T), (iii) H_PpMurE-TP (62.6 kDa, 22B), (iv) PpMurE-TP_AH (64.3 kDa, 1.1T), (v) PpMurE-TP (60.5 kDa, 1.1T minus His tag), (vi) HA_PaMurE-TP (87.7 kDa, B1 Fn 8-14), (vii) HA_PaMurE-TP (87.7 kDa, B1 Fn 15-20), (viii) PaMurE-TP_AH (84.5 kDa, E6), (ix) H_PaMurE-SATD (60.5 kDa, H S200 column fractions 4-8), (x) H_PaMurE-SATD (60.5 kDa, H S200 column fractions 9-12), (xi) HA_PaMurE-SATD (63.7 kDa, J6), (xii) PaMurE-SATD_AH (63.7 kDa, E7), (xiii) H_LgMurE-TP (82.9 kDa, G), (ixx) LgMurE-SATD_AH (63.2 kDa, 7.3T). Names of protein tags have been abbreviated: His to H, His_Avi to HA_ and Avi_His to _AH and figures in parentheses represent construct names. All MurE-TP proteins (ie. they encompassed the SATD) consistently migrated above their expected masses, at greater than 95kDa, whether prepared from S. frugiperda or E. coli. Because this was observed for proteins from both of the heterologous expression hosts it was considered probable that it was a consequence of the disordered structure of the SATD, rather than any post-translational modification. For the E. coli expressed SATD-containing proteins LCMS confirmed that the larger bands, indicated by asterisks, were the gymnosperm MurE, and the major contaminants were ArnA and HtpG (w), GlmS (x), ribosomal protein L2 (y) and SlyD (z). Protein markers, when not run in wells adjacent to the proteins of interest, have been presented as separate images alongside the relevant proteins from within the same gel and grouped by brackets.

(a)

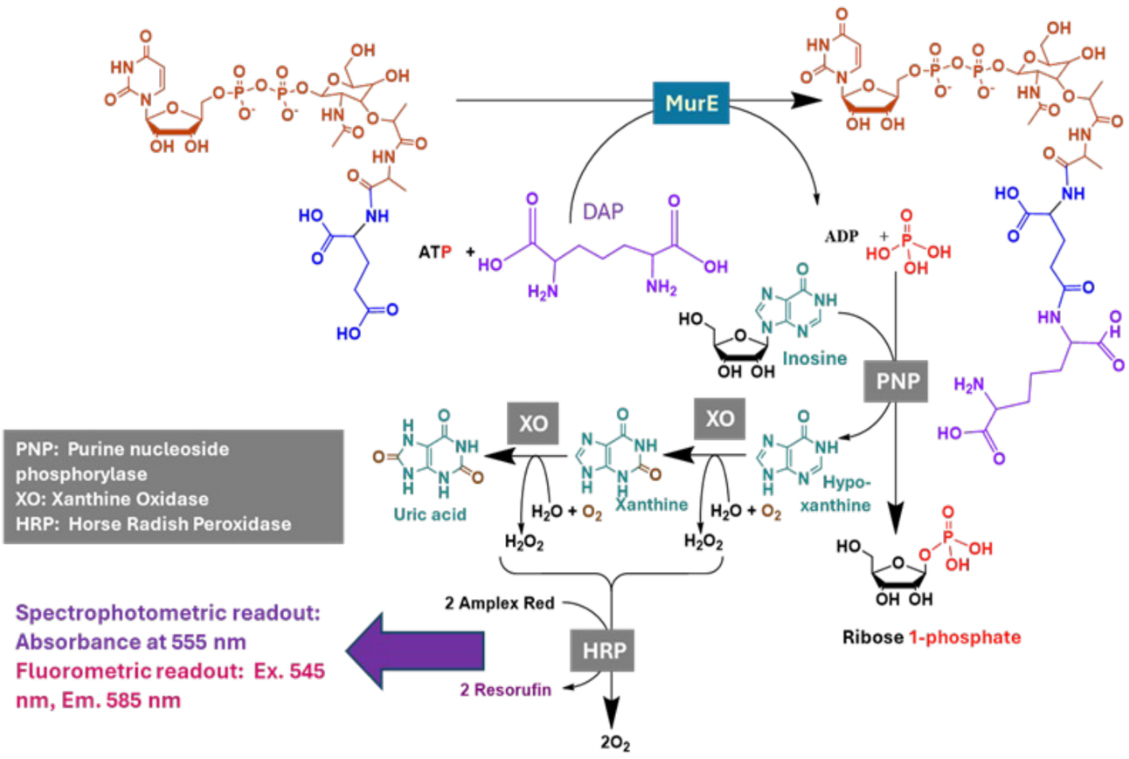

(b)

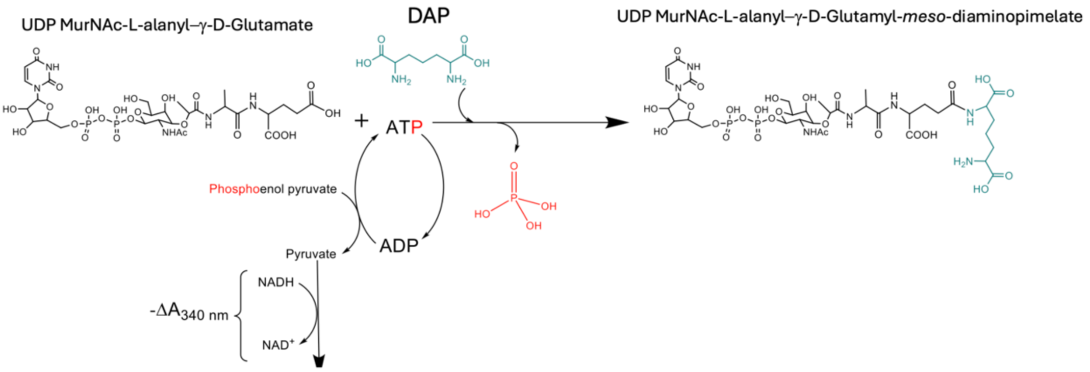

Figure S4. Enzymatic assays of UDP-N-acetylmuramoyl-L-alanyl-D-glutamate--2,6-diaminopimelate ligase (MurE) proteins.

(a) Assay scheme for continuous photometric assay of MurE dependent phosphate generation, coupled to phosphorolysis of inosine to hypoxanthine, which is subsequently oxidized to uric acid with concomitant generation of hydrogen peroxide which is coupled to conversion of Amplex Red to resorufin, generating either a spectrophotometric or a fluorometric output. (b) Assay scheme for the photometric assay of MurE dependent ADP generation, coupled to phosphoenolpyruvate kinase and lactate dehydrogenase generation of NAD from NADH and a concomitant loss of absorbance at 340 nm.

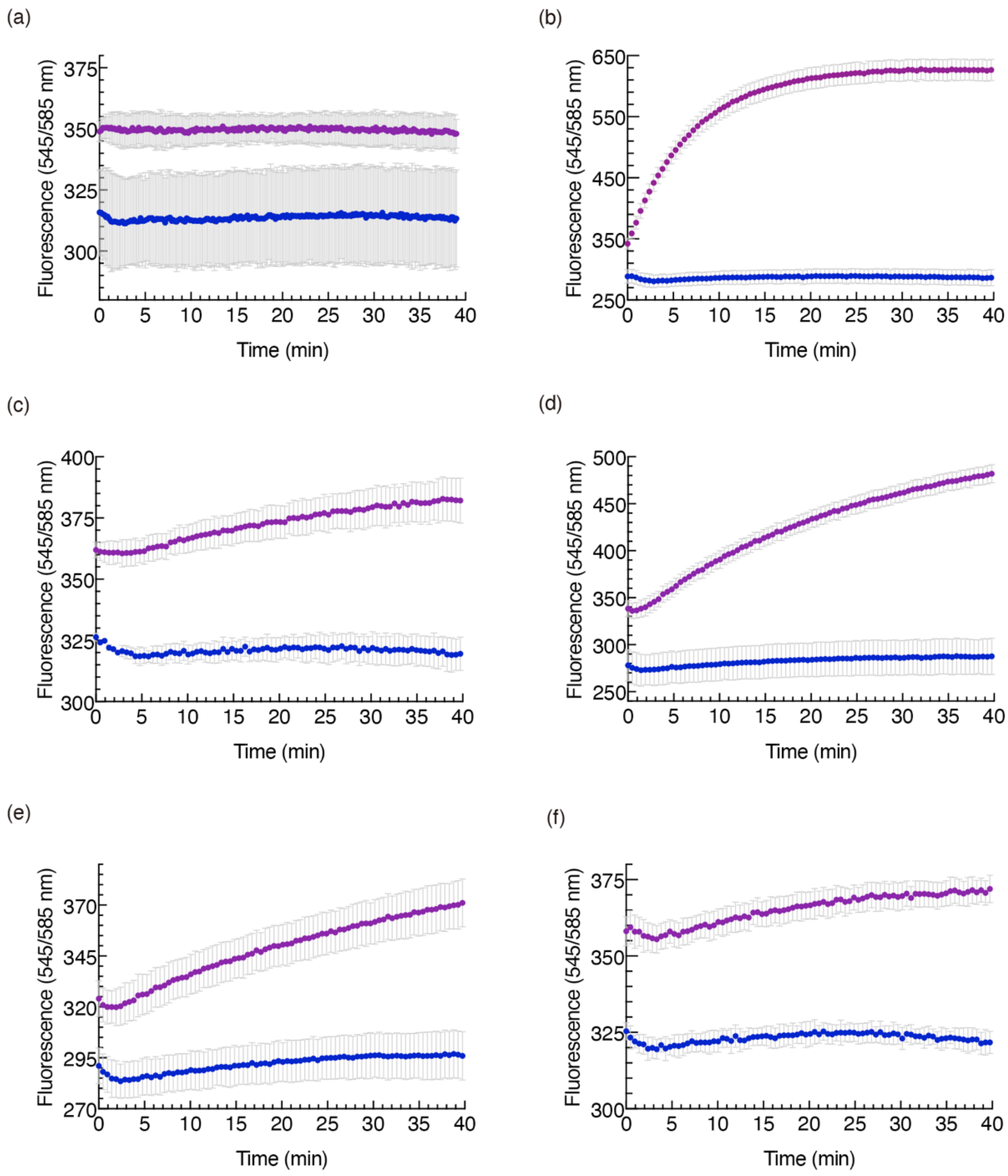

Figure S5. Time courses of resorufin fluorescence from Amplex Red, consequent on Pi release in reactions catalysed by plant MurE ligases.

Plant MurE proteins were prepared from baculovirus-infected *Spodoptera frugiperda* (Sf9) cells and assayed as described. Data are the means of triplicate fluorescence measurements of phosphate release ± standard error. The chloroplast transit peptide sequences were eliminated in all constructions (-TP), as was an amino terminal streptophyte specific domain (-SATD) in some constructions, all proteins included an hexahistidine and Avi-tag. Blue data points and purple data points: mean fluorescence measured in the respective absence and presence of 8 mM D,L-DAP. Error bars are grey for all traces. Data in part reflect the different protein concentrations of the enzymes. (a) Buffer, (b) PpMurE-TP_Avi_His (D7), (c) His_Avi_PaMurE-TP (B1), (d) His_Avi_PaMurE-SATD (J6), (e) PaMurE-SATD_Avi_His (E7), (f) His_Avi_LgMurE-SATD (B2).

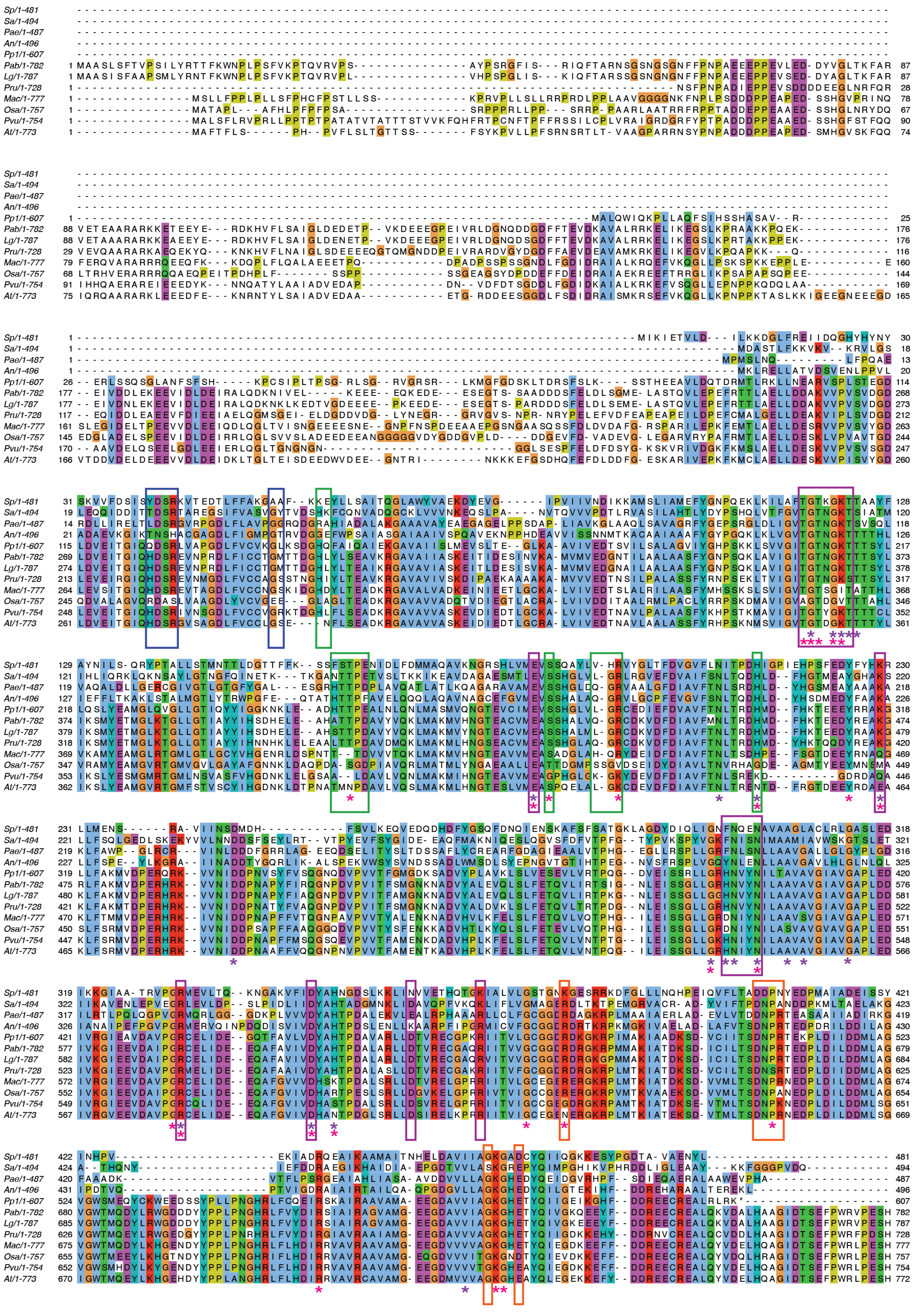
Sp/1-481
Sa/1-494
Pae/1-487
An/1-496
Pp1/1-607
Pab/1-782
Lg/1-787
Pru/1-728
Mac/1-777
Osa/1-757
Pvu/1-754
At/1-773

Figure S6. Multiple sequence alignment of bacterial and plant MurE ligases and homologs with reported ligand binding residues identified.

Alignment used Clustal Omega (EMBL-EBI) (Madeira et al., 2019) and was displayed using Jalview (Waterhouse et al., 2009) with Clustal designated colours. Gram positive, L-Lys-specific MurE: *Streptococcus pneumoniae* (Sp: WP_000590315.1 NCBI) and *Staphylococcus aureus* (Sa: 700699 WP_000340119.1 NCBI). Gram negative D, L-DAP-specific MurE: *Pseudomonas aeruginosa* (Pae: WP_003112751.1 NCBI) and *Anabaena nostoc* PCC7120 (An: WP_010995832.1 NCBI). Plant D, L-DAP-specific MurE: *Physcomitrium patens* (Pp1: Pp3c24_18820V3.2 v3.3 Phytozome), *Picea abies* (Pab: BER90440.1 Genbank). Plant MurE homolog sequences: *Larix gmelinii* (Lg: BAX09277.1 Genbank), *Podocarpus rubra* (Pru: XLGK_scaffold_2012189, OneKP), *Musa acuminata* (Mac: CAG1838261.1 Genbank subsp. *malaccensis*), *Oryza sativa* (Osa: XP_015614047.1 NCBI subsp. *japonica*), *Phaseolus vulgaris* (Pvu: XP_068470876.1 NCBI), *Arabidopsis thaliana* (At: AT1G63680.1 TAIR). Accession numbers and databases in parentheses. Bacterial MurE residues with published interactions to ligands UDP (blue), MurNAc sugar (green), ATP or ADP (mauve) and D, L-DAP (orange) have been boxed (*Escherichia coli* (Gordon et al., 2001), *Mycobacterium tuberculosum* (Basavannacharya et al., 2010; Maitra et al., 2019) or *Staphylococcus aureus* (Ruane et al., 2013)). Blue asterisks indicate residues common to the Mur ligase family, which includes folylpolyglutamate synthetase, cyanophycin synthetase and the capB enzyme from Bacillales (Gordon et al., 2001; Smith, 2006) and pink asterisks indicate residues common to MurC, D, E and F ligases (Basavannacharya et al., 2010).

Figure S7. LCMS data for post-translational modifications of MurE ligases expressed in baculovirus-infected *S. frugiperda* (Sf9) and *E. coli.*
Proteins were excised from PAGE gels, trypsin digested and analysed by MS/MS. Modifications of amino acids in fragments with strongly confirmatory spectra were highlighted by hand and are presented on Clustal Omega alignments (EMBL-EBI) for (a) baculovirus-infected Sf9-expressed proteins and (c) *E. coli*-expressed proteins. Modifications included carbamylation (+43.00582 Da, blue), ubiquitinylation (+114.042927 Da, red), methylation (+14.01565 Da, green), acetylation (+42.01056 Da, orange) and phosphorylation (+79.96633 Da, pink), the most frequent modification being highlighted. Red arrows indicate a lysine, carbamylated in *Mycobacterium tuberculosis* and *E. coli* MurE, and predicted to interact, via two water molecules, with one of two Mg^{2+} ions (Dementin et al., 2001). The blue arrow indicates a lysine that is similarly, but even more closely, proximal to the second Mg^{2+} ion, as predicted by Pymol 3.1 alignment of PpMurE to *M. tuberculosis* MurE (2xja). Red boxes correspond to areas where an estimate of the percentage modification was calculated for proteins from (b) baculovirus-infected Sf9 cells and (d) *E. coli.* Green boxes indicate the most methylated lysines in the SATD and blue boxes highlight the D,L-DAP-specific DNPR motif. The percentage of modified fragments relative to total fragments were related to the MS/MS Total Ion Count (TIC) for trypsin-digested fragments spanning all or part of the red boxed areas. Where a fragment was modified by the same modification at more than one residue within a particular fragment that TIC was counted twice. (e). Representative MS/MS spectra for modifications mentioned in the text

(a) Alignment of proteins from baculovirus-infected (Sf9) insect cells.

```
PpMurE-TP_Avi_His_D7          ------------------------------------------------------------   0
PaMurE-SATD_Avi_His_E7        ------------------------------------------------------------   0
His_Avi_LgMurE-SATD_B2        MAHHHHHHSSGLEVLFQGPGGLNDIFEAQKIEWHEGENLYFQSGT---------------   45
His_Avi_PaMurE-TP_B1          MAHHHHHHSSGLEVLFQGPGGLNDIFEAQKIEWHEGENLYFQSGSAYPSRGFISRIQFTA   60
His_Avi_PaMurE-SATD_J6        MAHHHHHHSSGLEVLFQGPGGLNDIFEAQKIEWHEGENLYFQSGT---------------   45

PpMurE-TP_Avi_His_D7          ------------------------------------------------------------   0
PaMurE-SATD_Avi_His_E7        ------------------------------------------------------------   0
His_Avi_LgMurE-SATD_B2        ------------------------------------------------------------   45
His_Avi_PaMurE-TP_B1          RNSGSNGSGNFFPNPAEEEPPEVLEDDYVGLTKFARVETEAARARKKETEEYERDKHVFL   120
His_Avi_PaMurE-SATD_J6        ------------------------------------------------------------   45

PpMurE-TP_Avi_His_D7          ------------------------------------------------------------   0
PaMurE-SATD_Avi_His_E7        ------------------------------------------------------------   0
His_Avi_LgMurE-SATD_B2        ------------------------------------------------------------   45
His_Avi_PaMurE-TP_B1          SAIGLDEDETPVKDEEEGPEIVRLDGNQDDGDFFTEVDKAVALRRKELIKEGSLKPRAAK   180
His_Avi_PaMurE-SATD_J6        ------------------------------------------------------------   45

PpMurE-TP_Avi_His_D7          -------------------------------------------MGFG-DSKLTDRSFSLK   16
PaMurE-SATD_Avi_His_E7        ------------------------------------------------------------   0
His_Avi_LgMurE-SATD_B2        ------------------------------------------------------------   45
His_Avi_PaMurE-TP_B1          KPQEKEIVDDLEKEEVIDLDEIRALQDKNIVELKEEEEQKEDEESEGTSSAADDDSFELD   240
His_Avi_PaMurE-SATD_J6        ------------------------------------------------------------   45

                                              1
PpMurE-TP_Avi_His_D7          SST---HEEAVLDQTDRMTLRKLLNEARVSPLSTEGDLDVEITGIQQDSRLVAPGDLFVC   73
PaMurE-SATD_Avi_His_E7        ----------------MTTLAELLDDAKVVPVSVDGDLDVEITGIQHDSREVNPRDLFIC   44
His_Avi_LgMurE-SATD_B2        -----------------TLAELLDDAKVVPVSVDGDLDVEITGIQHDSREVNPRDLFIC   87
His_Avi_PaMurE-TP_B1          LSGMELASTQVLEPEFRTTLAELLDDAKVVPVSVDGDLDVEITGIQHDSREVNPRDLFIC   300
His_Avi_PaMurE-SATD_J6        -----------------TLAELLDDAKVVPVSVDGDLDVEITGIQHDSREVNPRDLFIC   87
                                               ** :**::*:* *:*.:***********:*** * * ***:*

PpMurE-TP_Avi_His_D7          VKGLKSDGHQFAIQAIEKGAVAIISLMEVSLTEG--LKAAVIVEDTSVILSALAGVIYGH   131
PaMurE-SATD_Avi_His_E7        CTGMTTDGHLYLSEAVKRGAVAIIASKEITIDESINVKAMVMVEDGNTILAALAASFYGN   104
His_Avi_LgMurE-SATD_B2        CTGMTTDGHLYLTEAIKRGAVAVIASKEITLDESISVKAMVMVEDGNAILAALAASFYGN   147
His_Avi_PaMurE-TP_B1          CTGMTTDGHLYLSEAVKRGAVAIIASKEITIDESINVKAMVMVEDGNTILAALAASFYGN   360
His_Avi_PaMurE-SATD_J6        CTGMTTDGHLYLSEAVKRGAVAIIASKEITIDESINVKAMVMVEDGNTILAALAASFYGN   147
                               .*:.:*** :  :*::****:*:  *::: *.  :** *:*** ..**:***. :**:

PpMurE-TP_Avi_His_D7          PSKKLSVVGITGTNGKTTTSYLLQSLYEAMGLQVGLLGTIQYYIGGKNKLEADHTTPEAL   191
PaMurE-SATD_Avi_His_E7        PSQKLAVIGITGTNGKTTTSYLIKSMYETMGLKTGLLGTIAYYIHSDHELEAHATTPDAV   164
His_Avi_LgMurE-SATD_B2        PSQKLAVIGITGTNGKTTTSYLIKSMYETMGLKTGLLGTIAYYIHSDHELEAHSTTPDAV   207
His_Avi_PaMurE-TP_B1          PSQKLAVIGITGTNGKTTTSYLIKSMYETMGLKTGLLGTIAYYIHSDHELEAHATTPDAV   420
His_Avi_PaMurE-SATD_J6        PSQKLAVIGITGTNGKTTTSYLIKSMYETMGLKTGLLGTIAYYIHSDHELEAHATTPDAV   207
                              **:**:*:*************::*:**:***:.****** *** ..::***. ***:*:
```

```
                                    1                                      2
PpMurE-TP_Avi_His_D7        NLQNLMASMVQNGTEVCIMEVSSHGLVLGRCEDIEFDVAVFTNLTRDHMDFHKTEEEYRR  251
PaMurE-SATD_Avi_His_E7      YVQKLMAKMVHNGTEACVMEASSHALVQGRCDKVDFDIAVFMNLTRDHMDFHKTEEDYRE  224
His_Avi_LgMurE-SATD_B2      YVQKLMAKMVYNGTEACVMEASSHALMLGRCDKVDFDIAVFTNLTRDHMDFHKTEEDYRA  267
His_Avi_PaMurE-TP_B1        YVQKLMAKMVHNGTEACVMEASSHALVQGRCDKVDFDIAVFMNLTRDHMDFHKTEEDYRE  480
His_Avi_PaMurE-SATD_J6      YVQKLMAKMVHNGTEACVMEASSHALVQGRCDKVDFDIAVFMNLTRDHMDFHKTEEDYRE  267
                             :*:***.** ****.*:**.***.*: ***:.::**:*** ***********:** 

                                           3
PpMurE-TP_Avi_His_D7        AKGLLFAKMVDPERQRKVVNIDDPNVSYFVSQGNQDVPVVTFGMGDKSADVYPLAVKLSL  311
PaMurE-SATD_Avi_His_E7      AKGRLFAKMVDPERHRKVVNIDDPNAPYFIRQGNPDVPVITFSMGNKNADVYALEVQLSL  284
His_Avi_LgMurE-SATD_B2      AKGKLFAKMVDPERHRKVVNIDDPNAPYFIAQGNPDVPVITFAMGNKNADVYALEVQLSL  327
His_Avi_PaMurE-TP_B1        AKGRLFAKMVDPERHRKVVNIDDPNAPYFIRQGNPDVPVITFSMGNKNADVYALEVQLSL  540
His_Avi_PaMurE-SATD_J6      AKGRLFAKMVDPERHRKVVNIDDPNAPYFIRQGNPDVPVITFSMGNKNADVYALEVQLSL  327
                            *** **********:**********. **: *** ****:**.**:*.**** * *:***

PpMurE-TP_Avi_His_D7        VESEVLVRTPQGDVEISSRLLGRHNVYNILTAVAVGIAVGAPLEDIVRGIEAVDAVPGRC  371
PaMurE-SATD_Avi_His_E7      FETQVLIRTSIGNLEISSGLLGRHNVYNILAAVAVGIAVGAPLDDIVKGIEEVDAIPGRC  344
His_Avi_LgMurE-SATD_B2      FETQVLIRTPKGNLEISSGLLGGHNVYNILAAVAVGIAVGAPLDDIVKGIEEVDAIPGRC  387
His_Avi_PaMurE-TP_B1        FETQVLIRTSIGNLEISSGLLGRHNVYNILAAVAVGIAVGAPLDDIVKGIEEVDAIPGRC  600
His_Avi_PaMurE-SATD_J6      FETQVLIRTSIGNLEISSGLLGRHNVYNILAAVAVGIAVGAPLDDIVKGIEEVDAIPGRC  387
                            .*::**:**  *::**** *** *******:************:***:*** ***:****
                                                                                 2
                                                                         4
PpMurE-TP_Avi_His_D7        ELIDEGQTFAVLVDYAHTPDAVARLLDTVRECGPKRIITVLGCGGDRDKGKRPIMAKIAA  431
PaMurE-SATD_Avi_His_E7      ELIDEEQAFAVIVDYAHTPDALARLLDTVRECGAQRIITVVGCGGDRDRGKRPMMAKIAT  404
His_Avi_LgMurE-SATD_B2      ELIDEEQAFAVIVDYAHTPDALARLLDTVRECGAQRVITVVGCGGDRDRGKGPMMAKIAT  447
His_Avi_PaMurE-TP_B1        ELIDEEQAFAVIVDYAHTPDALARLLDTVRECGAQRIITVVGCGGDRDRGKRPMMAKIAT  660
His_Avi_PaMurE-SATD_J6      ELIDEEQAFAVIVDYAHTPDALARLLDTVRECGAQRIITVVGCGGDRDRGKRPMMAKIAT  447
                            ***** *:***:*********:***********  :*:***:*******:** *:*****:

PpMurE-TP_Avi_His_D7        DKSDVCIITSDNPRTEKPLDIIDDMLAGVGWSMEQYCKWEEDSSYPLLPNGHRLFCQEIR  491
PaMurE-SATD_Avi_His_E7      DKSDICILTSDNPRTEDPLDILDDMLAGVGWTMQDYLRWGDDDYYPPLPNGHRLFVYDIR  464
His_Avi_LgMurE-SATD_B2      DKSDICILTSDNPRTEDPLDILDDMLAGVGWTMQDYLRWGDDDYYPPLPNGHRLFVYDIR  507
His_Avi_PaMurE-TP_B1        DKSDICILTSDNPRTEDPLDILDDMLAGVGWTMQDYLRWGDDDYYPPLPNGHRLFVYDIR  720
His_Avi_PaMurE-SATD_J6      DKSDICILTSDNPRTEDPLDILDDMLAGVGWTMQDYLRWGDDDYYPPLPNGHRLFVYDIR  507
                            ****:**:********.****:*********:*::* :* :*. ** ********   :**

                                 5
PpMurE-TP_Avi_His_D7        SKAIRAAVAMAEEGDAVVIAGKGHETYQIIGEIKGHFDDREECREALRL-----------  540
PaMurE-SATD_Avi_His_E7      SIAIRAGVAMGEEGDAVVIAGKGHETYQIVGKQEEYFDDREECREALQKVDALHAAGIDT  524
His_Avi_LgMurE-SATD_B2      SIAIRAGVAMGEEGDAVVIAGKGHETYQIVGKEEEYFDDREECREALQKVDALHAAGIDT  567
His_Avi_PaMurE-TP_B1        SIAIRAGVAMGEEGDAVVIAGKGHETYQIVGKQEEYFDDREECREALQKVDALHAAGIDT  780
His_Avi_PaMurE-SATD_J6      SIAIRAGVAMGEEGDAVVIAGKGHETYQIVGKQEEYFDDREECREALQKVDALHAAGIDT  567
                            * ****.***.*****************:*: : :***********:

PpMurE-TP_Avi_His_D7        ---------RKGALENLYFQSGGLNDIFEAQKIEWHEGLEVLFQGPGKHHHHHH      585
PaMurE-SATD_Avi_His_E7      SEFPWRVPESHGALENLYFQSGGLNDIFEAQKIEWHEGLEVLFQGPGKHHHHHH      578
His_Avi_LgMurE-SATD_B2      SEFPWRVPESH-------------------------------------------      578
His_Avi_PaMurE-TP_B1        SEFPWRVPESH-------------------------------------------      791
His_Avi_PaMurE-SATD_J6      SEFPWRVPESH-------------------------------------------      578
                                      :
```

(b) Table of post-translational modifications to baculovirus-expressed proteins
Data for each box derive from several, often overlapping, fragments within the boxed area.

Red boxed area	Protein		Modification (percentage of TIC)					Total TIC
			carbamylated	ubiquitinylated	methylated	acetylated	phosphorylated	
1	His_Avi_PaMurE-TP	B1	0.00	0.57	9.93	0.00	1.91	18,549,404
	PpMurE-TP_Avi_His	D7	3.24	0.00	3.22	0.00	12.30	27,114,894
	PaMurE-SATD_Avi_His	E7	0.08	0.00	16.44	0.00	2.67	45,134,531
	His_Avi_PaMurE-SATD	J6	0.11	0.00	0.70	0.00	3.41	130,161,962
	His_Avi_LgMurE-TP	B2	n/a	n/a	n/a	n/a	n/a	0
2	His_Avi_PaMurE-TP	B1	0.44	0.00	6.40	0.00	0.00	24,399,914
	PpMurE-TP_Avi_His	D7	2.36	0.00	13.37	0.00	0.00	44,862,548
	PaMurE-SATD_Avi_His	E7	2.92	0.00	10.60	0.00	0.00	34,601,390
	His_Avi_PaMurE-SATD	J6	2.94	0.00	9.51	0.00	0.00	72,925,407
	His_Avi_LgMurE-TP	B2	0.00	0.00	0.00	0.00	0.00	1,777,561
3	His_Avi_PaMurE-TP	B1	0.51	0.58	1.28	0.00	0.00	14,482,474
	PpMurE-TP_Avi_His	D7	3.45	0.18	8.96	0.00	1.87	50,487,838
	PaMurE-SATD_Avi_His	E7	1.29	1.15	4.38	0.00	0.00	17,596,880
	His_Avi_PaMurE-SATD	J6	0.58	0.44	1.69	0.00	0.00	26,157,727
	His_Avi_LgMurE-TP	B2	0.00	0.00	0.00	0.00	0.00	861,049
4	His_Avi_PaMurE-TP	B1	0.00	0.44	15.82	0.00	0.00	8,438,416
	PpMurE-TP_Avi_His	D7	0.50	0.34	48.23	0.00	0.64	33,572,489
	PaMurE-SATD_Avi_His	E7	0.35	0.09	15.40	0.00	0.00	17,398,883
	His_Avi_PaMurE-SATD	J6	0.29	0.19	23.21	0.00	0.00	26,536,581
	His_Avi_LgMurE-TP	B2	0.00	0.00	0.00	0.00	0.00	733,135
5	His_Avi_PaMurE-TP	B1	0.00	0.14	19.37	0.00	0.00	28,194,671
	PpMurE-TP_Avi_His	D7	0.36	0.09	5.96	0.00	0.03	181,432,148
	PaMurE-SATD_Avi_His	E7	0.00	0.21	23.82	0.00	0.00	53,308,571
	His_Avi_PaMurE-SATD	J6	0.22	0.13	15.82	0.00	0.26	170,754,794
	His_Avi_LgMurE-TP	B2	0.00	0.00	0.00	0.00	0.00	4,044,302

(c) Alignment of proteins from *E. coli.*

```
His_AnMurE_46T               ------------------------------------------------------------  0
His_PpMurE-TP_22B            ------------------------------------------------------------  0
PpMurE-TP_Avi_His_1.1T       ------------------------------------------------------------  0
His_LgMurE-TP_G              --------------------------------------------MRGSHHHHHHGSQFTA  16
LgMurE-SATD_Avi_His_7.3T     ------------------------------------------------------------  0
PaMurE-TP_Avi_His_E6         ------------------------------------------------------------  0
PaMurE-SATD_Avi_His_E7       ------------------------------------------------------------  0
His_PaMurE-SATD_H            ------------------------------------------------------------  0
His_Avi_PaMurE-TP_B1         MAHHHHHHSSGLEVLFQGPGGLNDIFEAQKIEWHEGENLYFQSGSAYPSRGFISRIQFTA  60
His_Avi_PaMurE-SATD_J6       MAHHHHHHSSGLEVLFQGPGGLNDIFEAQKIEWHEGENLYFQSGT---------------  45

His_AnMurE_46T               ------------------------------------------------------------  0
His_PpMurE-TP_22B            ------------------------------------------------------------  0
PpMurE-TP_Avi_His_1.1T       ------------------------------------------------------------  0
His_LgMurE-TP_G              RSSGSNGNGNFFPNPAEEEPPEVSEDDYAGLTKFARVETAAARARKKEAEEYERDKHVFL  76
LgMurE-SATD_Avi_His_7.3T     ------------------------------------------------------------  0
PaMurE-TP_Avi_His_E6         --------------MAEEEPPEVLEDDYVGLTKFARVETEAARARKKETEEYERDKHVFL  46
PaMurE-SATD_Avi_His_E7       ------------------------------------------------------------  0
His_PaMurE-SATD_H            ------------------------------------------------------------  0
His_Avi_PaMurE-TP_B1         RNSGSNGSGNFFPNPAEEEPPEVLEDDYVGLTKFARVETEAARARKKETEEYERDKHVFL  120
His_Avi_PaMurE-SATD_J6       ------------------------------------------------------------  45

His_AnMurE_46T               ------------------------------------------------------------  0
His_PpMurE-TP_22B            ------------------------------------------------------------  0
PpMurE-TP_Avi_His_1.1T       ------------------------------------------------------------  0
His_LgMurE-TP_G              SAIGLDENETPVKDEEEGPEIVRLDGNQDSGDFFTEVDKAVALRRKELIKEGSLKPRTAK  136
LgMurE-SATD_Avi_His_7.3T     ------------------------------------------------------------  0
PaMurE-TP_Avi_His_E6         SAIGLDEDETPVKDEEEGPEIVRLDGNQDDGDFFTEVDKAVALRRKELIKEGSLKPRAAK  106
PaMurE-SATD_Avi_His_E7       ------------------------------------------------------------  0
His_PaMurE-SATD_H            ------------------------------------------------------------  0
His_Avi_PaMurE-TP_B1         SAIGLDEDETPVKDEEEGPEIVRLDGNQDDGDFFTEVDKAVALRRKELIKEGSLKPRAAK  180
His_Avi_PaMurE-SATD_J6       ------------------------------------------------------------  45

His_AnMurE_46T               -----MSYYHHHHHHDYDIPTTENLYFQGAMKL---------------------------  28
His_PpMurE-TP_22B            -----MSYYHHHHHHDYDIPTTENLYFQGAMLKMGFG----------------DSKLTDR  39
PpMurE-TP_Avi_His_1.1T       ------------------------------MLKMGFG----------------DSKLTDR  14
His_LgMurE-TP_G              KPPEKEIVDNLEKEEVIDLDEIRALQDKNKLKEDTVGDEEEEPKEEDEESEGTSPARDDD  196
LgMurE-SATD_Avi_His_7.3T     ------------------------------------------------------------  0
PaMurE-TP_Avi_His_E6         KPQEKEIVDDLEKEEVIDLDEIRALQDKNIVEL-----KEEEEQKEDEESEGTSSAADDD  161
PaMurE-SATD_Avi_His_E7       ------------------------------------------------------------  0
His_PaMurE-SATD_H            ------------------------------------------------------------  0
His_Avi_PaMurE-TP_B1         KPQEKEIVDDLEKEEVIDLDEIRALQDKNIVEL-----KEEEEQKEDEESEGTSSAADDD  235
His_Avi_PaMurE-SATD_J6       ------------------------------------------------------------  45

His_AnMurE_46T               -------------------------RELLATVDSVENLPPVLADAEVKGIKTNSHACGAG  63
His_PpMurE-TP_22B            SFSLKSST---HEEAVLDQTDRMTLRKLLNEARVSPLSTEGDLDVEITGIQQDSRLVAPG  96
PpMurE-TP_Avi_His_1.1T       SFSLKSST---HEEAVLDQTDRMTLRKLLNEARVSPLSTEGDLDVEITGIQQDSRLVAPG  71
His_LgMurE-TP_G              SFELDLSEMELASTQVLEPEFRTTLAELLDDAKVVPVSVDGDLDVEITGIQHDSREVNPR  256
LgMurE-SATD_Avi_His_7.3T     ---------------------MTTLAELLDDAKVVPVSVDGDLDVEITGIQHDSREVNPR  39
PaMurE-TP_Avi_His_E6         SFELDLSGMELASTQVLEPEFRTTLAELLDDAKVVPVSVDGDLDVEITGIQHDSREVNPR  221
PaMurE-SATD_Avi_His_E7       ---------------------MTTLAELLDDAKVVPVSVDGDLDVEITGIQHDSREVNPR  39
His_PaMurE-SATD_H            ----------MRGSHHHHHHGSTTLAELLDDAKVVPVSVDGDLDVEITGIQHDSREVNPR  50
His_Avi_PaMurE-TP_B1         SFELDLSGMELASTQVLEPEFRTTLAELLDDAKVVPVSVDGDLDVEITGIQHDSREVNPR  295
His_Avi_PaMurE-SATD_J6       -----------------------TLAELLDDAKVVPVSVDGDLDVEITGIQHDSREVNPR  82
                                                       :**  .           *.*:.**: :*:

His_AnMurE_46T               DLFIGMPGTRVDGGEFWPSAIASGAIAAIVSPQAVEKNPPHDEAVVISSNNMTKACAAIA  123
His_PpMurE-TP_22B            DLFVCVKGLKSDGHQFAIQAIEKGAVAIISLMEVSLTEG--LKAAVI-VEDTSVILSALA  153
PpMurE-TP_Avi_His_1.1T       DLFVCVKGLKSDGHQFAIQAIEKGAVAIISLMEVSLTEG--LKAAVI-VEDTSVILSALA  128
His_LgMurE-TP_G              DLFICCTGMTTDGHLYLTEAIKRGAVAVIASKEITLDESISVKAMVM-VEDGNAILAALA  315
LgMurE-SATD_Avi_His_7.3T     DLFICCTGMTTDGHLYLTEAIKRGAVAVIASKEITLDESISVKAMVM-VEDGNAILAALA  98
PaMurE-TP_Avi_His_E6         DLFICCTGMTTDGHLYLSEAVKRGAVAIIASKEITIDESINVKAMVM-VEDGNTILAALA  280
PaMurE-SATD_Avi_His_E7       DLFICCTGMTTDGHLYLSEAVKRGAVAIIASKEITIDESINVKAMVM-VEDGNTILAALA  98
His_PaMurE-SATD_H            DLFICCTGMTTDGHLYLSEAVKRGAVAIIASKEITIDESINVKAMVM-VEDGNTILAALA  109
His_Avi_PaMurE-TP_B1         DLFICCTGMTTDGHLYLSEAVKRGAVAIIASKEITIDESINVKAMVM-VEDGNTILAALA  354
His_Avi_PaMurE-SATD_J6       DLFICCTGMTTDGHLYLSEAVKRGAVAIIASKEITIDESINVKAMVM-VEDGNTILAALA  141
                             ***:   *   **  :  .*:  **:* *   :    :    :* *:  :: .   :*:*

                                       1  3 and 4
His_AnMurE_46T               AAFYGYPGQKLKLVGVTGTNGKTTTTHLIEFFLTKAKLSTALMGTLYTR-WPGFEQTATH  182
His_PpMurE-TP_22B            GVIYGHPSKKLSVVGITGTNGKTTTSYLLQSLYEAMGLQVGLLGTIQYYIGGKNKLEADH  213
PpMurE-TP_Avi_His_1.1T       GVIYGHPSKKLSVVGITGTNGKTTTSYLLQSLYEAMGLQVGLLGTIQYYIGGKNKLEADH  188
His_LgMurE-TP_G              ASFYGNPSQKLAVIGITGTNGKTTTSYLIKSMYETMGLKTGLLGTIAYYIHSDHELEAHS  375
LgMurE-SATD_Avi_His_7.3T     ASFYGNPSQKLAVIGITGTNGKTTTSYLIKSMYETMGLKTGLLGTIAYYIHSDHELEAHS  158
PaMurE-TP_Avi_His_E6         ASFYGNPSQKLAVIGITGTNGKTTTSYLIKSMYETMGLKTGLLGTIAYYIHSDHELEAHA  340
PaMurE-SATD_Avi_His_E7       ASFYGNPSQKLAVIGITGTNGKTTTSYLIKSMYETMGLKTGLLGTIAYYIHSDHELEAHA  158
His_PaMurE-SATD_H            ASFYGNPSQKLAVIGITGTNGKTTTSYLIKSMYETMGLKTGLLGTIAYYIHSDHELEAHA  169
His_Avi_PaMurE-TP_B1         ASFYGNPSQKLAVIGITGTNGKTTTSYLIKSMYETMGLKTGLLGTIAYYIHSDHELEAHA  414
His_Avi_PaMurE-SATD_J6       ASFYGNPSQKLAVIGITGTNGKTTTSYLIKSMYETMGLKTGLLGTIAYYIHSDHELEAHA  201
                             . :** *.:**  ::*:*********::*:: :     *...*:**:        :  *
```

```
                                     2    5                           3
His_AnMurE_46T                  TTPFAVELQQQLAQAVNAGCEFGVMEVSSHALAQGRVLGCPFEVGVFSNLTQDHLDYHSD  242
His_PpMurE-TP_22B               TTPEALNLQNLMASMVQNGTEVCIMEVSSHGLVLGRCEDIEFDVAVFTNLTRDHMDFHKT  273
PpMurE-TP_Avi_His_1.1T          TTPEALNLQNLMASMVQNGTEVCIMEVSSHGLVLGRCEDIEFDVAVFTNLTRDHMDFHKT  248
His_LgMurE-TP_G                 TTPDAVYVQKLMAKMVYNGTEACVMEASSHALMLGRCDKVDFDIAVFTNLTRDHMDFHKT  435
LgMurE-SATD_Avi_His_7.3T        TTPDAVYVQKLMAKMVYNGTEACVMEASSHALMLGRCDKVDFDIAVFTNLTRDHMDFHKT  218
PaMurE-TP_Avi_His_E6            TTPDAVYVQKLMAKMVHNGTEACVMEASSHALVQGRCDKVDFDIAVFMNLTRDHMDFHKT  400
PaMurE-SATD_Avi_His_E7          TTPDAVYVQKLMAKMVHNGTEACVMEASSHALVQGRCDKVDFDIAVFMNLTRDHMDFHKT  218
His_PaMurE-SATD_H               TTPDAVYVQKLMAKMVHNGTEACVMEASSHALVQGRCDKVDFDIAVFMNLTRDHMDFHKT  229
His_Avi_PaMurE-TP_B1            TTPDAVYVQKLMAKMVHNGTEACVMEASSHALVQGRCDKVDFDIAVFMNLTRDHMDFHKT  474
His_Avi_PaMurE-SATD_J6          TTPDAVYVQKLMAKMVHNGTEACVMEASSHALVQGRCDKVDFDIAVFMNLTRDHMDFHKT  261
                                *** *: :*: :*. *   * *   :**.***.*   **      *::.** ***:**:*:*.

                                      6
His_AnMurE_46T                  MEDYFAAKALLFSPEYL--KGRAIINADDTYGQRLIKALSP-EKVWSYSVNDSSADLWMS  299
His_PpMurE-TP_22B               EEEYRRAKGLLFAKMVDPERQRKVVNIDDPNVSYFVSQGNQDVPVVTFGMGDKSADVYPL  333
PpMurE-TP_Avi_His_1.1T          EEEYRRAKGLLFAKMVDPERQRKVVNIDDPNVSYFVSQGNQDVPVVTFGMGDKSADVYPL  308
His_LgMurE-TP_G                 EEDYRAAKGKLFAKMVDPERHRKVVNIDDPNAPYFIAQGNPDVPVITFAMGNKNADVYAL  495
LgMurE-SATD_Avi_His_7.3T        EEDYRAAKGKLFAKMVDPERHRKVVNIDDPNAPYFIAQGNPDVPVITFAMGNKNADVYAL  278
PaMurE-TP_Avi_His_E6            EEDYREAKGRLFAKMVDPERHRKVVNIDDPNAPYFIRQGNPDVPVITFSMGNKNADVYAL  460
PaMurE-SATD_Avi_His_E7          EEDYREAKGRLFAKMVDPERHRKVVNIDDPNAPYFIRQGNPDVPVITFSMGNKNADVYAL  278
His_PaMurE-SATD_H               EEDYREAKGRLFAKMVDPERHRKVVNIDDPNAPYFIRQGNPDVPVITFSMGNKNADVYAL  289
His_Avi_PaMurE-TP_B1            EEDYREAKGRLFAKMVDPERHRKVVNIDDPNAPYFIRQGNPDVPVITFSMGNKNADVYAL  534
His_Avi_PaMurE-SATD_J6          EEDYREAKGRLFAKMVDPERHRKVVNIDDPNAPYFIRQGNPDVPVITFSMGNKNADVYAL  321
                                 *:*   **. **:        : * ::* **      ::    .     * ::.:.:..**::

His_AnMurE_46T                  DLSYEPNGVTGTIHTPEGNVSFRSPLVGQYNLENLLAAVGAVLHLGLNLQLIANAIPEFP  359
His_PpMurE-TP_22B               AVKLSLVESEVLVRTPQGDVEISSRLLGRHNVYNILTAVAVGIAVGAPLEDIVRGIEAVD  393
PpMurE-TP_Avi_His_1.1T          AVKLSLVESEVLVRTPQGDVEISSRLLGRHNVYNILTAVAVGIAVGAPLEDIVRGIEAVD  368
His_LgMurE-TP_G                 EVQLSLFETQVLIRTPKGNLEISSGLLGGHNVYNILAAVAVGIAVGAPLDDIVKGIEEVD  555
LgMurE-SATD_Avi_His_7.3T        EVQLSLFETQVLIRTPKGNLEISSGLLGGHNVYNILAAVAVGIAVGAPLDDIVKGIEEVD  338
PaMurE-TP_Avi_His_E6            EVQLSLFETQVLIRTSIGNLEISSGLLGRHNVYNILAAVAVGIAVGAPLDDIVKGIEEVD  520
PaMurE-SATD_Avi_His_E7          EVQLSLFETQVLIRTSIGNLEISSGLLGRHNVYNILAAVAVGIAVGAPLDDIVKGIEEVD  338
His_PaMurE-SATD_H               EVQLSLFETQVLIRTSIGNLEISSGLLGRHNVYNILAAVAVGIAVGAPLDDIVKGIEEVD  349
His_Avi_PaMurE-TP_B1            EVQLSLFETQVLIRTSIGNLEISSGLLGRHNVYNILAAVAVGIAVGAPLDDIVKGIEEVD  594
His_Avi_PaMurE-SATD_J6          EVQLSLFETQVLIRTSIGNLEISSGLLGRHNVYNILAAVAVGIAVGAPLDDIVKGIEEVD  381
                                 :. .        ::*  *::.: * *:* :*: *:*:**.. : :*  *: *...*  .

                                                                                        4
His_AnMurE_46T                  GVPGRMERVQINPDQDISVIVDYAHTPDSLENLLKAARPFIPGRMICVFGCGGDRDRTKR  419
His_PpMurE-TP_22B               AVPGRCEL--IDEGQTFAVLVDYAHTPDAVARLLDTVRECGPKRIITVLGCGGDRDKGKR  451
PpMurE-TP_Avi_His_1.1T          AVPGRCEL--IDEGQTFAVLVDYAHTPDAVARLLDTVRECGPKRIITVLGCGGDRDKGKR  426
His_LgMurE-TP_G                 AIPGRCEL--IDEEQAFAVIVDYAHTPDALARLLDTVRECGAQRVITVVGCGGDRDRGKG  613
LgMurE-SATD_Avi_His_7.3T        AIPGRCEL--IDEEQAFAVIVDYAHTPDALARLLDTVRECGAQRVITVVGCGGDRDRGKG  396
PaMurE-TP_Avi_His_E6            AIPGRCEL--IDEEQAFAVIVDYAHTPDALARLLDTVRECGAQRIITVVGCGGDRDRGKR  578
PaMurE-SATD_Avi_His_E7          AIPGRCEL--IDEEQAFAVIVDYAHTPDALARLLDTVRECGAQRIITVVGCGGDRDRGKR  396
His_PaMurE-SATD_H               AIPGRCEL--IDEEQAFAVIVDYAHTPDALARLLDTVRECGAQRIITVVGCGGDRDRGKR  407
His_Avi_PaMurE-TP_B1            AIPGRCEL--IDEEQAFAVIVDYAHTPDALARLLDTVRECGAQRIITVVGCGGDRDRGKR  652
His_Avi_PaMurE-SATD_J6          AIPGRCEL--IDEEQAFAVIVDYAHTPDALARLLDTVRECGAQRIITVVGCGGDRDRGKR  439
                                .:*** *    *:   * ::*:*********:: .**.:.*      *:* *.*******: *

                                       7 and 8
His_AnMurE_46T                  PKMGKIVAELADLAFVTSDNPRTEDPDRILDDILAGIPDTVQP-----------------  462
His_PpMurE-TP_22B               PIMAKIAADKSDVCIITSDNPRTEKPLDIIDDMLAGVGWSMEQYCKWEEDSSYPLLPNGH  511
PpMurE-TP_Avi_His_1.1T          PIMAKIAADKSDVCIITSDNPRTEKPLDIIDDMLAGVGWSMEQYCKWEEDSSYPLLPNGH  486
His_LgMurE-TP_G                 PMMAKIATDKSDICILTSDNPRTEDPLDILDDMLAGVGWTMQDYLRWGDDDYYPPLPNGH  673
LgMurE-SATD_Avi_His_7.3T        PMMAKIATDKSDICILTSDNPRTEDPLDILDDMLAGVGWTMQDYLRWGDDDYYPPLPNGH  456
PaMurE-TP_Avi_His_E6            PMMAKIATDKSDICILTSDNPRTEDPLDILDDMLAGVGWTMQDYLRWGDDDYYPPLPNGH  638
PaMurE-SATD_Avi_His_E7          PMMAKIATDKSDICILTSDNPRTEDPLDILDDMLAGVGWTMQDYLRWGDDDYYPPLPNGH  456
His_PaMurE-SATD_H               PMMAKIATDKSDICILTSDNPRTEDPLDILDDMLAGVGWTMQDYLRWGDDDYYPPLPNGH  467
His_Avi_PaMurE-TP_B1            PMMAKIATDKSDICILTSDNPRTEDPLDILDDMLAGVGWTMQDYLRWGDDDYYPPLPNGH  712
His_Avi_PaMurE-SATD_J6          PMMAKIATDKSDICILTSDNPRTEDPLDILDDMLAGVGWTMQDYLRWGDDDYYPPLPNGH  499
                                * *.**.:: :*:.::*********.*  *:**:***:  :::

                                            5
His_AnMurE_46T                  --TVIGDRAIAIRTAILQAQPGDGVLLAGKGHEDYQILGTEKIHFDDREHARAALTEREK  520
His_PpMurE-TP_22B               RLFCQEIRSKAIRAAVAMAEEGDAVVIAGKGHETYQIIGEIKGHFDDREECREALRL---  568
PpMurE-TP_Avi_His_1.1T          RLFCQEIRSKAIRAAVAMAEEGDAVVIAGKGHETYQIIGEIKGHFDDREECREALRL---  543
His_LgMurE-TP_G                 RLFVYDIRSIAIRAGVAMGEEGDAVVIAGKGHETYQIVGKEEEYFDDREECREALQKVDA  733
LgMurE-SATD_Avi_His_7.3T        RLFVYDIRSIAIRAGVAMGEEGDAVVIAGKGHETYQIVGKEEEYFDDREECREALQKVDA  516
PaMurE-TP_Avi_His_E6            RLFVYDIRSIAIRAGVAMGEEGDAVVIAGKGHETYQIVGKQEEYFDDREECREALQKVDA  698
PaMurE-SATD_Avi_His_E7          RLFVYDIRSIAIRAGVAMGEEGDAVVIAGKGHETYQIVGKQEEYFDDREECREALQKVDA  516
His_PaMurE-SATD_H               RLFVYDIRSIAIRAGVAMGEEGDAVVIAGKGHETYQIVGKQEEYFDDREECREALQKVDA  527
His_Avi_PaMurE-TP_B1            RLFVYDIRSIAIRAGVAMGEEGDAVVIAGKGHETYQIVGKQEEYFDDREECREALQKVDA  772
His_Avi_PaMurE-SATD_J6          RLFVYDIRSIAIRAGVAMGEEGDAVVIAGKGHETYQIVGKQEEYFDDREECREALQKVDA  559
                                       *: ***:.:  .: **.*::****** ***:*  : :*****..* **

His_AnMurE_46T                  L-----------------------------------------------------------  521
His_PpMurE-TP_22B               -----------------RK-----------------------------------------  570
PpMurE-TP_Avi_His_1.1T          -----------------RKGALENLYFQSGGLNDIFEAQKIEWHEGLEVLFQGPGHHHHH  586
His_LgMurE-TP_G                 LHAAGIDTSEFPWRVPESH-----------------------------------------  752
LgMurE-SATD_Avi_His_7.3T        LHAAGIDTSEFPWRVPESHAALENLYFQSGGLNDIFEAQKIEWHEGLEVLFQGPGHHHHH  576
PaMurE-TP_Avi_His_E6            LHAAGIDTSEFPWRVPESHGALENLYFQSGGLNDIFEAQKIEWHEGLEVLFQGPGKHHHH  758
PaMurE-SATD_Avi_His_E7          LHAAGIDTSEFPWRVPESHGALENLYFQSGGLNDIFEAQKIEWHEGLEVLFQGPGKHHHH  576
His_PaMurE-SATD_H               LHAAGIDTSEFPWRVPESH-----------------------------------------  546
His_Avi_PaMurE-TP_B1            LHAAGIDTSEFPWRVPESH-----------------------------------------  791
His_Avi_PaMurE-SATD_J6          LHAAGIDTSEFPWRVPESH-----------------------------------------  578
```

(d) Table of post-translational modifications to *E. coli* proteins.
Data for each box derive from several, often overlapping, fragments within the boxed area. For box 3 the figures in parenthesis represent only the fragments that include the carbamylated lysine for AnMurE and PpMurE. The equivalent fragment for PaMurE and LgMurE was either not found or very rare and unmodified, so not included here

Red boxed area	Protein		Modification (percentage of TIC)					Total TIC
			carbamylated	ubiquitinylated	methylated	acetylated	phosphorylated	
1	His_AnMurE	46T	1.24	0.00	2.23	2.15	7.09	40,291,337
	His_PpMurE-TP	22B	0.00	0.00	0.00	0.00	0.00	17,418,724
	PpMurE-TP_Avi_His	1.1T	0.00	0.00	0.00	0.47	0.00	28,182,466
	His_LgMurE-TP	G	0.00	0.00	0.00	0.00	0.00	2,973,211
	LgMurE-SATD_Avi_His	7.3T	0.00	0.47	0.76	0.96	1.11	9,581,569
	PaMurE-TP_Avi_His	E6	0.00	0.00	0.00	0.00	3.73	3,737,999
	PaMurE-SATD_Avi_His	E7	0.71	0.00	0.26	0.52	4.61	19,507,174
	His_PaMurE-SATD	H	0.90	0.00	0.46	3.97	7.39	24,929,017
	His_Avi_PaMurE-TP	B1	0.50	0.00	0.12	0.43	0.29	22,243,317
	His_Avi_PaMurE-SATD	J6	0.45	0.00	0.26	0.44	2.98	12,037,921
2	His_AnMurE	46T	0.00	0.00	0.00	0.00	0.00	5,686,701
	His_PpMurE-TP	22B	2.36	0.00	8.62	0.00	0.00	7,246,762
	PpMurE-TP_Avi_His	1.1T	0.00	0.00	0.60	0.00	0.00	10,907,645
	His_LgMurE-TP	G	0.00	0.00	6.82	0.00	49.40	809,081
	LgMurE-SATD_Avi_His	7.3T	0.00	0.00	0.00	0.00	82.09	7,577,736
	PaMurE-TP_Avi_His	E6	0.00	0.00	2.13	0.00	10.43	3,828,564
	PaMurE-SATD_Avi_His	E7	0.51	3.53	6.37	0.72	47.18	33,693,334
	His_PaMurE-SATD	H	0.74	0.31	1.09	2.59	37.62	48,604,113
	His_Avi_PaMurE-TP	B1	0.34	4.70	1.57	0.00	38.87	25,554,108
	His_Avi_PaMurE-SATD	J6	0.00	4.77	2.07	0.00	21.67	19,690,949
3	His_AnMurE	46T	0.47 (0.65)	0.00 (0.00)	18.73 (25.90)	0.57 (0.21)	0.00 (0.00)	35,904,173
	His_PpMurE-TP	22B	0.44 (0.00)	0.00 (0.00)	3.17 (0.00)	1.89 (36.12)	0.00 (0.00)	36,364,881
	PpMurE-TP_Avi_His	1.1T	0.00 (0.00)	0.00 (0.00)	3.68 (0.00)	6.05 (71.68)	0.00 (0.00)	35,750,697
	His_LgMurE-TP	G	0.00	0.00	3.56	0.55	0.00	4,686,479
	LgMurE-SATD_Avi_His	7.3T	0.21	0.56	2.60	0.97	0.00	9,626,237
	PaMurE-TP_Avi_His	E6	0.00	0.00	3.92	0.00	0.00	3,731,886
	PaMurE-SATD_Avi_His	E7	1.01	0.00	8.47	1.80	0.00	22,297,802
	His_PaMurE-SATD	H	0.51	0.00	4.86	2.50	0.00	20,685,939
	His_Avi_PaMurE-TP	B1	0.24	0.00	5.94	1.63	0.00	20,325,511
	His_Avi_PaMurE-SATD	J6	0.00	0.00	5.01	2.21	0.00	14,260,445
4	His_AnMurE	46T	0.08	0.40	1.11	1.63	0.00	27,224,656
	His_PpMurE-TP	22B	0.29	0.24	20.00	3.68	0.00	27,273,182
	PpMurE-TP_Avi_His	1.1T	0.26	0.42	7.49	3.02	0.10	30,101,084
	His_LgMurE-TP	G	0.00	0.96	7.09	0.91	0.00	1,949,424
	LgMurE-SATD_Avi_His	7.3T	0.73	21.81	7.07	0.70	0.64	4,502,805
	PaMurE-TP_Avi_His	E6	0.00	3.26	2.99	18.31	0.00	1,991,638
	PaMurE-SATD_Avi_His	E7	2.96	8.94	7.47	46.16	1.35	14,786,534
	His_PaMurE-SATD	H	1.18	17.45	4.24	28.32	1.34	21,443,938
	His_Avi_PaMurE-TP	B1	3.11	9.51	7.49	48.10	0.26	11,390,367
	His_Avi_PaMurE-SATD	J6	2.01	9.17	6.30	34.09	1.55	9,253,909
5	His_AnMurE	46T	0.53	1.14	3.70	2.81	0.19	62,758,765
	His_PpMurE-TP	22B	0.03	0.04	1.03	0.51	0.00	54,369,927
	PpMurE-TP_Avi_His	1.1T	0.09	0.06	1.28	0.33	0.06	265,232,184
	His_LgMurE-TP	G	0.00	0.00	3.18	0.00	0.00	11,900,306
	LgMurE-SATD_Avi_His	7.3T	0.00	0.00	3.64	0.14	0.00	54,875,426
	PaMurE-TP_Avi_His	E6	0.00	0.00	2.27	0.00	0.00	13,664,869
	PaMurE-SATD_Avi_His	E7	0.25	0.00	5.48	0.41	0.06	102,157,729
	His_PaMurE-SATD	H	0.05	0.00	0.78	0.87	0.00	80,422,377
	His_Avi_PaMurE-TP	B1	0.00	0.00	1.28	0.44	0.00	55,701,062
	His_Avi_PaMurE-SATD	J6	0.00	0.00	1.60	0.31	0.00	47,394,727

(e) MS/MS spectra for post-translational modifications to MurE ligases expressed in either baculovirus-infected *S. frugiperda* cells (grey boxes 1 and 2 in (a)) or *E. coli* (grey boxes 3-8 in (c)), and detailed in the text boxes below.

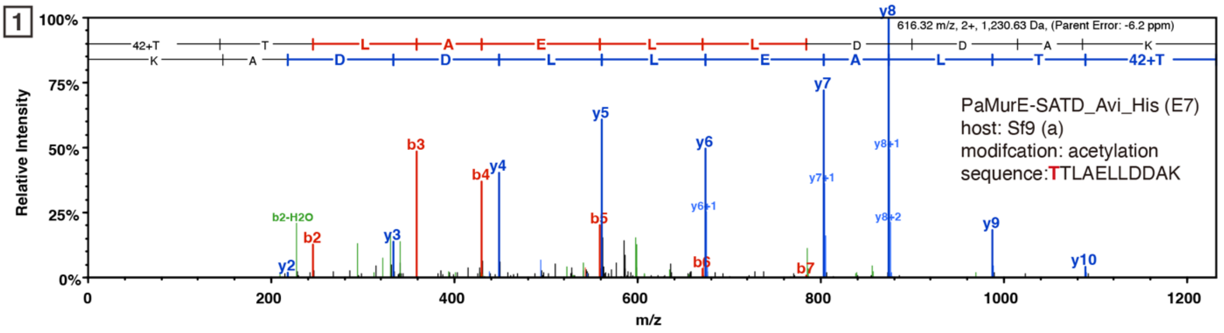

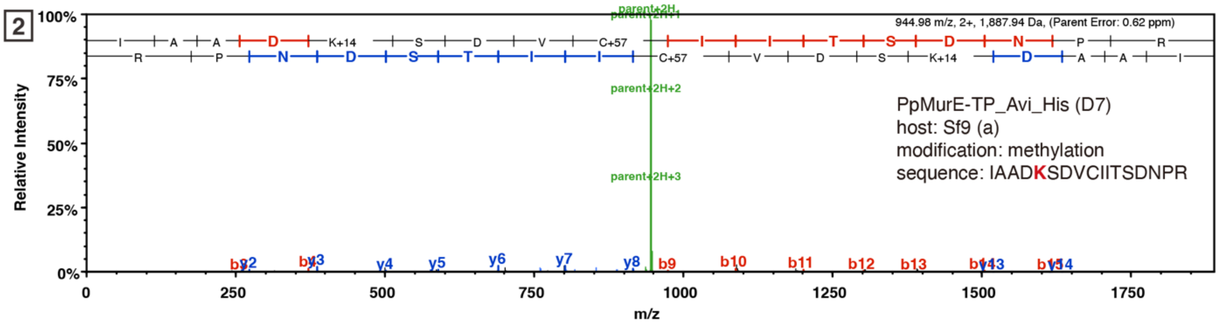

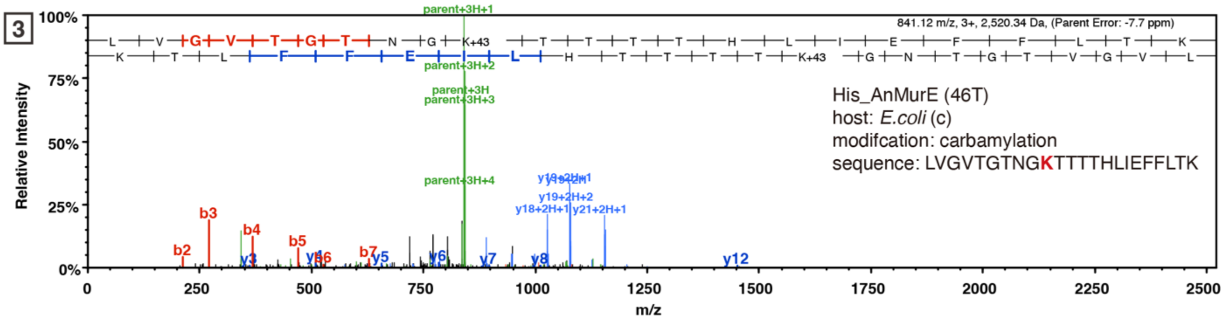

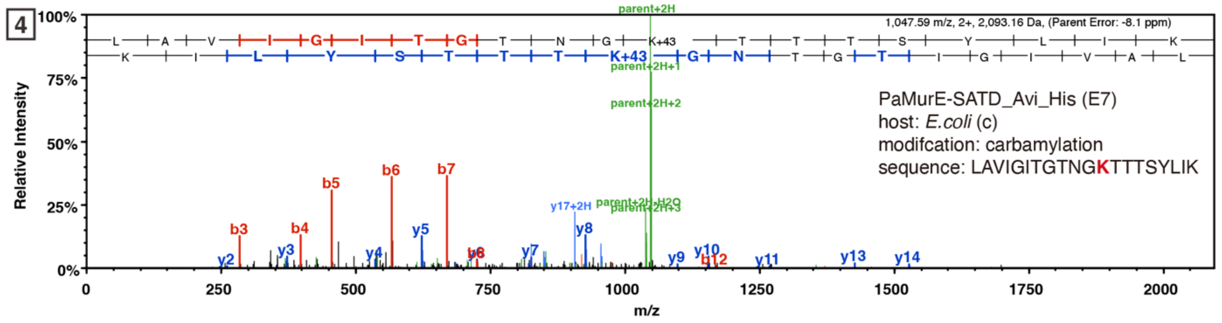

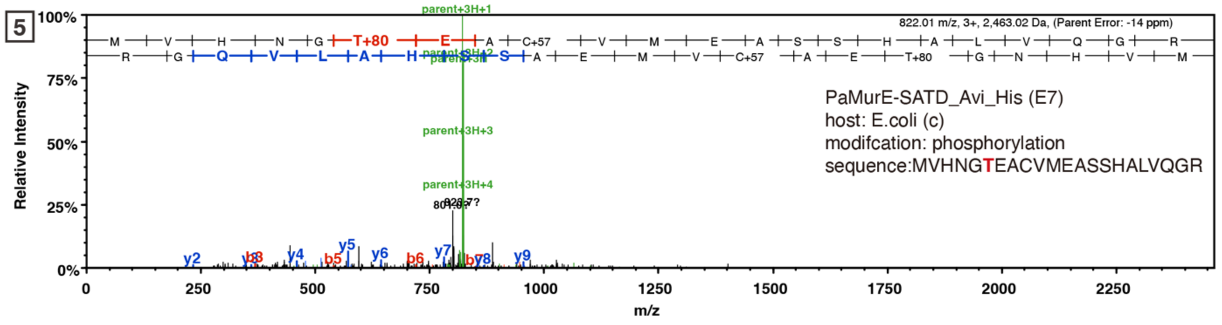

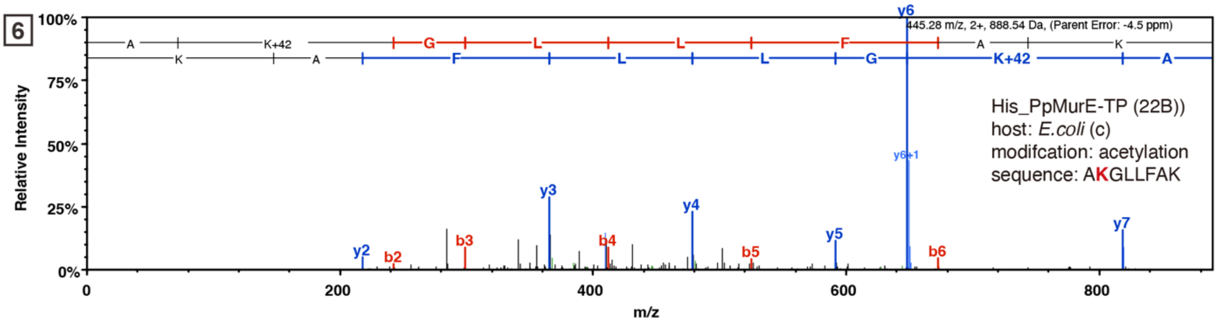
6
445.28 m/z, 2+, 888.54 Da, (Parent Error: -4.5 ppm)
His_PpMurE-TP (22B))
host: E.coli (c)
modifcation: acetylation
sequence: AKGLLFAK
Relative Intensity
m/z
y2
b2
b3
y3
b4
y4
b5
y5
y6
y6+1
b6
y7

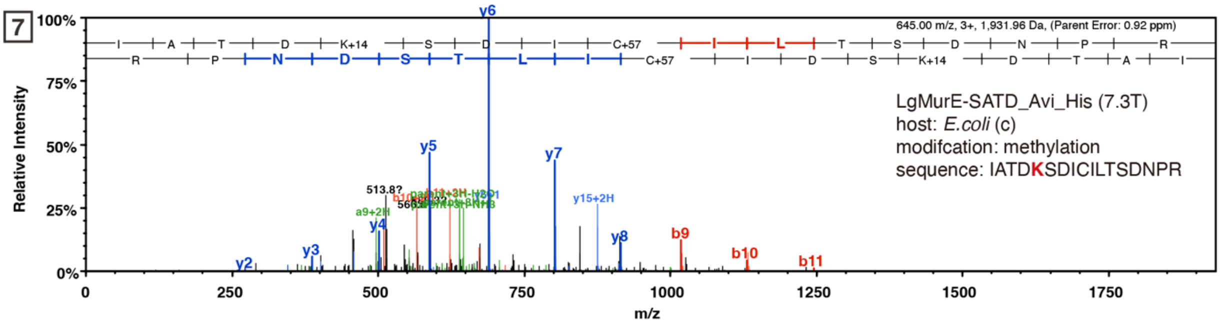
7
645.00 m/z, 3+, 1,931.96 Da, (Parent Error: 0.92 ppm)
LgMurE-SATD_Avi_His (7.3T)
host: E.coli (c)
modifcation: methylation
sequence: IATDKSDICILTSDNPR
Relative Intensity
m/z
y2
y3
y4
a9+2H
513.8?
y5
y6
y7
y15+2H
y8
b9
b10
b11

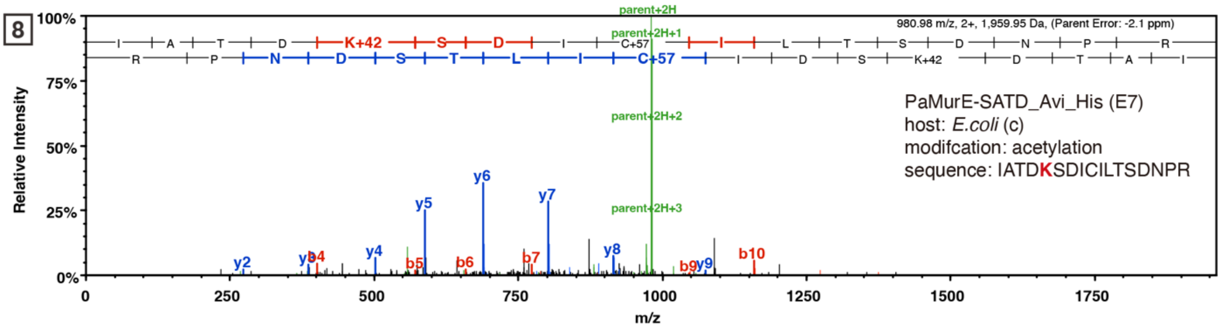
8
980.98 m/z, 2+, 1,959.95 Da, (Parent Error: -2.1 ppm)
PaMurE-SATD_Avi_His (E7)
host: E.coli (c)
modifcation: acetylation
sequence: IATDKSDICILTSDNPR
Relative Intensity
m/z
parent+2H
parent+2H+1
parent+2H+2
parent+2H+3
y2
b4
y4
b5
y5
b6
y6
b7
y7
y8
b9
y9
b10

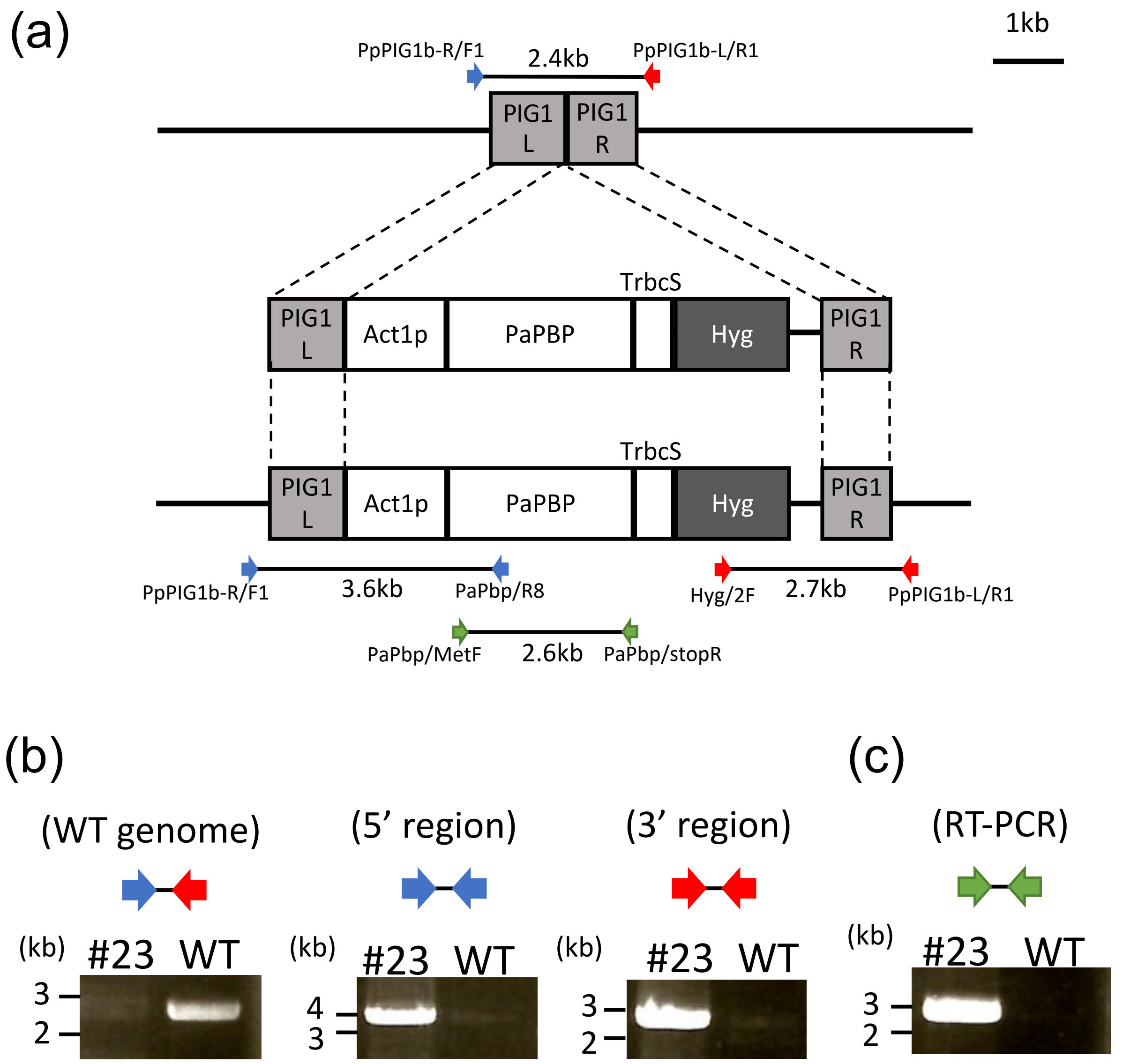

Figure S8. Generation of stable transformants expressing *PaPBP* gene in Δ*PpPbp*.
(a) Schematic representation of the PIG1 genomic region in the WT (top) and *PaPBP* transgenic plants (bottom); a part of the plasmid used for generating transformants is shown between the two. PIG1L, left half of the PIG1 sequence; PIG1R, right half of PIG1; Act1p, rice actin promoter; TrbcS, terminator of the pea rbcS gene; Hyg, hygromycin resistance gene cassette. (b) Genomic PCR for screening of transformants. Primers used were shown in (a). (c) Expression of *PaPbp* in wild-type and transformants was examined by RT-PCR with the primers (green) shown in (a).
